# An actionable axis linking NFATc2 to EZH2 controls the EMT-like program of melanoma cells

**DOI:** 10.1038/s41388-019-0729-2

**Published:** 2019-02-01

**Authors:** Valentina Perotti, Paola Baldassari, Alessandra Molla, Gabriella Nicolini, Ilaria Bersani, Giulia Grazia, Fabio Benigni, Andrea Maurichi, Mario Santinami, Andrea Anichini, Roberta Mortarini

**Affiliations:** 1 Department of Research, Human Tumors Immunobiology Unit, Milan, Italy; 2 HuMabs Biomed, a subsidiary of Vir Biotechnology, Bellinzona, Switzerland; 3 Melanoma and Sarcoma Unit, Department of Surgery, Fondazione IRCCS Istituto Nazionale dei Tumori, Milan, Italy

**Keywords:** Melanoma, Apoptosis

## Abstract

Discovery of new actionable targets and functional networks in melanoma is an urgent need as only a fraction of metastatic patients achieves durable clinical benefit by targeted therapy or immunotherapy approaches. Here we show that NFATc2 expression is associated with an EMT-like transcriptional program and with an invasive melanoma phenotype, as shown by analysis of melanoma cell lines at the mRNA and protein levels, interrogation of the TCGA melanoma dataset and characterization of melanoma lesions by immunohistochemistry. Gene silencing or pharmacological inhibition of NFATc2 downregulated EMT-related genes and AXL, and suppressed c-Myc, FOXM1, and EZH2. Targeting of c-Myc suppressed FOXM1 and EZH2, while targeting of FOXM1 suppressed EZH2. Inhibition of c-Myc, or FOXM1, or EZH2 downregulated EMT-related gene expression, upregulated MITF and suppressed migratory and invasive activity of neoplastic cells. Stable silencing of NFATc2 impaired melanoma cell proliferation in vitro and tumor growth in vivo in SCID mice. In NFATc2^+^ EZH2^+^ melanoma cell lines pharmacological co-targeting of NFATc2 and EZH2 exerted strong anti-proliferative and pro-apoptotic activity, irrespective of BRAF or NRAS mutations and of BRAF inhibitor resistance. These results provide preclinical evidence for a role of NFATc2 in shaping the EMT-like melanoma phenotype and reveal a targetable vulnerability associated with NFATc2 and EZH2 expression in melanoma cells belonging to different mutational subsets.

## Introduction

Several master genes play a key role in regulation of complex transcriptional networks that shape the biological behavior of cutaneous melanoma [1–5]. Oncogenes, different classes of transcription factors (TF) and epigenetic regulators have been associated to regulation of proliferation, epithelial-mesenchymal transition (EMT)-like programs, invasive activity, development of metastasis, and resistance to target-specific inhibitors [5]. BRAF and NRAS oncogenes were shown to fuel a switch in the EMT-TFs network from ZEB2/SNAIL2 in favor of ZEB1/TWIST1, leading to promotion of invasive properties [4]. Master transcription factors SOX10/MITF and AP-1/TEADS play a crucial role in the control of the proliferative and invasive transcriptional networks, respectively [3], while c-JUN, a component of AP-1 [6], is a mediator of the mesenchymal-like profile of melanoma cells [2]. The epigenetic regulators RNF2 and EZH2 promote the invasive, metastatic and EMT-like phenotype of melanoma cells [1, 7]. Melanomas characterized by the invasive transcriptional program, associated with high expression of AXL, show intrinsic resistance to BRAF and ERK inhibitors [8]. Moreover, in response to targeted therapy, or to inflammatory signals associated with immunotherapy, melanoma may dedifferentiate along a two-dimensional trajectory including melanocytic, transitory, neural-crest-like, and undifferentiated stages [9]. Interestingly, this process leads also to enhanced susceptibility to ferroptosis-inducing drugs [9]. Collectively, these findings suggest that the identification of master genes associated with melanoma EMT-like phenotype and invasive transcriptional programs may reveal new targetable vulnerabilities.

The TF NFATc2 is frequently expressed and transcriptionally active in cutaneous melanoma, and we found that it may behave as a master gene controlling transcriptional programs of melanoma cells [10]. In fact, NFATc2 targeting reversed melanoma de-differentiation, promoted upregulation of MITF and of melanocyte-lineage-specific antigens, and downregulated the stemness-related marker CD271 [10].

By building upon this initial evidence we tested the hypothesis that NFATc2 could be involved in controlling the EMT-like/invasive melanoma program by regulating a specific set of downstream molecular targets. To this end, we tested the hypothesis that c-Myc, FOXM1, and EZH2 could be among the possible downstream targets based on the following rationale. We knew that Myc is suppressed by NFATc2 targeting in melanoma [10]. FOXM1 is a Myc target gene [11] and is known for playing a major role in the EMT process and metastasis formation [12]. FOXM1 is involved in the transcriptional control of the epigenetic regulator EZH2 [13], the latter gene being also a regulator of the EMT program [14] and of the invasive activity and metastatic ability of melanoma cells [1].

Here we show that the EMT-like transcriptional program of melanoma cells is indeed controlled by NFATc2 acting on c-Myc, FOXM1 and EZH2 and that NFATc2 regulates melanoma migratory and invasive activity in vitro, and tumor growth in vivo. Crucially, pharmacological co-targeting of NFATc2 and EZH2 exerted significant anti-tumor activity not only against BRAF-mutant melanomas with intrinsic resistance to BRAF inhibitors, but even against NRAS-mutant and BRAF/NRAS wild type melanoma cells.

## Results

### NFATc2^+^ melanomas express markers of the EMT-like/invasive transcriptional program

**Fig. 1:**
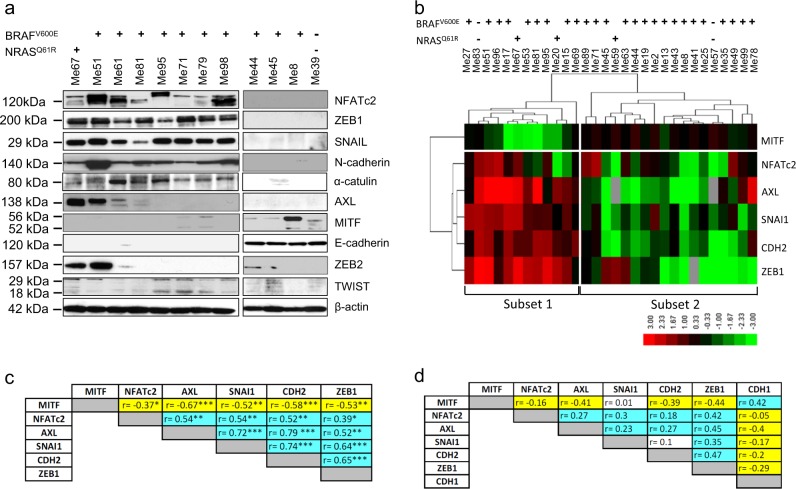
NFATc2 expression correlates with EMT-related markers in melanoma. **a** Western blot analysis for NFATc2, ZEB1, SNAIL, N-cadherin, α-catulin, AXL, MITF, E-cadherin, ZEB2, and TWIST, in melanoma cell lines with the indicated genotype for BRAF^V600E^ or NRAS^Q61R^ mutations. **b** Hierarchical clustering of log_2_-transformed and median-centered qPCR mRNA expression levels (2^−Δ*Ct*^) for MITF, NFATc2, AXL, SNAI1, CDH2, ZEB1 genes in 30 melanoma cell lines with the indicated genotype for BRAF^V600E^ or NRAS^Q61R^ mutations. **c** Spearman correlation analysis of mRNA expression values obtained by qPCR for the indicated genes in the panel of 30 melanoma cells lines shown in **b**. Positive and negative *r-*values are indicated in light blue and yellow, respectively.*p < 0.05; **p < 0.01; ***p < 0.001. **d** Spearman correlation analysis of the indicated genes in the TCGA melanoma dataset; *r*-values indicated as in **c**

By western blot analysis in 12 melanoma cell lines we tested expression of NFATc2, of several EMT-related proteins as well as of AXL and MITF, the prototypic markers of the alternative invasive/proliferative transcriptional programs of melanoma [15]. Among the EMT-related protein tested, the transcription factors ZEB1 and SNAIL, the adhesion molecule N-cadherin and the scaffold molecule α-catulin [5, 16, 17], were expressed only in NFATc2^+^ cell lines (Fig. 1a). Some of the NFATc2^+^ cell lines expressed AXL (Fig. 1a). The epithelial marker E-cadherin and MITF were expressed only in NFATc2^−^ cell lines (Fig. 1a). ZEB2 and TWIST did not prove to be discriminative on the NFATc2^+^ and NFATc2^−^ cell lines.

Expression of NFATc2, MITF, AXL, ZEB1, SNAI1 (encoding SNAIL), and CDH2 (N-Cadherin) was then evaluated at the mRNA level, by qPCR, in a larger panel of 30 melanoma cell lines. Hierarchical clustering of normalized mRNA expression levels identified two subsets of lines. Each subset included BRAF-mutant, NRAS-mutant, and BRAF/NRAS wild-type lines. Subset 1 was characterized by enhanced expression of AXL, ZEB1, SNAI1, CDH2, NFATc2, and lower expression of MITF, compared to subset 2 (Fig. 1b). By Spearman correlation analysis of the qPCR data, NFATc2 showed a direct and significant correlation with AXL, ZEB1, SNAI, and CDH2, while MITF showed a negative correlation with NFATc2 and with all the other investigated genes (Fig. 1c). NFATc2 showed a positive correlation with AXL, SNAI1, CDH2, and ZEB1 even in the TCGA melanoma dataset (Fig. 1d). In the TCGA melanoma dataset, the top 312 genes with a positive correlation (down to Spearman *r* = 0.5) with the prototypic EMT marker ZEB1, were also positively correlated with SNAI1, NFATc2, CDH2, and AXL, but negatively with MITF and CDH1 (Supplementary Table S1).

As shown by Tsoi et al. [9] melanoma differentiation follows a multistage two-dimensional trajectory where four transcriptional subsets reflect the transition from the most differentiated (M = melanocytic) to the most undifferentiated subtype (U = undifferentiated) through two intermediate steps (T = transitory and N = neural crest like). Differential expression of several genes (MITF, SOX10, ERBB3, CDH1, NGFR, SOX9, EGFR, and AXL) occurs along the M → T → N → U transition [9] and we visualized such progressive change in gene expression through principal component analysis (PCA) plots that highlight expression trends of each gene in a cell line dataset (Supplementary Fig. S1a). We then visualized the expression trends of NFATc2, of EMT-related genes (SNAI1, CDH1, ZEB1, CTNNAL1, and CDH2) and of additional genes investigated in this study (FOXM1, EZH2). NFATc2 and SNAI1 showed PCA profiles consistent with higher expression in T and N subsets. ZEB1, CTNNAL1, and CDH2 showed PCA profiles consistent with higher expression in T, N, and U subsets (Supplementary Fig. S1b).

Expression of NFATc2 and of EMT-related markers was then investigated by immunohistochemistry (IHC) in human melanoma lesions. NFATc2^+^ melanomas expressed AXL, N-cadherin and ZEB1, but lacked MITF (Supplementary Fig. S2 and S3). In contrast, MITF^+^ lesions lacked NFATc2, AXL, N-cadherin and ZEB1 (Supplementary Fig. S4 for representative results). Additional metastatic lesions were characterized by IHC and marker expression was subjected to quantitative analysis by the open source QuPath software [18]. In three out of four lesions (Supplementary Fig. S5, S6, S7) the tumor was positive for NFATc2, ZEB1 and N-cadherin, but lacked MITF. In a fourth lesion (Supplementary Fig. S8) the tumor expressed NFATc2 and ZEB1, but was negative for N-Cadherin and expressed MITF. In these lesions NFATc2 was expressed in both nuclear and cytoplasmic compartments of tumor cells (Supplementary Fig. S5a, c, S6a, c, S7a, c, S8a, c).

Finally, to investigate the role of pathways that may be involved in NFATc2 regulation [19, 20] we focused on the ERK pathway, since MEK inhibition has been shown to downregulate NFATc2 gene expression in melanoma cells [20]. Indeed, MEK inhibition by PD0325901 reduced NFATc2 protein expression in two melanoma cell lines (Supplementary Fig. S9).

Taken together, these data indicate that NFATc2 is expressed in melanoma cells showing an EMT-like transcriptional program.

### Targeting of NFATc2 by siRNA, shRNA and by pharmacological inhibition reverses the EMT-like/invasive transcriptional program of melanoma cells

**Fig. 2:**
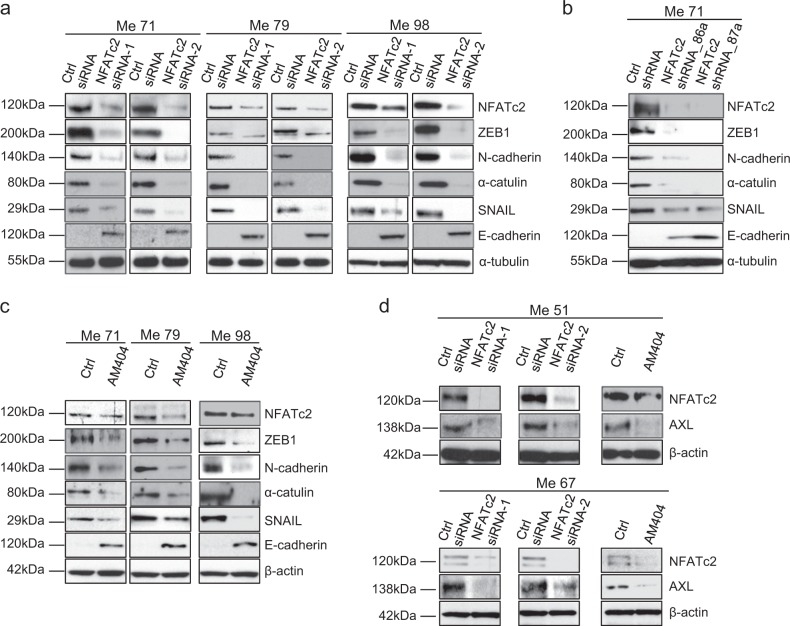
Targeting of NFATc2 in melanoma cells downregulates EMT-related markers and AXL. **a** Expression by western blotting of NFATc2, ZEB1, N-cadherin, α-catulin, SNAIL, and E-cadherin in three melanoma cell lines (Me71, Me79, Me98) at 72 h after transfection with two different NFATc2-specific Stealth siRNA (NFATc2 siRNA-1 and siRNA-2) or with control siRNA (ctrl siRNA). **b**, **c** Expression of NFATc2, ZEB1, N-cadherin, α-catulin, SNAIL, and E-cadherin in **b** NFATc2 shRNA stable transfectants (NFATc2 shRNA_86a and NFATc2 shRNA_87a) or in control cells (ctrl shRNA), or **c** in melanoma cell lines Me71, Me79, and Me98 treated or not for 144 h with the NFATc2 inhibitor AM404 (at 20 μM). **d** Expression of NFATc2 and AXL in melanoma cell lines Me51 (top panels) and Me67 (bottom panels) after NFATc2 targeting by siRNA as in **a**, or by AM404 as in **c**

Targeting of NFATc2 by siRNA in three different melanoma cell lines (Fig. 2a), by shRNA in one cell line (Fig. 2b), and by treatment of three cell lines with AM404, an inhibitor that prevents NFATc2 binding to DNA [21] (Fig. 2c), led to strong downregulation of the EMT-markers ZEB1, N-cadherin, α-catulin and SNAIL, but upregulated E-cadherin, a marker of the mesenchymal to epithelial transition [22]. NFATc2 targeting by siRNA or pharmacological inhibition led also to downregulation of AXL in BRAF-mutant (Me51) and in NRAS-mutant (Me67) cell lines (Fig. 2d). Densitometric analysis of independent replicate experiments of data in Fig. 2 are shown in Supplementary Fig. S10. Modulation of CDH1, EZH2, ZEB1, and SNAI1, upon NFATc2 silencing was confirmed at the mRNA level by qPCR in Me79 cells (Supplementary Fig. S11). Targeting of NFATc2 by shRNA (Supplementary Fig. S12) inhibited several EMT-related and migration/invasion-related genes such as TBX3, ITGB3, NRP1, CTGF, DDR2, FSTL1, AHNAK, and PVRL3 [23–30], as well as of mesenchymal-related genes PRR16 and KDR [31, 32], but increased expression of the epithelial marker DSP [33] and of the pigmentation-related gene QPCT [34]. Taken together these results indicate that NFATc2 controls an EMT-like/invasive melanoma transcriptional program.

### An axis connects NFATc2 to FOXM1 and EZH2, through c-Myc, and controls the EMT-like program of melanoma cells

**Fig. 3:**
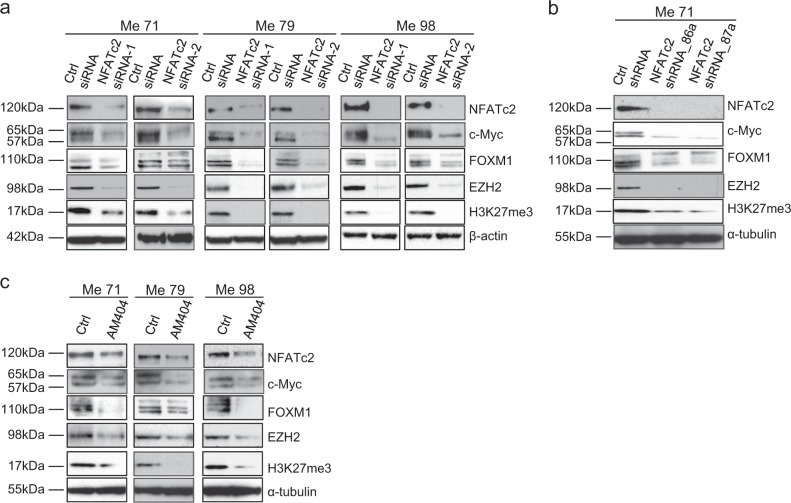
Targeting of NFATc2 in melanoma cells downregulates c-Myc, FOXM1, and EZH2. **a** Expression by western blotting of NFATc2, c-Myc, FOXM1, EZH2, and H3K27me3 in three melanoma cell lines (Me71, Me79, Me98) at 72 h after transfection with two different NFATc2-specific Stealth siRNA (NFATc2 siRNA-1 and siRNA-2) or with control siRNA (ctrl siRNA). **b**, **c** Expression of NFATc2, c-Myc, FOXM1, EZH2, and H3K27me3 in **b** NFATc2 shRNA stable transfectants (NFATc2 shRNA_86a, and NFATc2 shRNA_87a) or in control cells (ctrl shRNA), or **c** in melanoma cell lines Me71, Me79, and Me98 treated or not for 144 h with the NFATc2 inhibitor AM404 (at 20 μM)

**Fig. 4:**
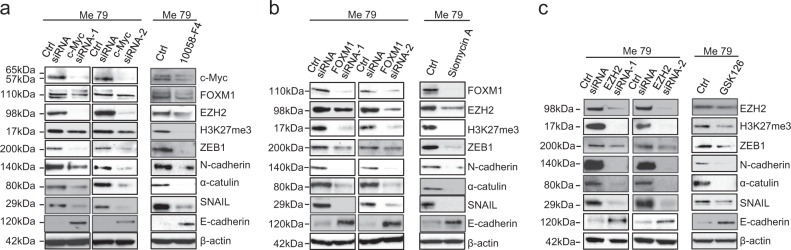
Regulation of FOXM1 and EZH2 by c-Myc, of EZH2 by FOXM1 and downregulation of EMT-related markers by targeting of c-Myc, FOXM1 and EZH2. **a** Expression by western blotting of c-Myc, FOXM1, EZH2, H3K27me3 and of different EMT-related markers in melanoma cell line Me79 at 72 h after transfection with two different c-Myc-specific Stealth siRNA (c-Myc siRNA-1 and siRNA-2) or with control siRNA (ctrl siRNA), or at 48 h after treatment with c-Myc inhibitor 10058-F4 at 20 μM. **b**, **c** expression by western blotting of the indicated proteins after transfection of Me79 at 72 h with two different FOXM1-specific siRNA **b** or with two different EZH2-specific siRNA **c** or with control siRNA, or at 48 h after treatment with FOXM1 inhibitor Siomycin A at 2 μM **b** or with EZH2 inhibitor GSK126 at 5 μM **c**

By whole-genome gene expression analysis of a melanoma transfectant (Me71) with stable knockdown of NFATc2 (NFATc2_shRNA_86a) we observed reduced expression of Myc, FOXM1, and EZH2 compared to two different control transfectants (column entitled “Log ratio” in Supplementary Table S2a,b). These three genes were also predicted to be inhibited upon NFATc2 silencing by the “upstream regulator analysis” carried out by Ingenuity Pathway Analysis (IPA) software (columns entitled “Predicted activation state” and “Activation *Z*-score” in Tables S2a,b). On this basis and according to available evidence on regulation of FOXM1 by Myc and on regulation and roles of FOXM1 and EZH2 in EMT [11–14], we tested the hypothesis that the EMT-like program of melanoma cells could be regulated by an axis connecting NFATc2 to EZH2, through c-Myc and FOXM1. In agreement, targeting of NFATc2 by siRNA in three cell lines (Fig. 3a), by shRNA in one cell line (Fig. 3b), and by pharmacological inhibition with AM404 in three cell lines (Fig. 3c), not only downregulated c-Myc, as expected, but also inhibited FOXM1 and EZH2 and suppressed EZH2 function, as shown by inhibition of trimethylation of histone 3 at lysine 27 (H3K27me3, Fig. 3a–c). Densitometric analysis of independent replicate experiments of data in Fig. 3 are shown in Supplementary Fig. S13. We then sequentially targeted each of the three genes downstream of NFATc2. Silencing of c-Myc by siRNA in three different melanoma cell lines (Fig. 4a and Supplementary Fig. S14a), or treatment of melanoma cells with the c-Myc inhibitor 10058-F4 (Fig. 4a and Supplementary Fig. S14b) downregulated FOXM1, EZH2, and H3K27me3, as well as the EMT-related markers ZEB1, N- Cadherin, α-catulin and SNAIL, but upregulated E-Cadherin. Gene silencing of FOXM1 by siRNA or its pharmacological targeting, with Siomycin A, inhibited EZH2 and H3K27me3, as well as the expression of EMT-markers ZEB1, N-cadherin, α-catulin and SNAIL, but upregulated E-Cadherin (Fig. 4b and Supplementary Fig. S14c,d). Similarly, targeting of EZH2 by siRNA, and by the EZH2-specific inhibitor GSK126, inhibited ZEB1, N-cadherin, α-catulin and SNAIL and upregulated E-cadherin (Fig. 4c and Supplementary Fig. S14 e,f). Modulation of CDH1, ZEB1, and SNAI1, upon EZH2 silencing was confirmed at the mRNA level by qPCR in Me79 cells (Supplementary Fig. S15). Targeting of FOXM1 and of EZH2, by siRNA, led to upregulation of MITF (Supplementary Fig. S16 a, b). Collectively, these results support a model where an NFATc2/c-Myc/FOXM1/EZH2 axis regulates the EMT-like/invasive program of melanoma cells.

### Relevance of the NFATc2 and of downstream targets in regulation of melanoma migration, invasion and growth in vitro and in vivo

**Fig. 5:**
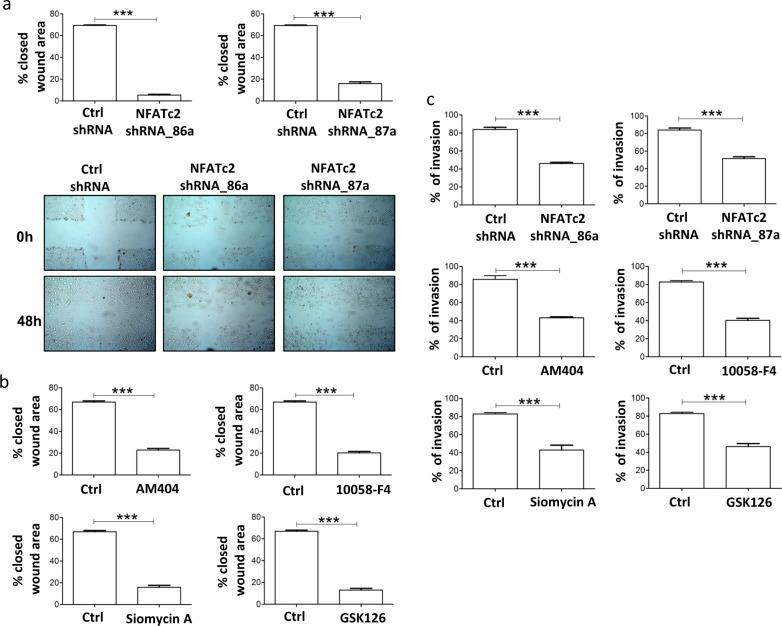
Targeting of NFATc2, Myc, FOXM1, and EZH2 inhibits melanoma migratory and invasive activity. **a** Top panels: reduced melanoma cell migration in NFATc2 shRNA transfectants (NFATc2 shRNA_86a and NFATc2 shRNA_87a) compared to control transfectants (Ctrl_shRNA), evaluated by the wound closure assay. Bottom panels: representative images of the wound closure assay performed with NFATc2 shRNA transfectants and control transfectants. **b** Inhibition of Me71 melanoma migration by the wound closure assay by treatment for 48 h with inhibitors of NFATc2 (AM404), c-Myc (10058-F4), FOXM1 (Siomycin A), or EZH2 (GSK126). Results expressed as % closed wound area. **c** Inhibition of melanoma cell invasive activity, evaluated at 8 h by the CultrexCoat BME Cell Invasion Assay. Top panels: NFATc2 shRNA transfectants compared to control transfectants. Middle and bottom panels: melanoma cell line Me71 pre-treated for 48 h with the indicated inhibitors as in **a**. Statistical analysis in **a**, **b**, **c** by Student's *t*-test. ****p < 0.001. Error bars indicate mean ± SD. Data from four-independent experiments

EMT is characterized by increased migratory and invasive ability of neoplastic cells [16]. NFATc2 silencing by shRNA or treatment of melanoma cells with inhibitors of NFATc2 (AM404), c-Myc (10058-F4), FOXM1 (Siomycin A) or EZH2 (GSK126) reduced significantly migratory activity (Fig. 5a, b), by wound healing assay, and invasive ability (Fig. 5c). NFATc2 shRNA transfectants, kept in low serum (0–2% FCS) showed a significant reduction in growth, compared to control transfectants, in long term (144–168 h) proliferation assays (Supplementary Fig. S17a). Tumor nodules from Me71 melanoma cells with stably silenced NFATc2 showed significant reduction in both volume and weight compared to nodules from control transfectants, at day +55 after s.c. injection in SCID mice (Supplementary Fig. S17b, c). By IHC in tumor nodules, NFATc2 shRNA transfectants lacked expression of NFATc2 and of EZH2, compared to control transfectants (Supplementary Fig. S18).

### Pharmacological co-targeting of NFATc2 and EZH2 induces significant anti-tumor effects in distinct melanoma subsets

**Fig. 6:**
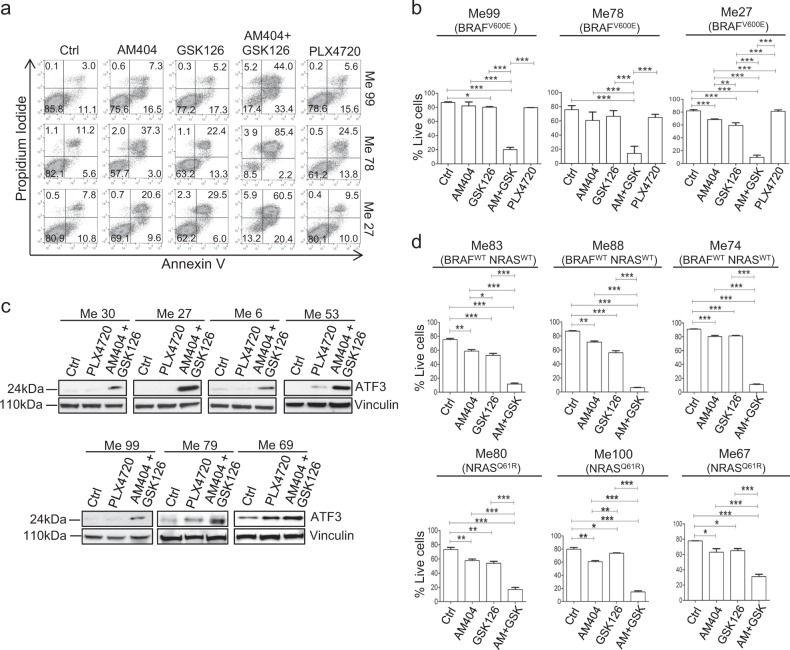
Promotion of melanoma apoptosis and upregulation of ATF3 by combinatorial treatment with NFATc2 and EZH2 inhibitors. **a**, **b** Evaluation of apoptosis by PI/Annexin-V assay on BRAF^V600E^-mutant and BRAF-inhibitor-resistant melanoma cell lines Me99, Me78, and Me27 (PLX4720 IC_50_ values: 5.638 μM, 1.593 μM, 0.582 μM, respectively, as reported in ref. 35), at 48 h after treatment with NFATc2 inhibitor AM404 (15 μM), or EZH2 inhibitor GSK126 (10 μM), or their combination, or the BRAF^V600E^-specific inhibitor PLX4720 (at 1 μM on Me99 and Me78; at 0.25 μM on Me27). Data of a representative experiment shown in **a**, data from three-independent experiments shown in **b**. **c** Expression of ATF3, by western blot analysis, in 7 BRAF-mutant melanoma cell lines, upon treatment o/n with PLX4720 or with the combination of AM404 and GSK126. **d** Promotion of apoptosis, by Annexin-V/PI assay at 48 h, in BRAF/NRAS wild type (top panels) or NRAS^Q61R^-mutant (bottom panels) melanoma cell lines after treatment with NFATc2 inhibitor AM404 (15 μM), or with EZH2 inhibitor GSK126 (10 μM), or with the combination of AM404 and GSK126. Data from three-independent experiments. **b**, **d** results expressed as % live cells (PI^−^ Annexin-V^−^ cells). Statistical analysis in **b**, **d** by one-way ANOVA followed by SNK test; *p < 0.05; **p < 0.01; ***p < 0.001. Error bars indicate mean ± SD

**Fig. 7:**
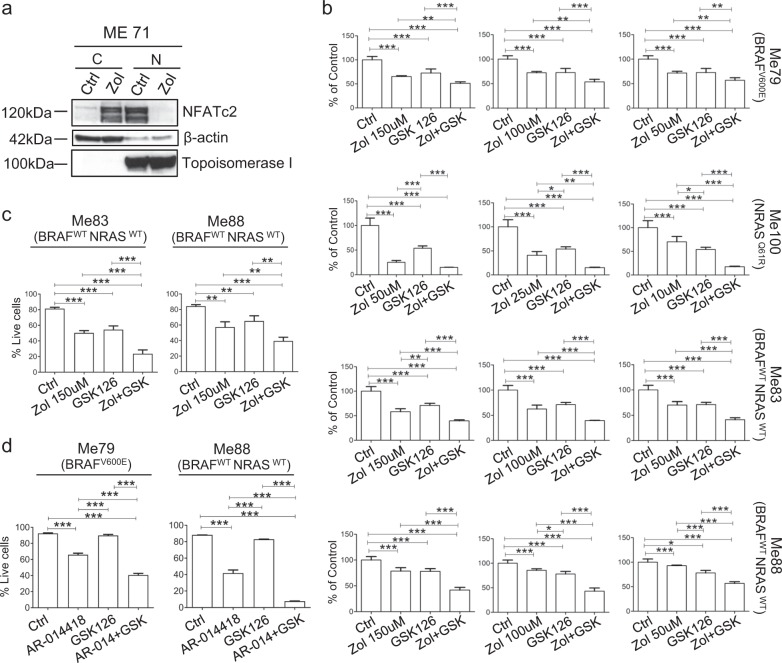
Modulation of subcellular localization of NFATc2 by Zoledronic acid and anti-tumor effect of the combination of Zoledronic acid with GSK126 and of a GSK-3β inhibitor with GSK126. **a** Expression of NFATc2 by western blot analysis in cytoplasmic (C) or nuclear (N) fractions of melanoma cells treated or not with zoledronic acid (indicated as Zol, 150 μM, 72 h). **b** Anti-proliferative effect by MTT assay of Zoledronic acid (at three different doses in each cell line), GSK126 or their combinations on melanoma cell lines belonging to distinct mutational subset. **c** Apoptosis by annexin-V/PI assay at 72 h in melanoma cells treated with Zoledronic acid, GSK126 or their combinations. **d** Apoptosis by annexin-V/PI assay at 72 h in melanoma cells treated for 48 h with GSK-3β inhibitor AR-014418 (at 7.5 μM) and GSK126 (10 μM) or their combinations. Data from three-independent experiments. Statistical analysis in **b**, **c**, **d** by ANOVA and SNK post test. *p < 0.05; **p < 0.001, ***p < 0.001. Error bars indicate mean ± SD

Melanoma cell lines belonging to distinct mutational groups (BRAF-mutant, NRAS-mutant, or wild type for both BRAF and NRAS, Supplementary Fig. S19a top and bottom panels) co-expressed NFATc2 and EZH2 supporting the rationale for testing the potential anti-tumor effects of pharmacological co-targeting. We first tested the EZH2 inhibitor GSK126, either used alone or in association with AM404. The association of AM404 and GSK126 induced a strong pro-apoptotic effect against three BRAFV600E-mutant melanoma cell lines previously characterized [35] for intrinsic resistance to the BRAF-specific inhibitor PLX4720 (Fig. 6a, b). The AM404 + GSK126 was significantly more effective in induction of melanoma cell death compared to the single agents and to PLX4720 (Fig. 6a, b) and these results were confirmed in 6 additional BRAF-mutant cell lines (Supplementary Fig. S19b). Interestingly, the AM404 + GSK126 combination led also to upregulation of ATF3, a candidate melanoma tumor suppressor [36], as evaluated at 18 h (Fig. 6c). Association of AM404 and GSK126 induced a strong pro-apoptotic effect even in BRAF/NRAS wild-type cell lines and in NRAS-mutant melanomas, compared to treatment with single agents (Fig. 6d). The AM404 + GSK126 association induced a significantly increased anti-tumor effect, compared to treatment with single inhibitors, even in a 3D spheroid tumor model (Supplementary Fig. S19c). As a further approach, we used zoledronic acid, a third generation biphosphonate with significant anti-melanoma activity [37] that inhibits the GSK-3β-dependent NFATc2 nuclear stabilization pathway [38]. Treatment of melanoma cells with zoledronic acid suppressed nuclear NFATc2 associated with increased cytoplasmic NFATc2 levels (Fig. 7a). The zoledronic acid + GSK126 association exerted a strong anti-proliferative effect on BRAF-mutant, NRAS-mutant, and BRAF/NRAS wild-type melanoma cell lines (Fig. 7b), compared to treatment with single agents, and induced significant apoptosis on BRAF wild-type melanoma cell lines (Fig. 7c). A significant increase in melanoma apoptosis, compared to treatment with single agents, was obtained even by association of EZH2 inhibitor GSK126 with the GSK-3β inhibitor AR-014418 (Fig. 7d), in agreement with the role of GSK-3β in NFATc2 regulation [39]. Finally, the AM404 + GSK126 combination, after O/N treatment, reversed the EMT-like profile of melanoma cells by reducing SNAIL, ZEB1, AXL, and α-catulin protein expression while upregulating MITF and E-cadherin (Supplementary Fig. S20a). Pre-treatment of melanoma cells with AM404 + GSK126 combination led also to a significant increase in melanoma cell death in response to PLX4720 at 72 h in two BRAF-mutant and PLX4720-resistant melanoma cell lines (Supplementary Fig. S20c). Taken together these results indicate that NFATc2 and EZH2 represent potentially relevant actionable targets in melanomas belonging to different mutational subsets.

## Discussion

EMT-like programs, regulated by multiple signaling pathways, promote melanoma invasive activity [4] and concur to intrinsic and acquired resistance to MAPK inhibitors and to immunotherapy [8]. Thus, identification of master genes and functional networks that promote the EMT-like melanoma transcriptional profile may be crucial to define new therapeutic targets. Our results show that NFATc2 is a relevant regulator of the EMT-like melanoma program. In fact, by analysis of melanoma cell lines, TCGA data and melanoma lesions, we found that NFATc2 defines a subset of melanomas characterized by constitutive expression of a large set of EMT-related genes. In the investigated melanoma lesions NFATc2 expression showed an association with EMT-markers and lack of MITF, but at least one exception was found, in agreement with the notion that EMT and melanoma de-differentiation are multi-step processes with several intermediate phenotypic profiles [9].

Silencing or pharmacological inhibition of NFATc2 downregulated expression of EMT-related genes ZEB1, N-cadherin, α-catulin, and SNAIL, and of several migration/invasion-related and mesenchymal-related genes. Targeting of NFATc2 also suppressed AXL, a marker of the two less differentiated melanoma subsets identified by Tsoi et al. [9]. Silencing of NFATc2, or its pharmacological inhibition not only downregulated c-Myc, in agreement with our previous work [10], but led also to downregulation of the c-Myc target FOXM1 [11] and of the FOXM1 target EZH2 [13]. This evidence suggested that NFATc2 is functionally linked to FOXM1 and EZH2, two genes known to regulate mesenchymal programs in cancer cells. FOXM1 promotes EMT and fosters tumor cell migration and invasion [40]. EZH2, a known EMT regulator in melanoma [14], also promotes EMT in breast cancer [41] and induces migration and invasion of renal cancer cells [42]. In agreement, targeting of FOXM1 and of EZH2, as well as of the upstream regulator c-Myc, induced a reversal of the EMT-like profile of melanoma cells, associated with upregulation of MITF and inhibited melanoma migratory and invasive activity.

In vitro and in vivo melanoma growth were significantly impaired by stable silencing of NFATc2. Tumor nodules of shNFATc2 transfectants removed from SCID mice were negative for EZH2, suggesting that loss of expression of both NFATc2 and EZH2 could contribute to the observed strong anti-tumor effect. Analysis of a panel of melanoma cell lines indicated co-expression of NFATc2 and of EZH2, thus supporting the rationale for testing the potential anti-tumor effects of pharmacological co-targeting. The relevance of targeting the EMT process in melanoma is supported by distinct lines of evidence. Several preclinical studies have shown that small molecule inhibitors targeting BRAF, MEK, PI3K, or VEGFR, used as single agents, or as combinations, can suppress EMT-related biological functions such as migration and invasion [16 for review]. The results of our study corroborate the potential relevance of targeting main regulators of the EMT-like program. This approach downregulated the EMT-like program, reduced migratory and invasive activity and suppressed tumor growth in vitro and in vivo. In addition, inhibition of NFATc2 by AM404 or zoledronic acid, or the GSK-3β inhibitor AR-014418 in association with the EZH2 inhibitor GSK126-induced significant anti-proliferative and pro-apoptotic effects on melanoma cell lines. Interestingly, zoledronic acid has been shown to decrease expression of EMT-markers N-cadherin, Twist and Snail, while upregulating E-cadherin in triple-negative breast cancer cells [43]. Although the calcineurin-NFAT axis has been proposed to be implicated in differentiation of neural crest stem cells [44], current evidence indicates that NFATc2 may not be expressed in normal melanocytes [45], suggesting that the functional axis proposed in this study, and involved in the EMT-like program of melanoma cells, may be activated mainly after neoplastic transformation. Pharmacological co-targeting of NFATc2 and EZH2 exerted significant anti-tumor effects in distinct melanoma subsets, including BRAF-mutant but BRAF-inhibitor-resistant cell lines, as well as NRAS mutant and BRAF/NRAS wild-type tumors.

Taken together these results indicate that the functional axis identified in this study, and linking NFATc2 to EZH2, is not only a relevant regulatory network of the EMT-like program in melanoma cells, but also a potential actionable pathway in different subsets of melanomas, irrespective of BRAF/NRAS mutational status.

## Materials and methods

### Melanoma cell lines and treatments

Melanoma cell lines were established, maintained and routinely tested for the absence of mycoplasma contamination by PCR, as previously described [46]. Lesions were histologically confirmed to be cutaneous malignant melanoma. BRAF or NRAS mutations of the cell lines were described previously [35, 47]. Cell lines were authenticated by short tandem repeat (STR) analysis using the GenePrint10 kit (Promega, Madison, WI, USA) allowing co-amplification and detection of TH01, TPOX, vWA, Amelogenin, CSF1PO, D16S539, D7S820, D13S317, D5S818, and D21S11 loci. Collectively this STR profiling has a random match probability of 1 in 2.92 × 10^9^. The STR profiles of the cell lines were distinct from any cell line reported in the current STR Profile Databases maintained by JCRB (http://cellbank.nibio.go.jp/cellbank_e.html), ATCC (http://www.atcc.org) and DSMZ (http://www.dsmz.de/). Melanoma cell lines were treated for 48–144 h (depending on experiments) with the following inhibitors: AM404, a specific inhibitor that blocks NFATc2 binding to DNA (Enzo Life Sciences, Farmingdale, NY, USA) at 10–20 μM, c-Myc inhibitor 10058-F4 (Sigma-Aldrich, St. Louis, MO, USA) at 20 μM, FOXM1 inhibitor Siomycin A (Enzo Life Sciences) at 1–2 μM, EZH2 inhibitor GSK126 (Selleckchem, Munich, Germany) at 5 μM, BRAFV600E inhibitor PLX4720 at 0.25–1 μM (Selleckchem), the GSK-3β inhibitors Zoledronic acid (Mylan, Canonsburgh, PA, USA) at 10–150 μM or AR0-14418 (Selleckchem) at 7.5 μM, and the MEK inhibitor PD0325901 (Cayman Chemical Company, Ann Arbor, MI) at 100 nM. The study was conducted according to the Declaration of Helsinki Principles, following approval by the independent ethical committee of our Institute, and informed consent was obtained from patients.

### Western blot analysis

SDS-PAGE was performed with 30 μg of protein lysate on 4–12% NuPAGE Bis-Tris (Thermo Fisher Scientific, Waltham, MA, USA), in MOPS buffer as previously described [47]. Primary antibodies (Supplementary Table S3) were diluted in milk 5% or BSA 5% in TBST as described [47] and incubated overnight. Nuclear and cytoplasmic lysates were generated as described [47]. Development was performed with the ECL normal western blot detection System by the chemiluminescence method. Densitometric analysis was carried out by Quantity One software (Bio-Rad Laboratories Inc., Hercules, CA, USA).

### Quantitative RT-PCR

RNA was extracted from melanoma cell lines by TRIzol (Thermo Fisher Scientific) and cDNA was synthesized from 1 μg of RNA using Transcriptor First Strand cDNA synthesis Kit (Roche, Basel, Switzerland), according to the manufacturer instructions. qPCR was carried out using Taqman Gene Expression Assays 20X (Thermo Fisher Scientific, listed in Supplementary Table S4) and Taqman Gene Expression Master Mix 2X (Applied Biosystems, Foster City, CA, USA). qPCR was carried out with 20 ng input complementary DNA, 1 × TaqMan Gene Expression Master Mix and TaqMan Gene Expression Assays on an ABI PRISM 7900 HT thermal cycler (Applied Biosystems). Data were analyzed using ABI PRISM Sequence Detection Software version 2.2.2 (Applied Biosystems). Relative expression was determined using the formula 2^−Δ*Ct*^, reflecting target gene expression normalized to endogenous control genes levels [10].

### Gene silencing by Stealth siRNA and by shRNA

Transient silencing experiments were performed as described [10] with Stealth Select RNAi siRNA oligos (Thermo Fisher Scientific) listed in Supplementary Table S5 and with corresponding recommended Stealth RNAi siRNA-negative controls. Oligos were used at 75–100 nM final concentration according to lipofectamine RNAiMAX guidelines (Thermo Fisher Scientific). In a melanoma cell line (Me71) expressing NFATc2, stable knockdown of NFATc2 was achieved by shRNA retroviral plasmids (SKU TF311198, OriGene, Rockville, MD, USA) as previously described [10].

### TCGA data analysis, gene expression trends in PCA space and whole-genome gene expression analysis

The TCGA melanoma dataset was accessed and interrogated through the tools available in the cBioportal for Cancer Genomics at www.cBioportal.org [48] by looking at the tumor set (*n* = 472) with available mRNA data. The “mRNA co-expression tool” was used to retrieve the list of Spearman correlation values of the mRNA expression levels (RNAseq V2) for the genes investigated in this study (NFATc2, ZEB1, SNAI1, CDH2, AXL, MITF, CDH1, FOXM1, EZH2) with all the genes in the dataset. Gene expression trends by Principal Component Analysis (PCA) plots, based on the cell line dataset, as described by Tsoi et al. [9], were investigated by the interactive web interface resource available at http://systems.crump.ucla.edu/dediff/. Whole-genome gene expression analysis of NFATc2_shRNA_86a and of two control transfectants (control shRNA_1 and _2) was carried out as described (35), by single-color hybridization of RNAs performed on Illumina Bead Chip HumanHT-12_v4 Microarrays (Illumina). The expression profiles have been deposited in NCBI’s Gene Expression Omnibus (GEO) with GSE accession number GSE101323.

### Immunohistochemistry

Immunohistochemistry was performed as described [47] on formalin-fixed, paraffin-embedded (FFPE) tissues from the following samples: primary and metastatic human melanoma samples and neoplastic nodules removed from SCID mice injected s.c. with melanoma cell transfected with NFATc2 shRNA retroviral plasmids or with control plasmids. Sections were stained with antibodies listed in Supplementary Table S6. Images were acquired at ×20 with an Aperio Scanscope XT digital pathology slide scanner (Leica Biosystems, Wetzlar, Germany).

### MTT assay

NFATc2 shRNA and control transfectants were seeded at 8 × 10^3^ cells/well in 96-well culture plates in the presence of increasing concentrations of FCS. After 144 or 168 h, cultures were evaluated as described [47] by the 3-(4, 5) dimethylthiazol-2, 5-diphenyltetrazolium bromide (MTT) assay. Effects of Zoledronic acid, GSK126 and their association on melanoma cell proliferation were tested at 72 h. The absorbance was measured at 570 nm with reference at 630 nm, by using an Infinite 1000 instrument (Tecan, Männedorf, Switzerland).

### Migration and invasion assays

Cell migration was evaluated by the wound healing assay. Confluent cultures in 6-well plates of NFATc2 shRNA transfectants and control transfectants, or of melanoma cell line 71 were wounded using a sterile 200 μl pipette tip, then washed three times. AM404, 10058-F4, Siomycin A, or GSK126 inhibitors were then added and wound closure was assessed at 48 h by imaging wounds at ×5 through an Axiovert 100 microscope (Zeiss, Oberkochen, Germany) equipped with a digital camera (AxioCam MrC5, Zeiss). Wound width was evaluated through the TScratch software [49]. Data were expressed as percentage of closure of the original (*t* = 0 h) wound width. The invasive activity of melanoma cells was tested using the CultrexCoat BME Cell Invasion Assay 96 well (R&D Systems, MN, MN, USA), according to the manufacturer’s instructions. In these experiments melanoma cells were pre-treated for 48 h with different inhibitors as described for the migration assay. Fluorescence signal from calcein-labeled cells was read by an Infinite 1000 instrument (Tecan). Data were expressed as % of invasion by referring to a standard curve.

### Apoptosis assay

Melanoma cell lines were treated for 48–72 h with AM404, GSK126, AR-014418, Zoledronic acid, PLX4720, or with the associations of AM404 + GSK126 or Zoledronic acid + GSK126, or AR-014418 + GSK126, or AM404 + GSK126 + PLX4720. Apoptosis was then evaluated by flow cytometry on FACscalibur instrument (BD, San Diego, CA, USA) after staining cells with APC-conjugated Annexin-V (BD Pharmingen) and propidium iodide (PI, BD Biosciences) as described [47].

### 3-D tumor spheroid assay

Spheroids formation of melanoma cells was tested using the Cultrex® 3-D Spheroid Colorimetric Proliferation/Viability Assay 96 well (R&D Systems, MN, MN, USA), according to the manufacturer’s instructions. Melanoma cells (8 × 10^3^/well) were resuspended in 1X Spheroid Formation medium containing basement membrane proteins. At 48 h, after spheroid formation, cell culture medium containing inhibitors was added. Results were then assessed by the MTT assay after 72 h of treatment with inhibitors.

### In vivo experiments

In vivo experiments in SCID mice were performed according to the Italian laws (D.L. 116/92 and after additions), after approval by the institutional Ethical Committee for Animal Experimentation of our Institute and by the Italian Ministry of Health. Melanoma cells with stable knockdown of NFATc2 (by shRNA) and related control transfectants were injected subcutaneously (5 × 10^6^) in the left flank of 8–10-week-old female SCID mice (Charles River Laboratories, Wilmington, MA, USA). Mice (*n* = 4/group of treatment) were monitored daily for signs of toxicity and were weighed twice weekly. Animals were randomized and allocated to experimental groups based on homogenous distribution of weight. Tumor size was regularly evaluated by measuring the orthogonal diameters (d and D). Neoplastic nodules were removed at day +55 and tumor volume were calculated with the following formula: $\frac{4}{3}{\mathrm{\pi }}\frac{{({\mathrm{d}}^2 \ast {\mathrm{D}})}}{2}.$

### Statistical analysis

mRNA expression levels, resulting from qPCR assays for gene expression in melanoma cell lines, were log_2_-transformed and median-centered and then analyzed by hierarchical clustering by Cluster 3.0 software (University of Tokyo, Japan). Results of clustering were visualized through Java TreeView software [50] and subjected to Spearman correlation analysis through PRISM (GraphPad Software, La Jolla, CA, USA). Expression of EMT-related genes and induction of melanoma apoptosis by AM404, GSK126, AR-014418, Zoledronic acid, or PLX4720 were evaluated by ANOVA followed by SNK multiple comparison test. In vitro growth assays comparing NFATC2 shRNA and control transfectants in different serum concentrations were analyzed by two-way ANOVA and Bonferroni post test. Effects of AM404, 10058-F4, Siomycin A, or GSK126 inhibitors on melanoma cell migration and invasion were evaluated by Student’s *T*-test. Comparison of in vivo growth curves of NFATc2 shRNA and control transfectants was carried out by mixed effects model ANOVA [35] by the XLSTAT software (Xlstat, Addinsoft’s, New York, NY, USA).

## Supplementary information

Supplementary Figure

Supplementary Table
