# Supplementary material for: An actionable axis linking NFATc2 to EZH2 controls the EMT-like program of melanoma cells
Source: Oncogene. 2019 Feb 1;38(22):4384–96. doi: 10.1038/s41388-019-0729-2 (PMC6756060; doi:10.1038/s41388-019-0729-2)
Supplement: Supplementary file 1 — Supplementary Figure [file 41388_2019_729_MOESM1_ESM.pdf]

Supplementary Figure S1

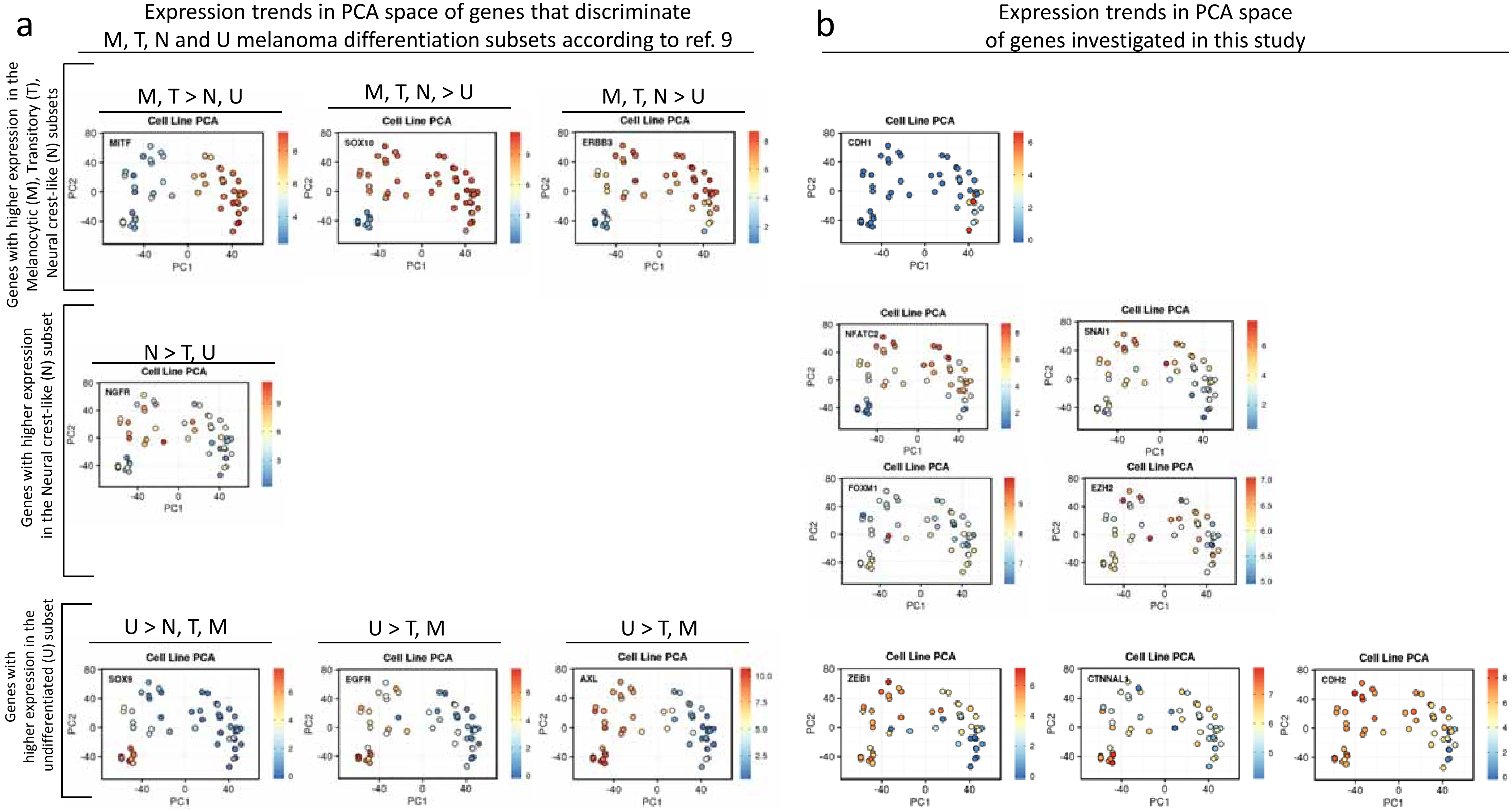

Supplementary Figure S1. a Expression trends in PCA space of representative genes, identified in ref. 9, that discriminate Melanocytic (M), Transitory (T), Neural crest-like (N) and Undifferentiated (U) melanoma subsets. The interactive Web-interface resource available at http://systems.crump.ucla.edu/dediff/ based on the cell line dataset described by Tsoi et al. (9) was used to generate these PCA Plots. In these PCA plots the most differentiated cells, classified as 'Melanocytic' subset, are found on the lower right hand side of the plot, while the most undifferentiated cells, belonging to the 'Undifferentiated' subset are found on the lower left hand side of the plot. 'Transitory' and 'Neural crest-like' subsets are found in the upper right and upper left sides of the plots, respectively. Summary of differential expression for each gene in the four melanoma subsets (as reported in ref.9) is schematically summarized above each PCA plot. Top PCA plots: three genes (MITF, SOX10, ERBB3) with higher expression in Melanocytic and Transistory subsets. Middle panel: a gene (NGFR) with higher expression in the Neural crest-like subset. Bottom panel: three genes (SOX9, EGFR, AXL) with higher expression in the Undifferentiated subset. **b** The same web interface resource mentioned in (a) was used to visualize expression trends in PCA space of relevant genes (CDH1, NFATc2, SNAI1, ZEB1, CTNNAL1, CDH2, FOXM1, EZH2) investigated in this study.

Supplementary Figure S2

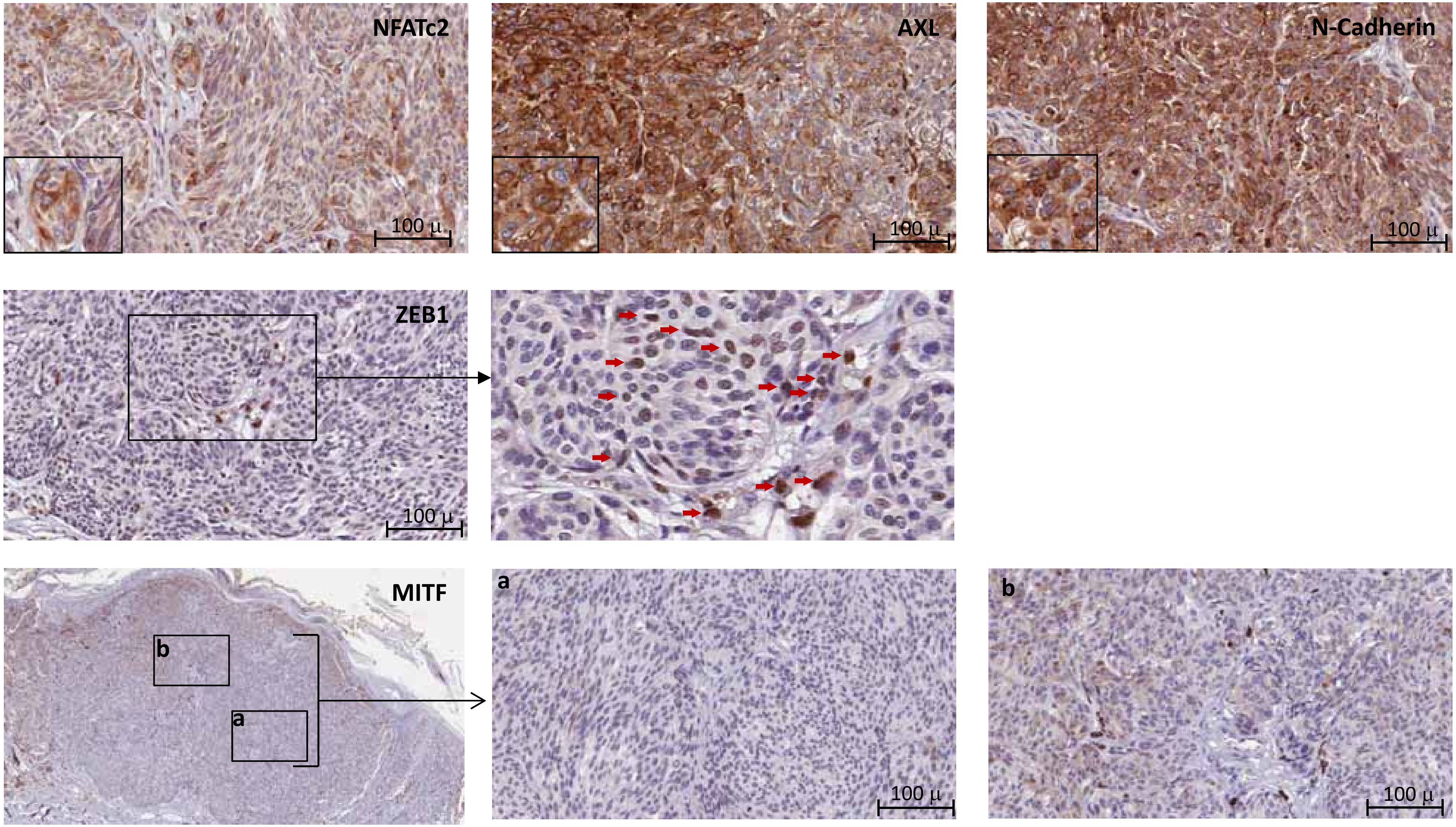

Supplementary Figure S2. **Expression of NFATc2, AXL, N-cadherin, ZEB1 and MITF in a melanoma lesion**. Characterization by immunohistochemistry of a 1.8 mm thick primary lesion, Clark level IV with presence of vertical growth phase, lack of ulceration and of regression. The tumor expresses NFATc2, AXL, and N-cadherin (top panels), ZEB1 (nuclear staining, red arrows in the inset) with heterogeneous expression in neoplastic cells, but lacks MITF (bottom panels). Insets: higher magnification of a representative area.

Supplementary Figure S3

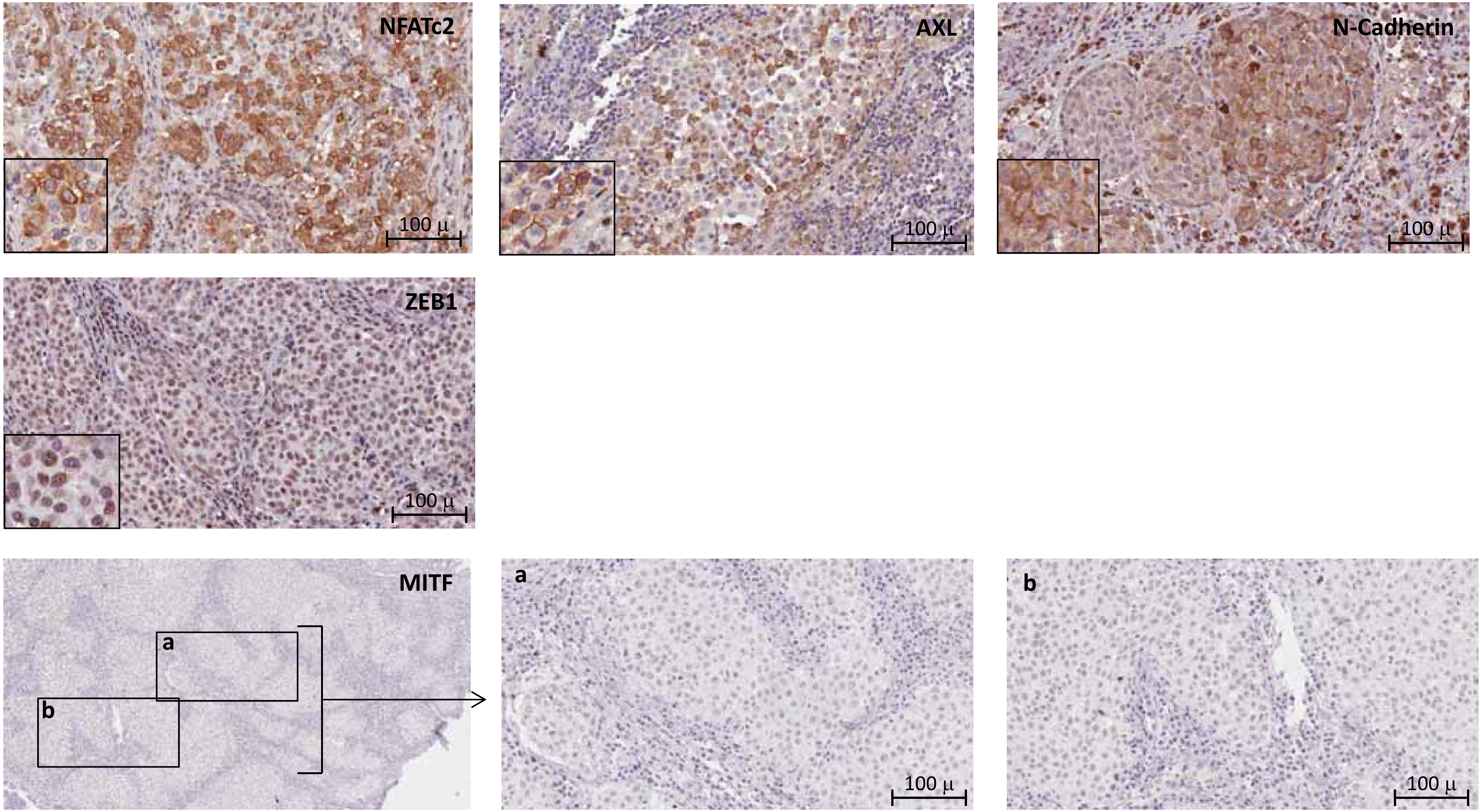

Supplementary Figure S3. **Expression of NFATc2, AXL, N-cadherin, ZEB1 and MITF in a melanoma lesion**. Characterization by immunohistochemistry of a lymph node metastasis of melanoma. The tumor expresses NFATc2, AXL, N-cadherin (top panels) and ZEB1 (nuclear staining, middle panel), but lacks MITF (bottom panels). Insets: higher magnification of a representative area.

Supplementary Figure S4

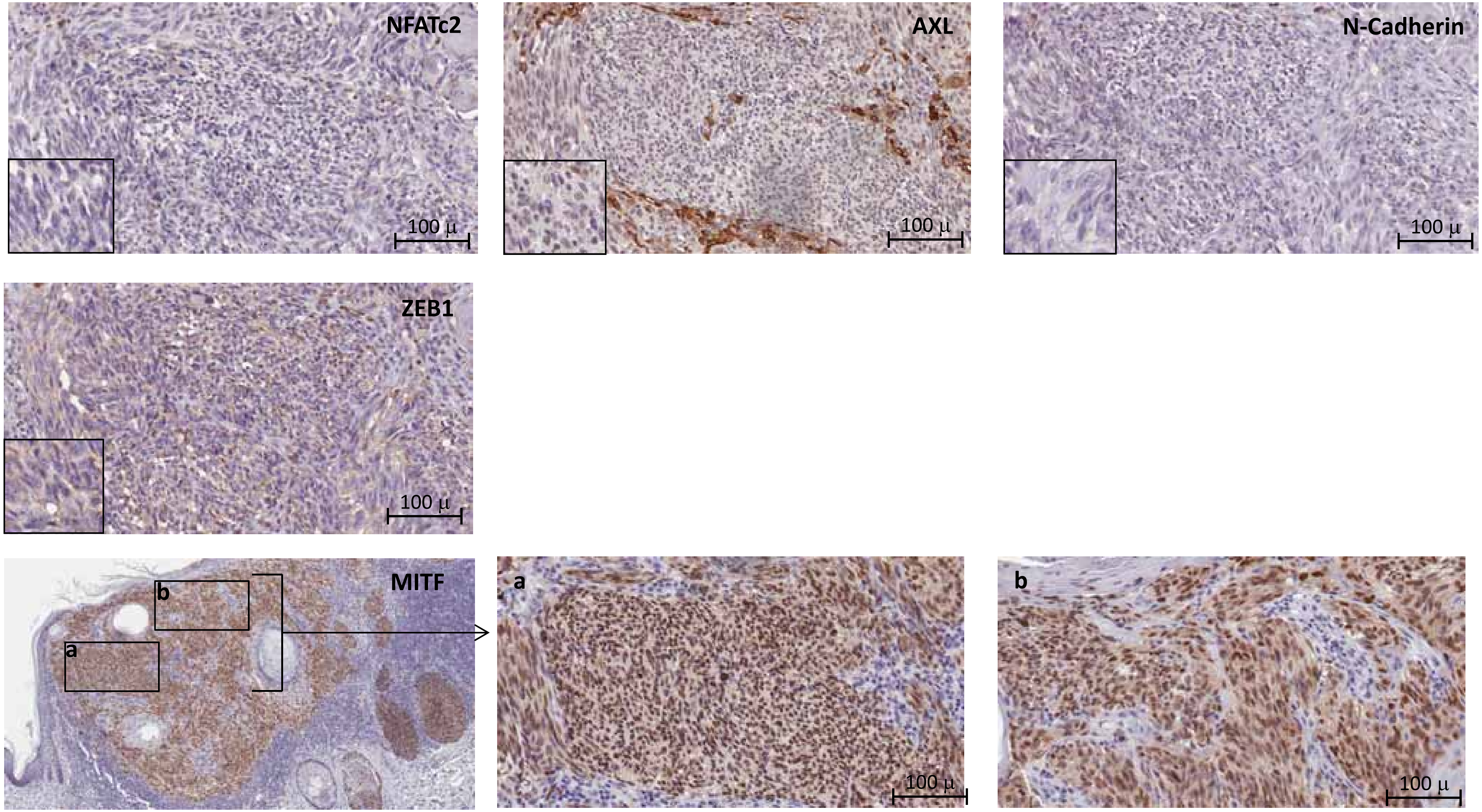

Supplementary Figure S4. Expression of NFATc2, AXL, N-cadherin, ZEB1 and MITF in a melanoma lesion. Characterization by immunohistochemistry of a 1.44 mm thick primary melanoma, Clark level III, with presence of vertical growth phase and lack of ulceration and presence of regression. The tumor does not express NFATc2, AXL, N-cadherin (top panels) and ZEB1 (middle panel), but is strongly positive for MITF (bottom panels). Note that AXL in top panel is not expressed in tumor cells, but is expressed by endothelial cells and by some stromal cells. Insets: higher magnification of a representative area.

Supplementary Figure S5

a

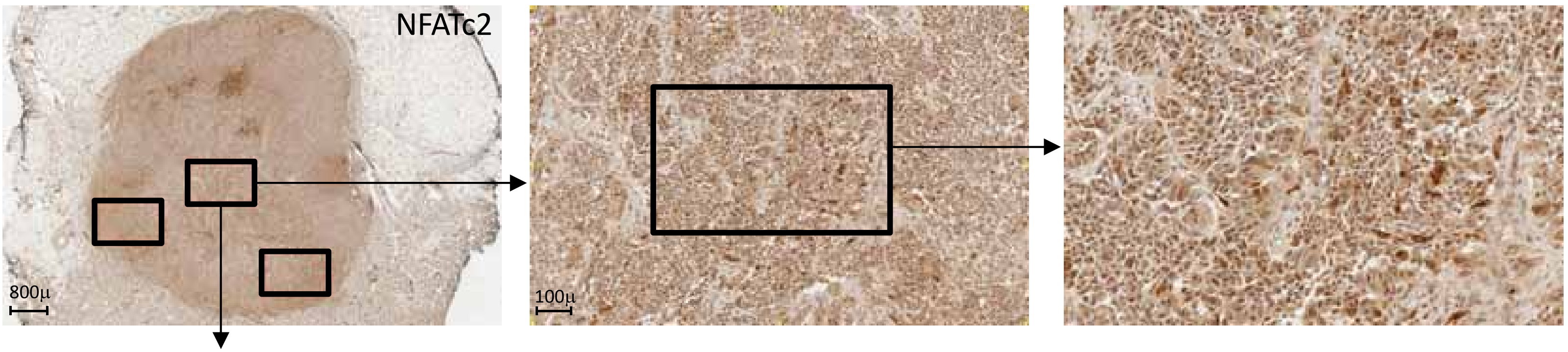

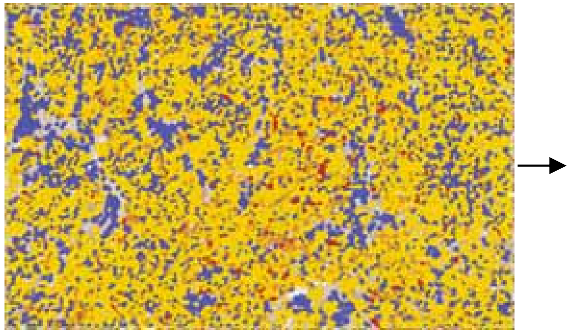

Scoring compartment: nucleus, DAB OD mean

	Color code			
	(dark blue)	(yellow)	(orange)	(red)
Expression class	Neg.	1+	2+	3+
Threshold parameter		0.2	0.4	0.6
cells/mm^2	2,371	4,751	603	141
% of tumor cells	30.1	60.4	7.7	1.8

b

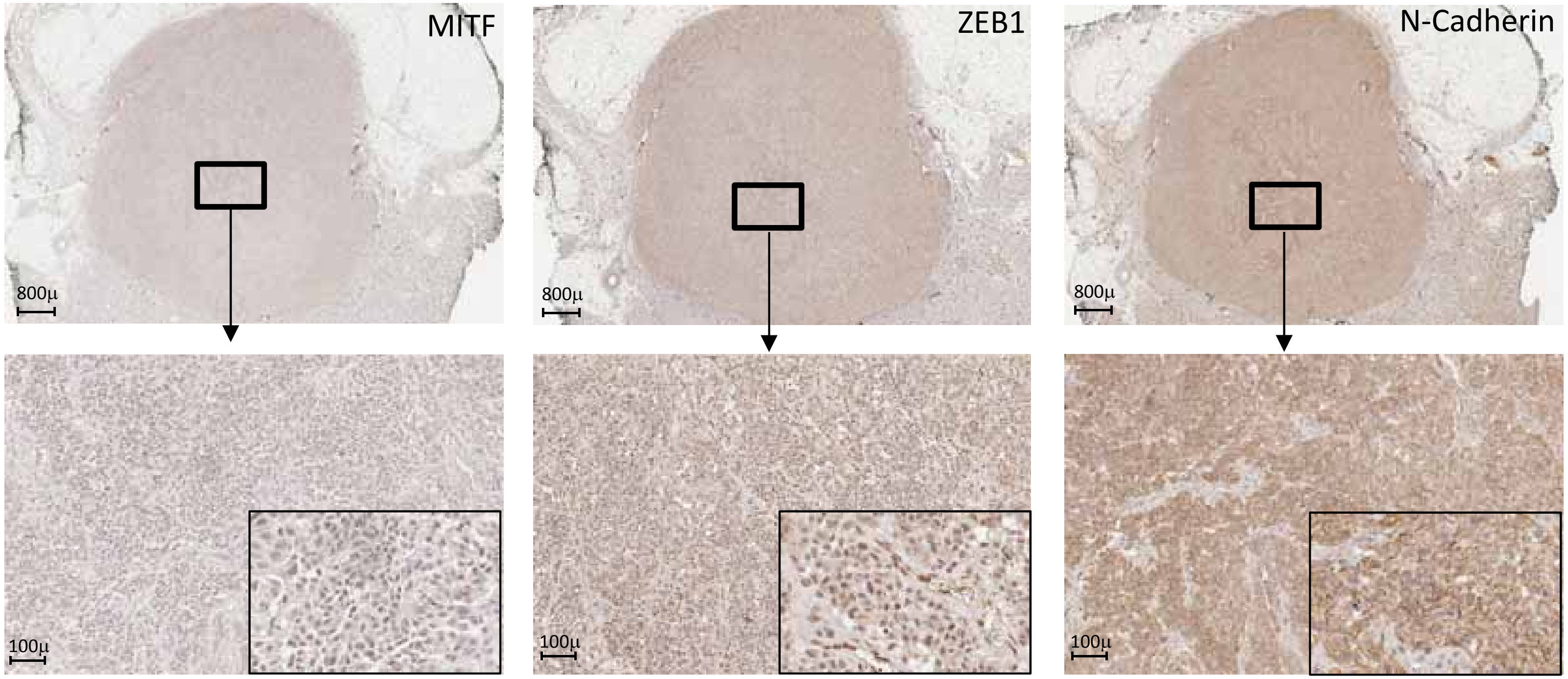

c

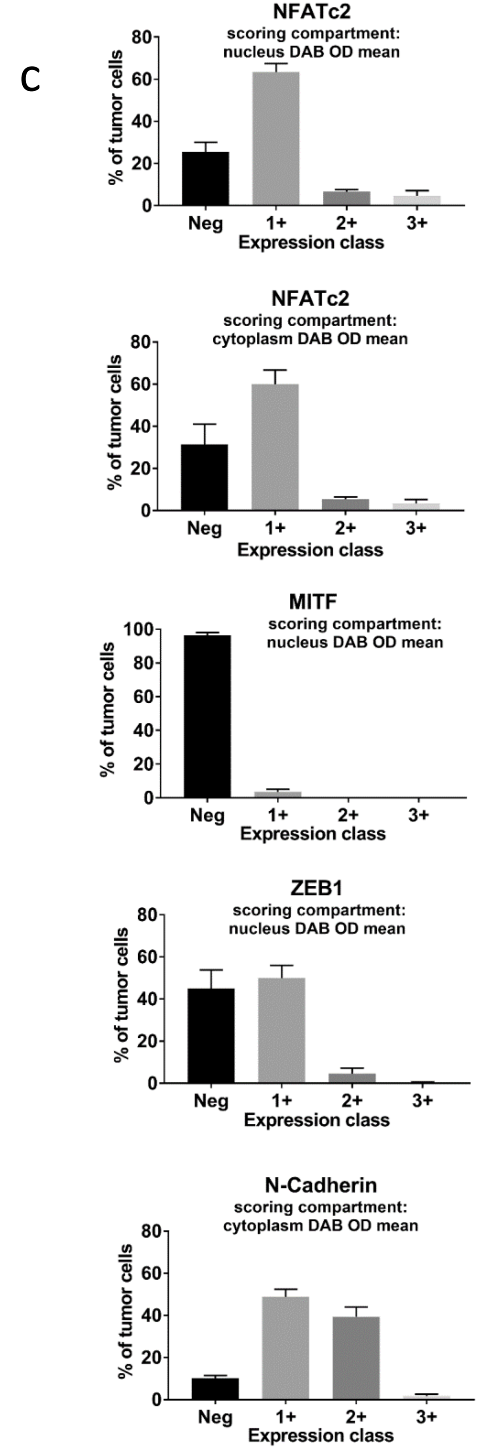

Figure S5 legend after Figure S8

Supplementary Figure S6

a

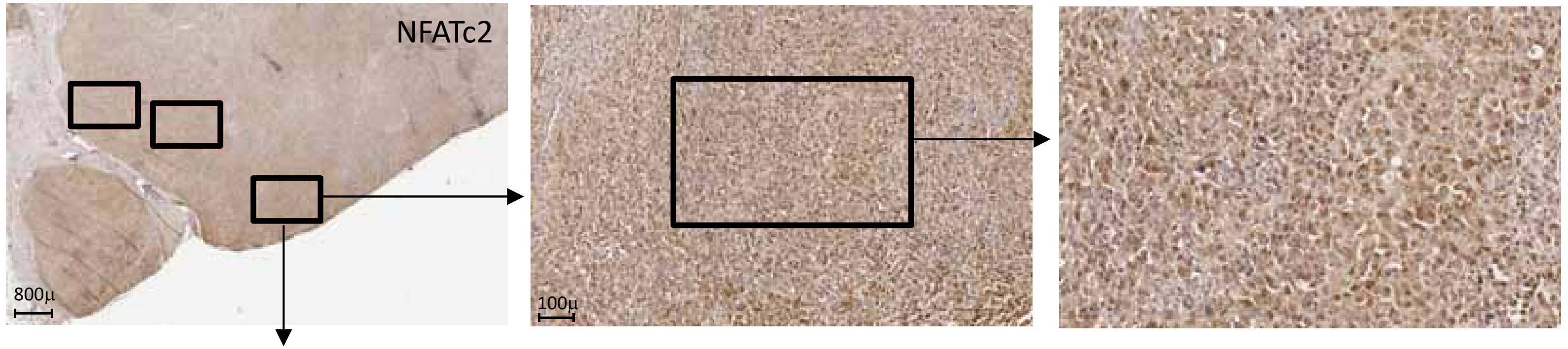

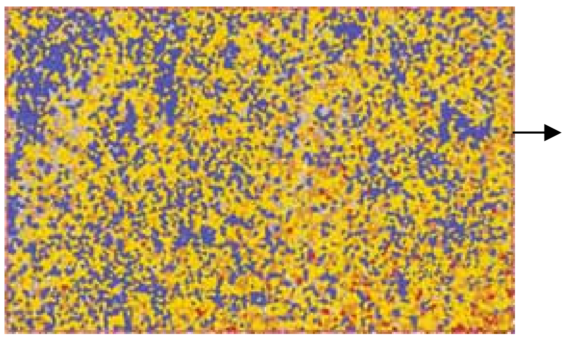

Scoring compartment: nucleus, DAB OD mean

	Color code			
	(dark blue)	(yellow)	(orange)	(red)
Expression class	Neg.	1+	2+	3+
Threshold parameter		0.2	0.4	0.6
cells/mm²	2,871	3,660	724	82
% of tumor cells	39.1	49.9	9.9	1.1

b

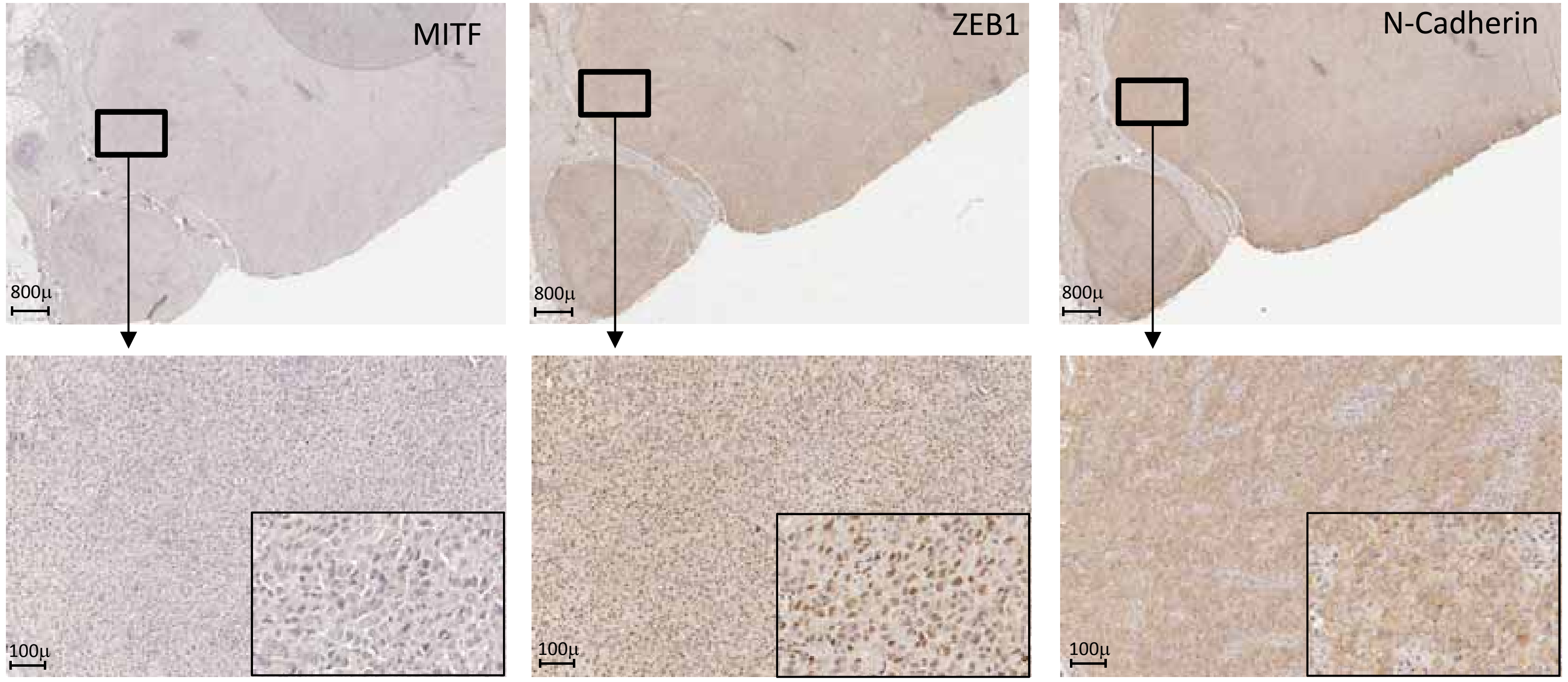

c

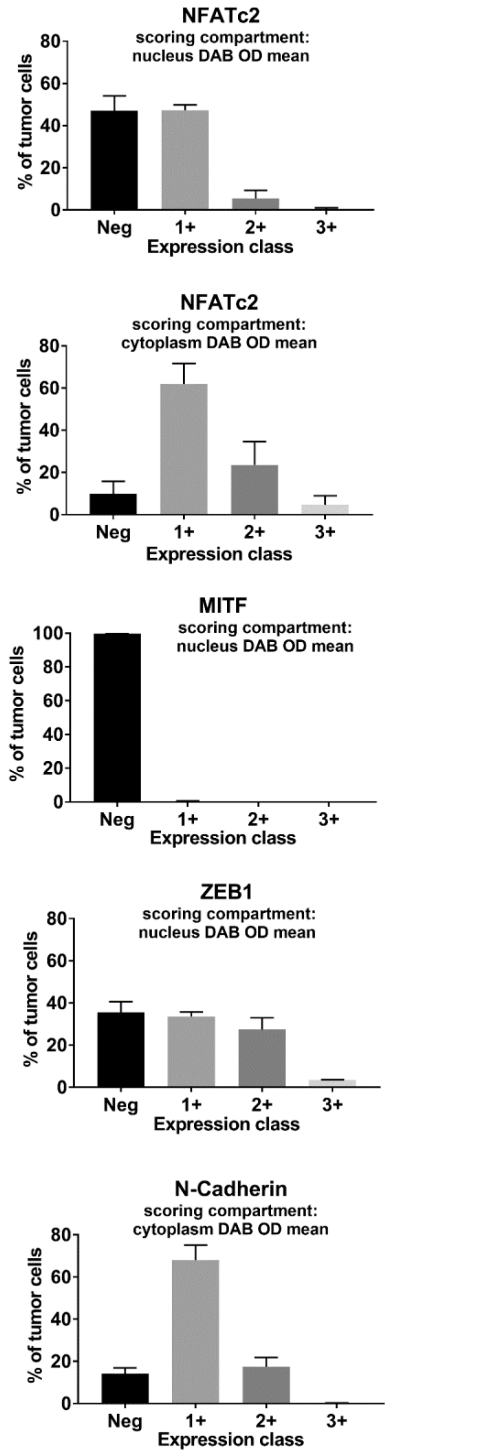

Figure S6 legend after Figure S8

Supplementary Figure S7

a

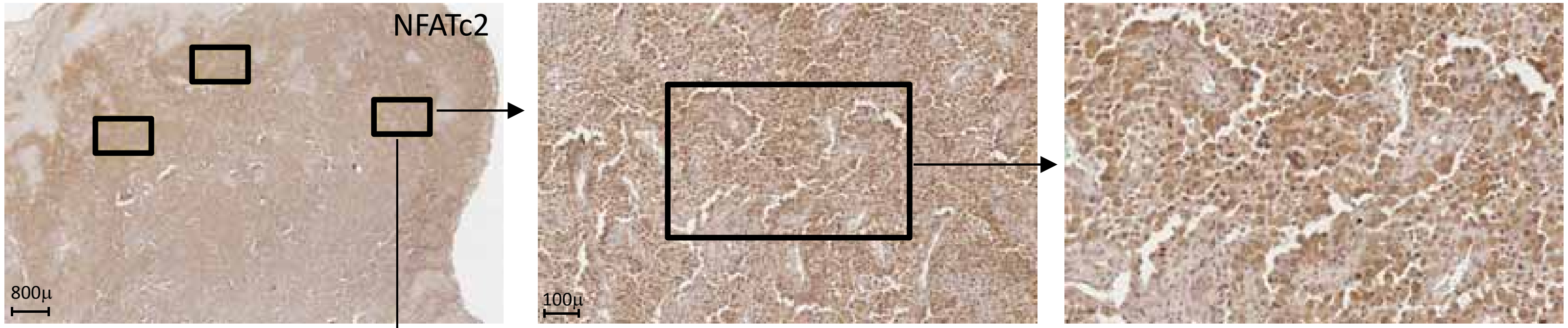

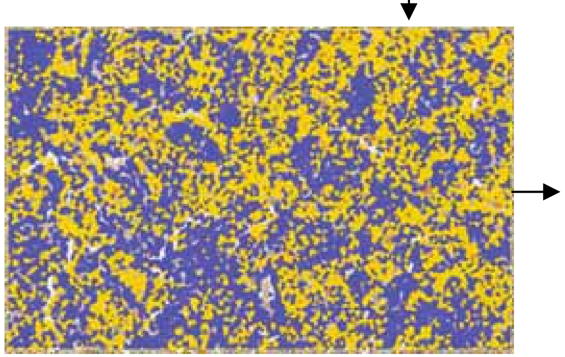

Scoring compartment: nucleus, DAB OD mean

	Color code			
	(dark blue)	(yellow)	(orange)	(red)
Expression class	Neg.	1+	2+	3+
Threshold parameter		0.2	0.4	0.6
cells/mm^2	4,135	2,510	62	1
% of tumor cells	61.6	37.4	0.9	0.01

b

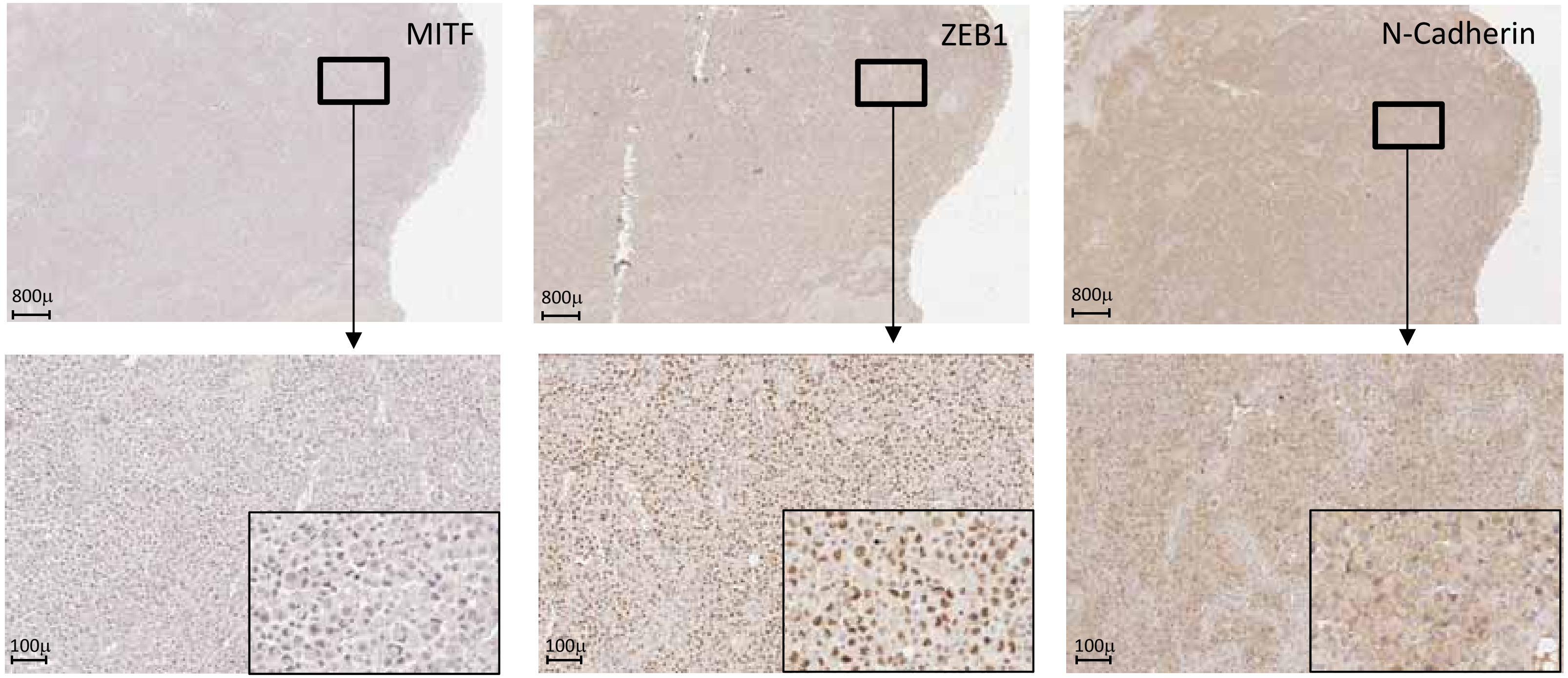

c

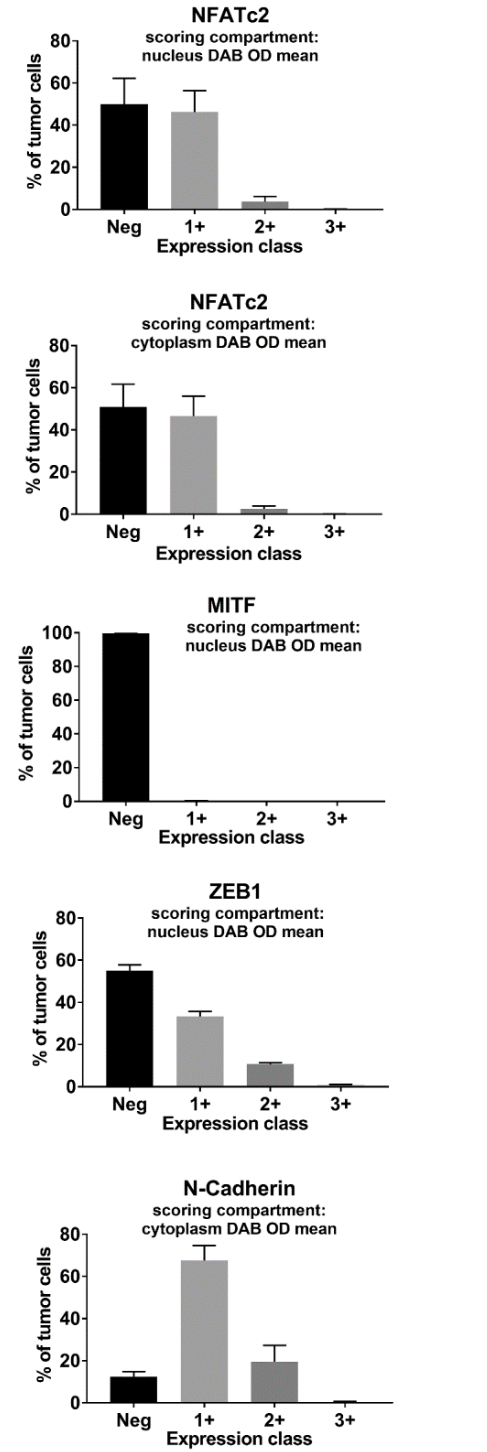

Figure S7 legend after Figure S8

Supplementary Figure S8

a

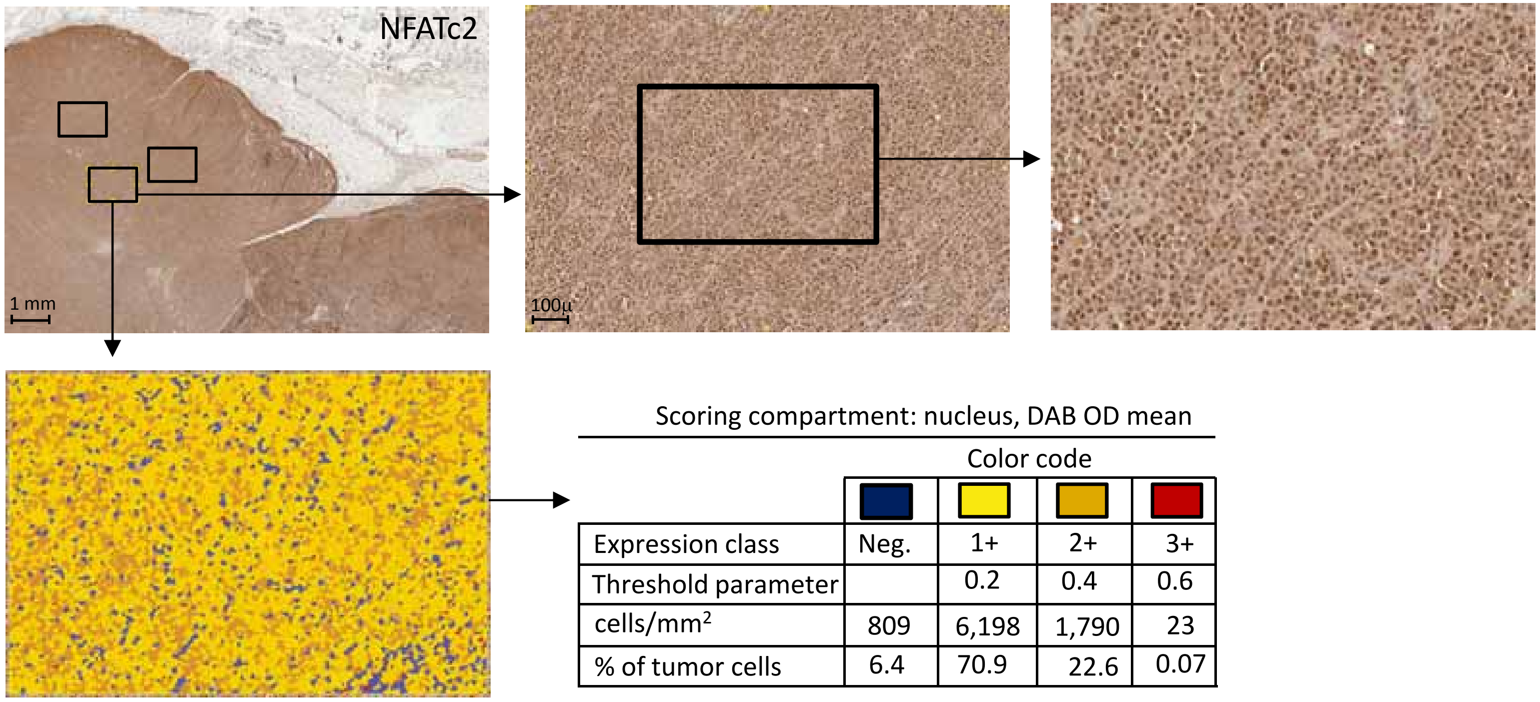

Scoring compartment: nucleus, DAB OD mean

	Color code			
	(dark blue)	(yellow)	(orange)	(red)
Expression class	Neg.	1+	2+	3+
Threshold parameter		0.2	0.4	0.6
cells/mm²	809	6,198	1,790	23
% of tumor cells	6.4	70.9	22.6	0.07

b

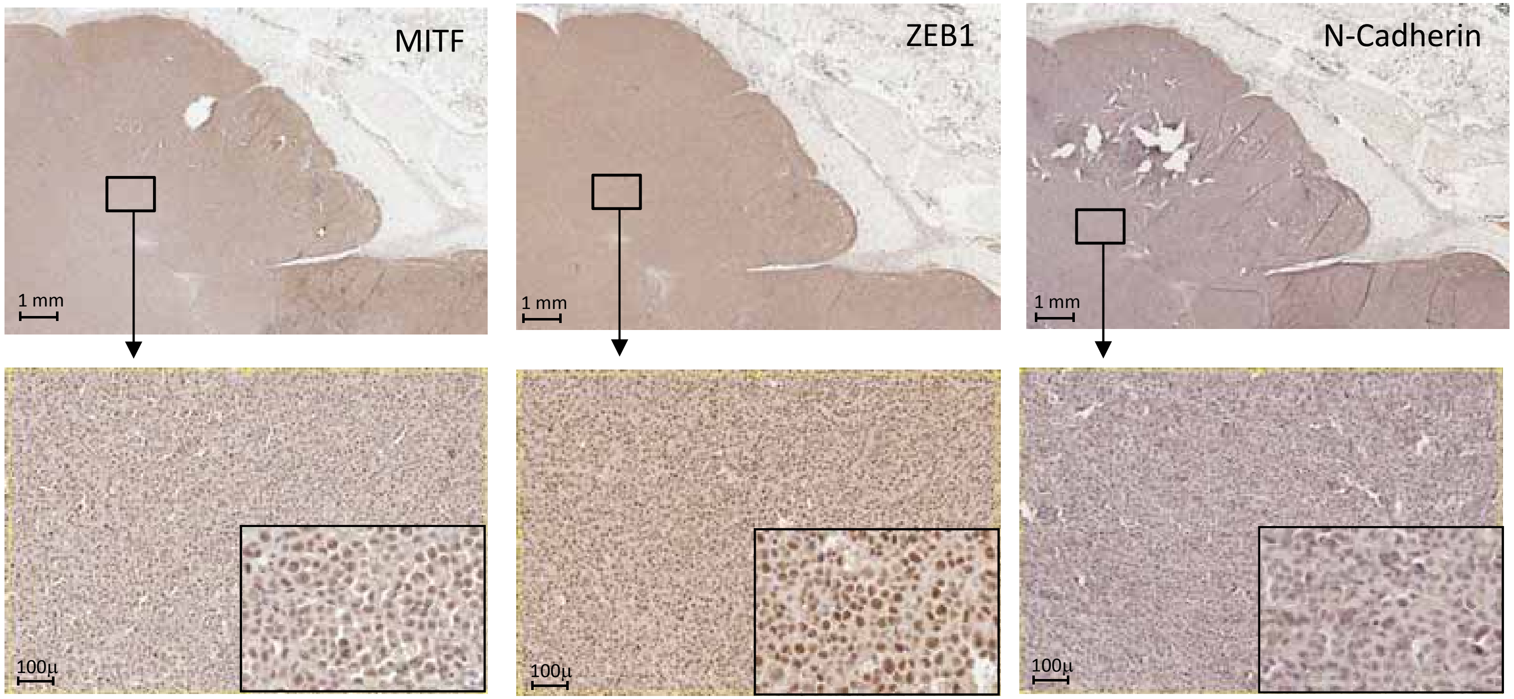

c

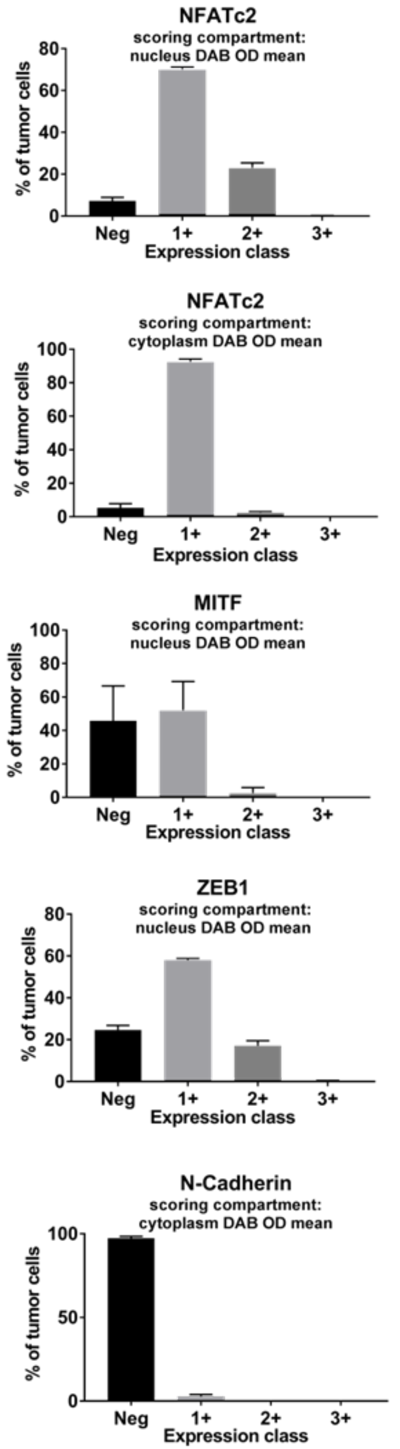

Figure S8 legend, next page

Legends to Figures S5, S6, S7 and S8

Legend to Supplementary Figure S5. **Expression and quantitative analysis by QuPath of NFATc2, MITF, ZEB1 and N-cadherin in metastatic melanoma lesions. a**. Top panels, from left to right, NFATc2 expression in a s.c. melanoma metastasis at three levels of magnification. This lesion has both cytoplasmic and nuclear NFATc2 expression. Bottom panels, identification of cells with nuclear staining for NFATc2 in the 1.5 mm^2 region of interest (ROI) shown in the top panel, by the open source QuPath software. Quantitative analysis was carried out by selecting "nucleus" as scoring compartment and by classifying cells based on three levels of staining intensity (1+, 2+ and 3+, color coded in yellow, orange and red respectively and corresponding to thresholds 0.2, 0.4 and 0.6, as indicated in the table; negative cells were color coded in dark blue). The table shows results for the indicated ROI in terms of cells/mm^2 and % of tumor cells in each of the four expression classes. **b**. Expression of MITF, ZEB1 and N-Cadherin in the same lesion shown in a, at low (top panesl) and high magnification (bottom panels). **c**. Quantitative analysis by QuPath for NFATc2 (in two different scoring compartments, "nucleus" or "cytoplasm"), MITF ("nucleus" scoring compartment), ZEB1 ("nucleus" scoring compartment) and N-cadherin ("cytoplasm" scoring compartment). For each marker, histograms in each graph represent average of values assessed in three rectangular 1.5 mm^2 ROIs.

Legend to Supplementary Figure S6. **Expression and quantitative analysis of NFATc2, MITF, ZEB1 and N-cadherin in s.c. melanoma metastases.**
a. Expression and quantitative analysis of NFATc2 in a s.c. melanoma metastasis, as in the legend to Supplementary Figure S6. **b**. Expression of MITF, ZEB1 and N-Cadherin in the same lesion shown in a, at low (top panesl) and high magnification (bottom panels). **c**. Quantitative analysis by QuPath for NFATc2, MITF, ZEB1 and N-cadherin as described in the legend to Supplementary Figure S6.

Legend to Supplementary Figure S7. **Expression and quantitative analysis of NFATc2, MITF, ZEB1 and N-cadherin in s.c. melanoma metastases**.
a. Expression and quantitative analysis of NFATc2 in a s.c. melanoma metastasis, as in the legend to Supplementary Figure S6. **b**. Expression of MITF, ZEB1 and N-Cadherin in the same lesion shown in a, at low (top panesl) and high magnification (bottom panels). **c**. Quantitative analysis by QuPath for NFATc2, MITF, ZEB1 and N-cadherin as described in the legend to Supplementary Figure S6.

Legend to Supplementary Figure S8. **Expression and quantitative analysis of NFATc2, MITF, ZEB1 and N-cadherin in s.c. melanoma metastases.**
a. Expression and quantitative analysis of NFATc2 in a s.c. melanoma metastasis, as in the legend to Supplementary Figure S6. **b**. Expression of MITF, ZEB1 and N-Cadherin in the same lesion shown in a at low (top panesl) and high magnification (bottom panels). **c**. Quantitative analysis by QuPath for NFATc2, MITF, ZEB1 and N-cadherin as described in the legend to Supplementary Figure S6.

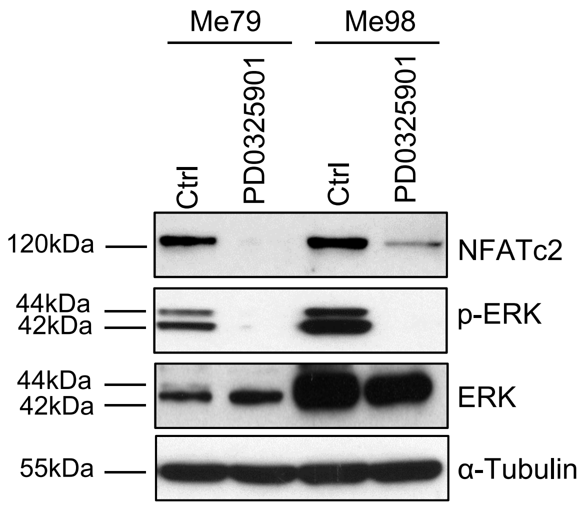

Supplementary Figure S9. **Inhibition of NFATc2 expression by targeting of MEK.** Expression of NFATc2 by western blotting in Me79 and Me98 treated or not with the MEK inhibitor PD0325901 at 100 nM for 48h.

Supplementary Figure S10

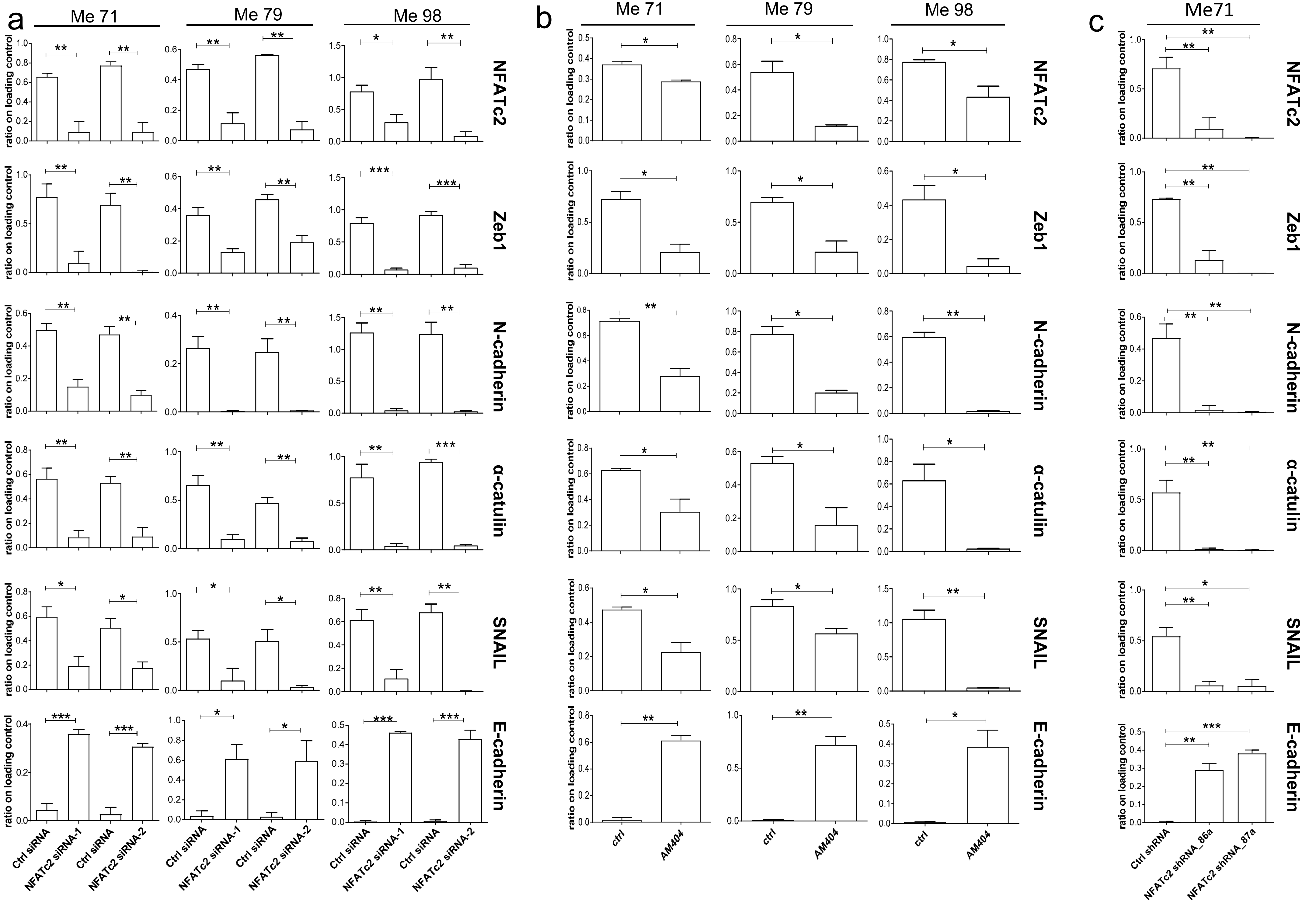

Legend to Supplementary Figure S10. **Densitometric analysis of western blot data related to experiments shown in Fig.2**. **a**, Densitometric analysis, by Quantity One software, of independent replicate experiments (n=2) assessing NFATc2, ZEB1, N-cadherin, α-catulin, SNAIL and E-cadherin expression in Me71, Me 79 and Me 98 cells transfected with NFATc2-siRNA and control-siRNA. **b**, densitometric analysis as in a of the indicated proteins in Me71, Me79 and Me98 treated or not with AM404. **c**, densitometric analysis as in a of the indicated proteins in Me71 transfected with NFATc2 shRNA and control shRNA. Densitometric values are expressed as ratio on the corresponding loading controls after background subtraction. Statistical analysis in a, c by one-way ANOVA and SNK test, in b by paired Student T test. ***: $p<0.001$; **: $p<0.01$; *:$p<0.05$.

Supplementary Figure S11

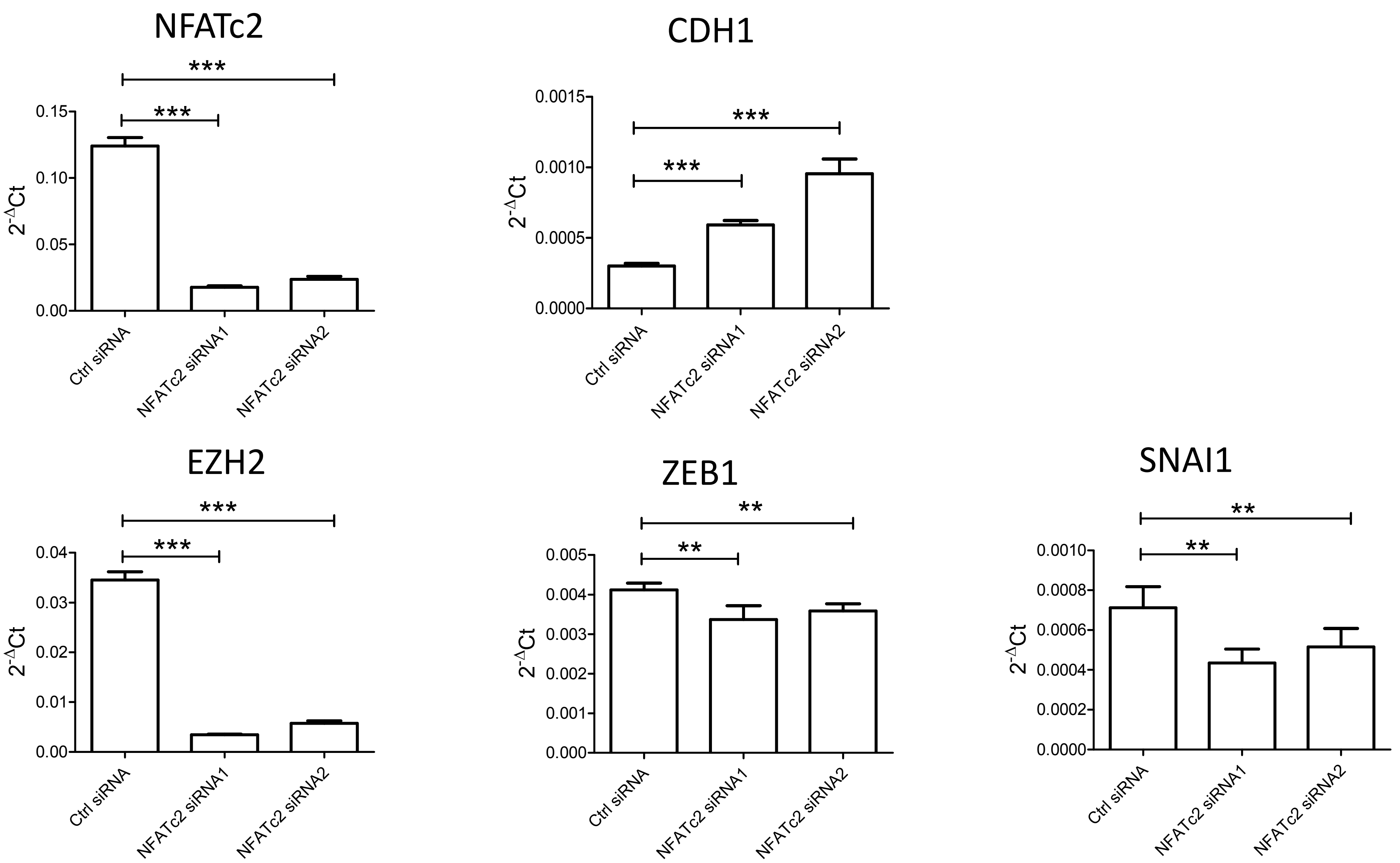

Supplementary Figure S11. Quantitative analysis of gene expression by qPCR in Me79 cells. Me 79 cells were transfected with the indicated NFATc2 siRNA and control siRNA an assessed for expression of the indicated genes by qPCR at 48h. Results expressed a $2^{-\Delta Ct}$. Statistical analysis by one way ANOVA and SNK test. ***: $p<0.001$; **: $p<0.01$; *:$p<0.05$. Data from six independent PCR amplifications.

Supplementary Figure S12

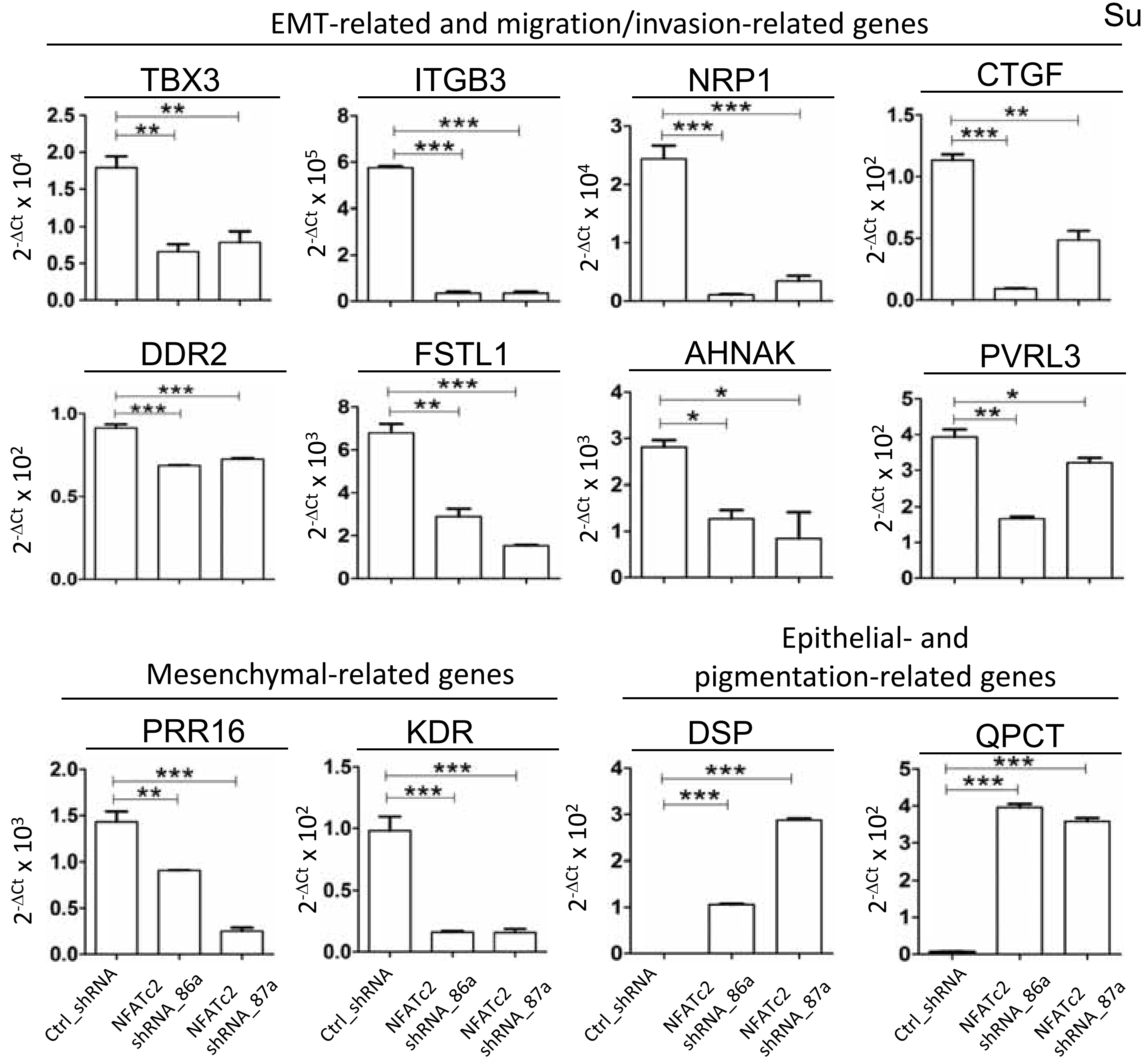

Supplementary Figure S12. **Modulation of EMT-, mesenchymal-, epithelial- and pigmentation-related genes by NFATc2 silencing.** Expression of the indicated genes by qPCR in control (Ctrl_shRNA) and NFATc2 shRNA transfectants (NFATc2 shRNA_86a and NFATc2 shRNA_87a) of Me71. Statistical analysis by ANOVA and SNK post-test. *:p<0.05; **: p<0.01, ***: p<0.0001. Error bars indicate mean ± SD. Data from six independent PCR amplifications.

Supplementary Figure S13

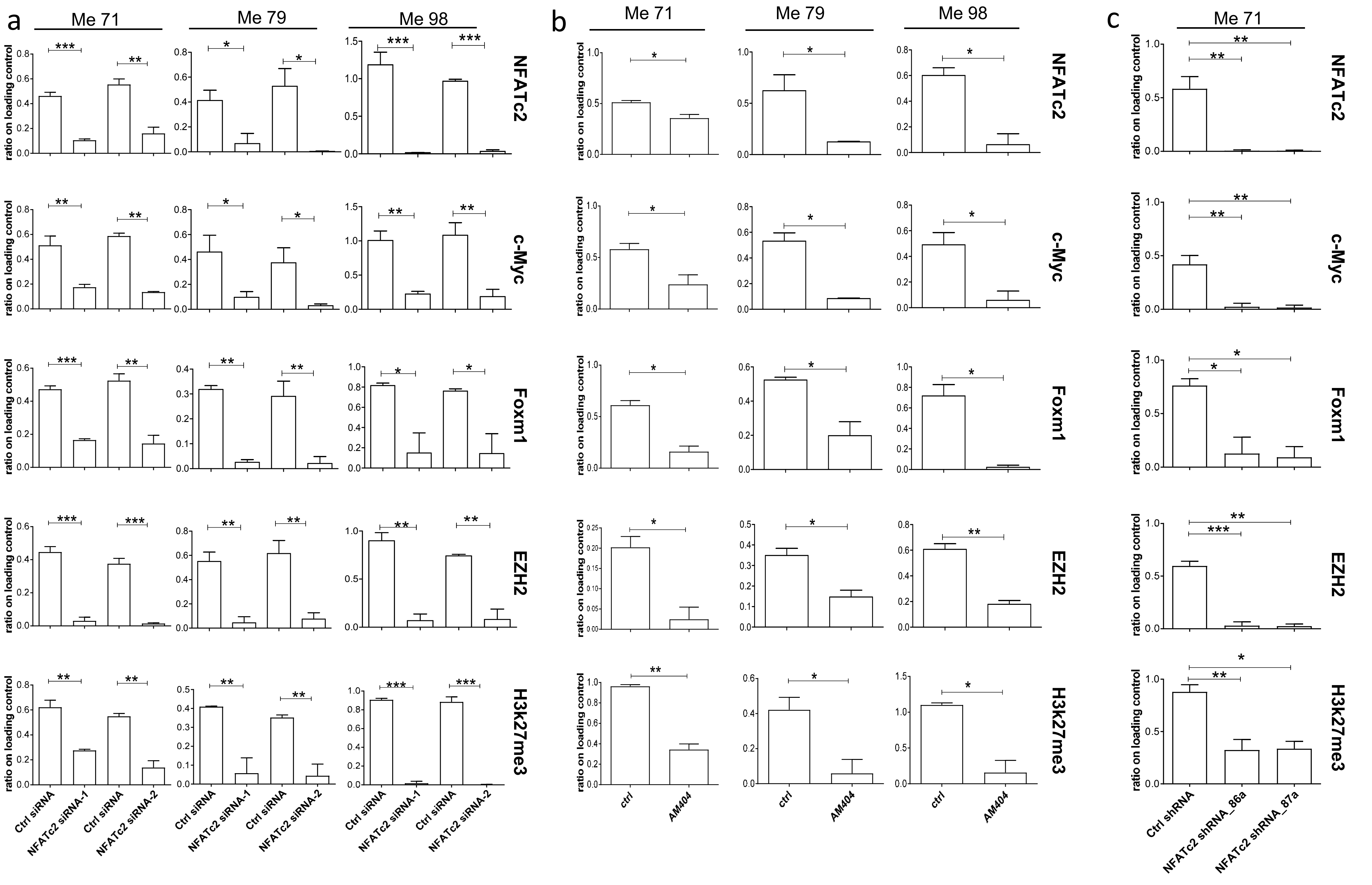

Legend to Supplementary Figure S13. **Densitometric analysis of western blot data related to experiments shown in Fig.3**. **a**, Densitometric analysis, by Quantity One software, of independent replicate experiments (n=2) assessing NFATc2, c-Myc, FOXM1, EZH2, H3K27me3 expression in Me71, Me 79 and Me 98 cells transfected with NFATc2 siRNA and control siRNA. **b**, densitometric analysis as in a of the indicated proteins in Me71, Me79 and Me98 treated or not with AM404. **c**, densitometric analysis as in **a** of the indicated proteins in Me71 transfected with NFATc2 shRNA and control shRNA. Densitometric values are expressed as ratio on the corresponding loading controls after background subtraction. Statistical analysis in a, c by one-way ANOVA and SNK test, in b by paired Student T test. ***: $p<0.001$; **: $p<0.01$; *:$p<0.05$.

Supplementary Figure S14

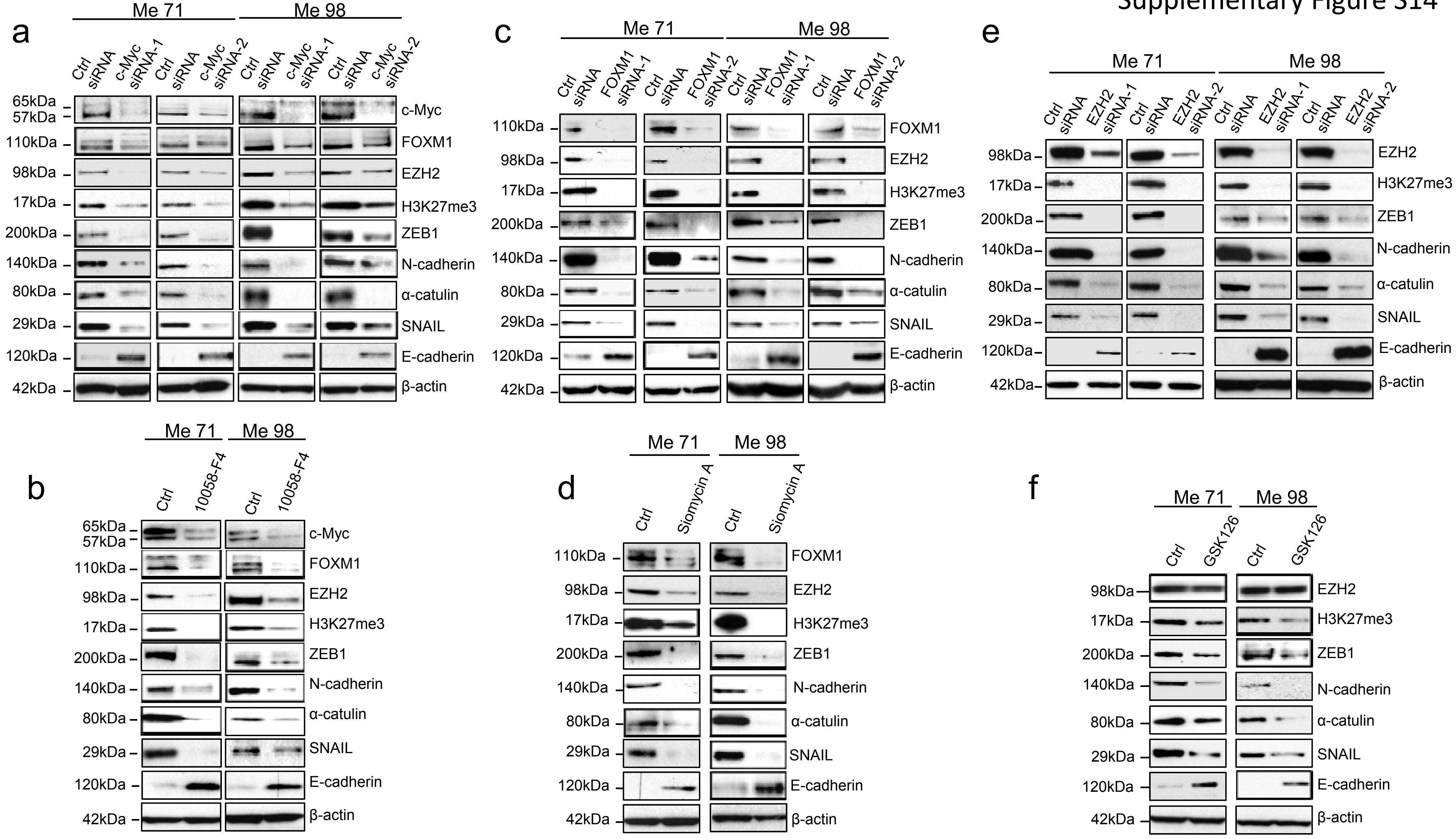

Supplementary Figure S14. **Regulation of FOXM1 and EZH2 by c-Myc, of EZH2 by FOXM1 and downregulation of EMT-related markers by targeting of c-Myc, FOXM1 and EZH2 in melanoma cell lines Me71 and Me98. a,b** Expression by western blotting of c-Myc, FOXM1, EZH2, H3K27me3 and of different EMT-related markers in melanoma cell lines Me71 and Me98 at 72h (a) after transfection with two different c-Myc-specific Sthealth siRNA (c-Myc siRNA-1 and siRNA-2) or with control siRNA (ctrl siRNA), or (b) at 48h after treatment with c-Myc inhibitor 10058-F4 at 20μM. **c,d,e,f** Expression by western blotting of the indicated proteins after transfection of Me71 and Me 98 at 72h (c, e) with two different FOXM1-specific (c) or two different EZH2-specific (e) siRNA or with control siRNA, or at 48 h (d,f) after treatment with FOXM-1 inhibitor Siomycin A at 2 μM (d) or with EZH2 inhibitor GSK126 at 5 μM (f).

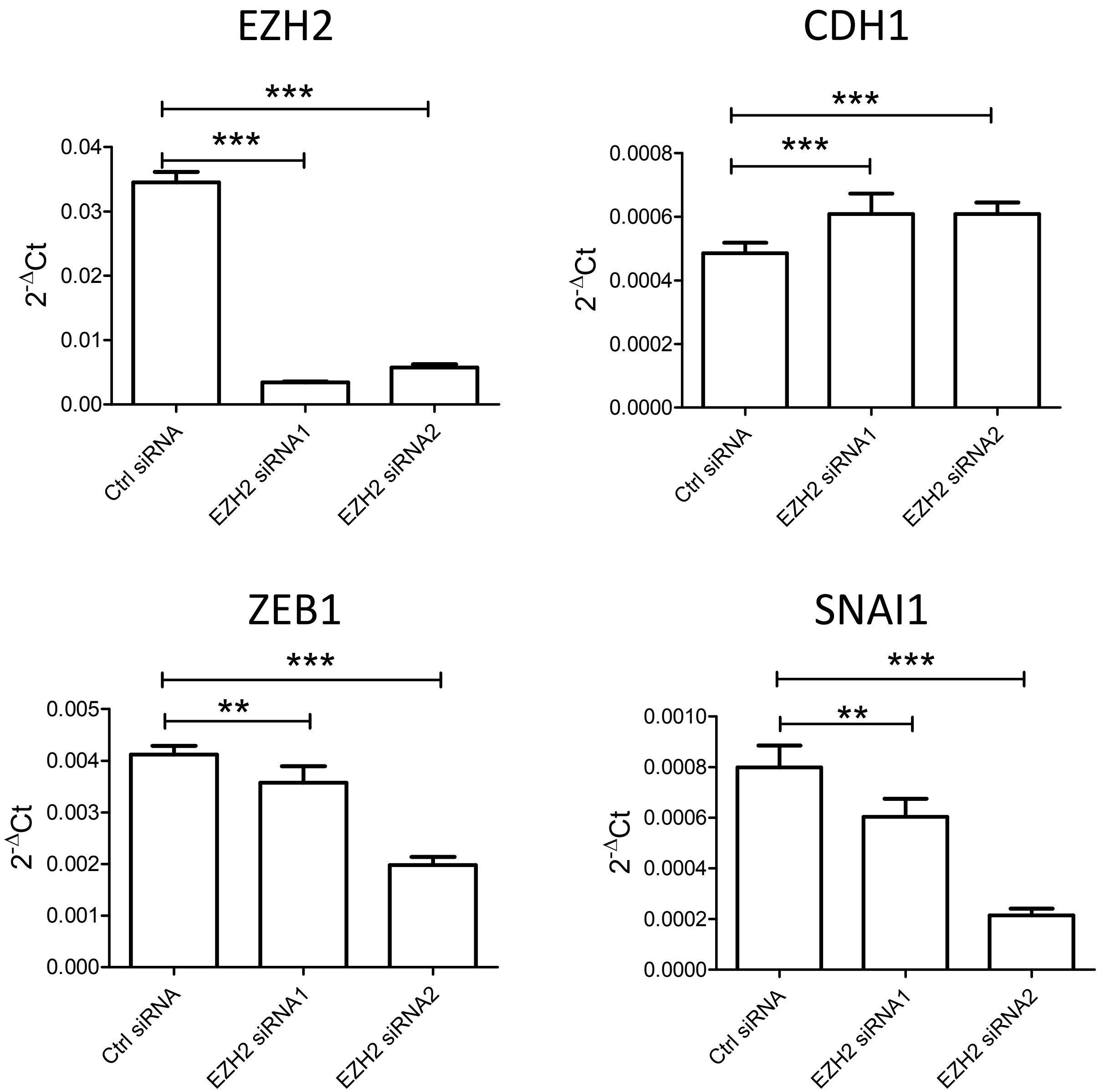

Supplementary Figure S15. Quantitative analysis of gene expression by qPCR in Me79 cells. Me 79 cells were transfected with the indicated EZH2 siRNA and control siRNA an assessed for expression of the indicated genes by qPCR at 48h. Results expressed a $2^{-\Delta Ct}$. Statistical analysis by one way ANOVA and SNK test. ***: p<0.001; **: p<0.01; *:p<0.05. Data from six independent PCR amplifications.

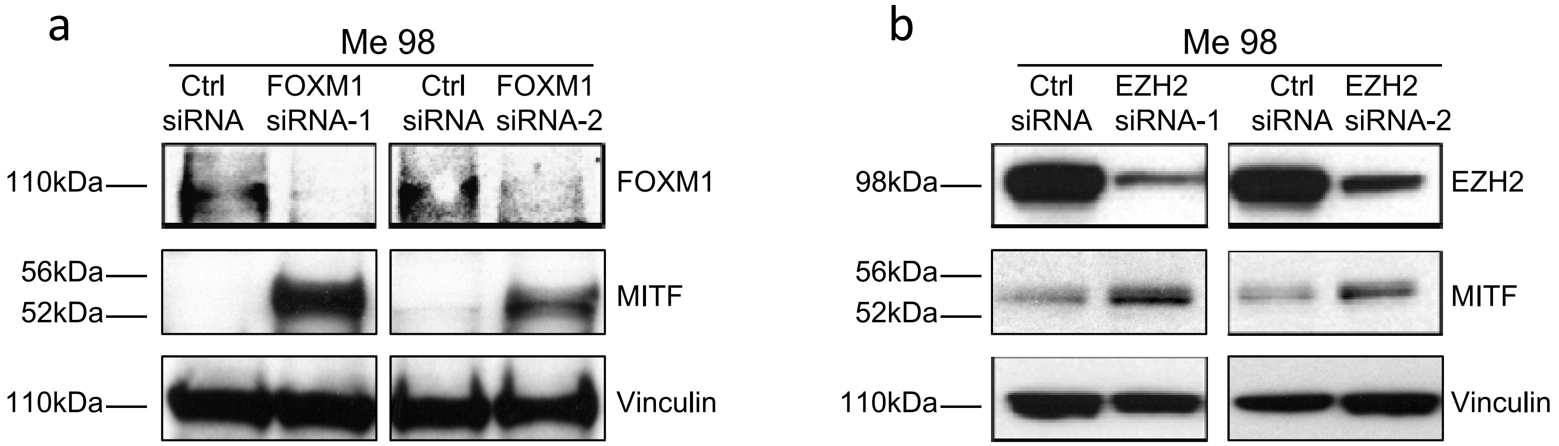

Supplementary Figure S16. Targeting of FOXM1 and of EZH2 promotes MITF upregulation. a, b Expression by western blot analysis of FOXM1 and MITF (a) and of EZH2 and MITF (b) at 96h after transfection of Me98 cells with (a) two different FOXM1-specific stealth small interfering RNA (FOXM1 siRNA-1, FOXM1 siRNA-2) and related control siRNA (ctrl siRNA) or (b) two different EZH2-specific stealth small interfering RNA (EZH2 siRNA-1, EZH2 siRNA-2) and related control siRNA (ctrl siRNA).

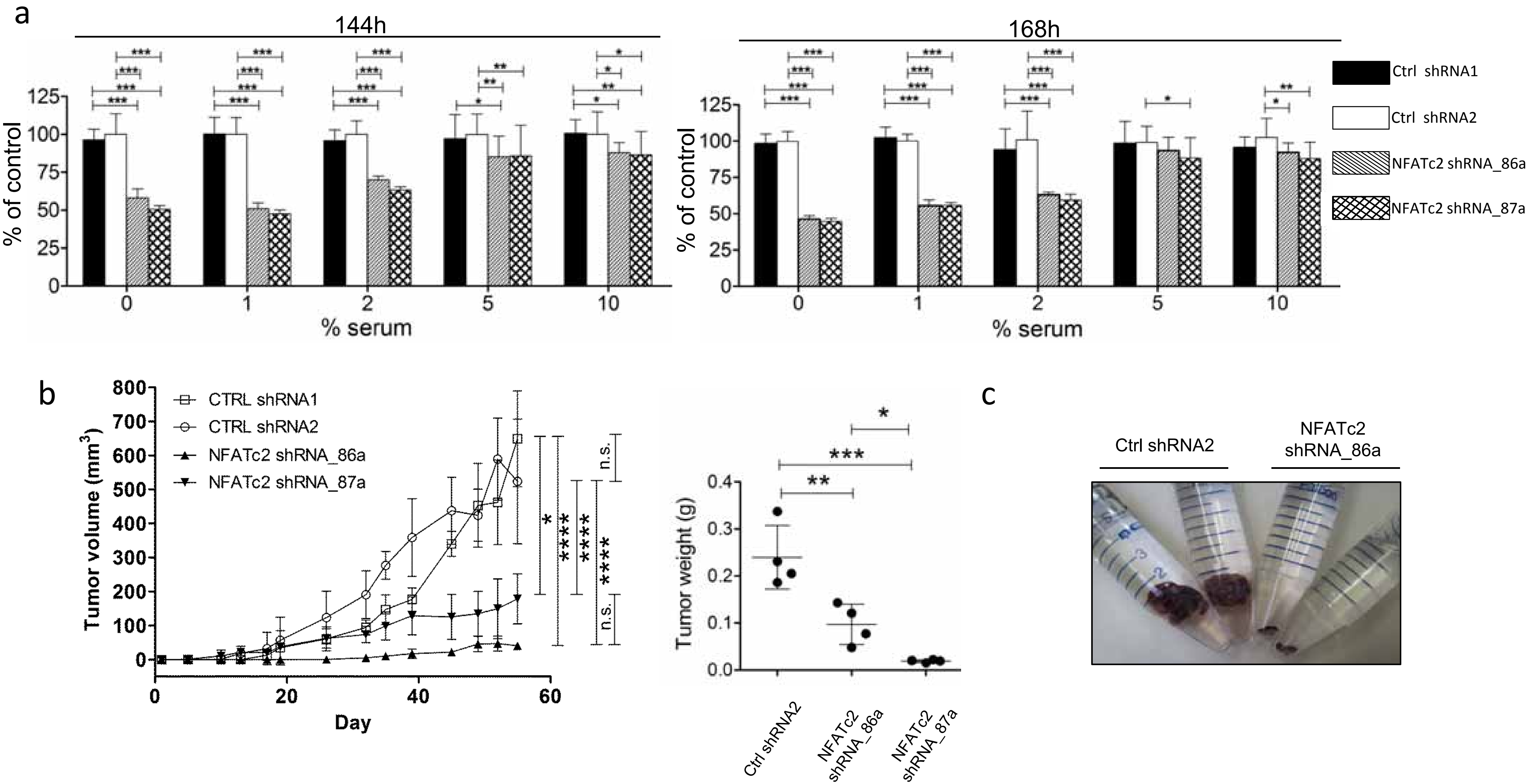

Supplementary Figure S17. Inhibition of in-vitro and in-vivo melanoma growth by NFATc2 silencing. a In-vitro growth, by MTT assay, at 144 (left graph) or 168h (right graph), of NFATc2 shRNA transfectants (NFATc2 shRNA_86a: hatched bars; NFATc2 shRNA_87a: cross-hatched bars) and two different control transfectants (black bars and empty bars) from Me71, in presence of 0 to 10% FCS (serum). **b** in-vivo growth after s.c. injection in nude mice of two NFATc2 shRNA transfectants and two different control transfectants. Left graft: tumor volume; right graph: tumor weight. **c** Images of tumor nodules removed at day +55 from two animals injected s.c. with NFATc2 control transfectants (Ctrl shRNA2) and from two animals injected with NFATc2 shRNA transfectants (NFATc2 shRNA_86a). Statistical analysis in (a) by two way ANOVA and Bonferroni post-test, in (b), left graph, by mixed models ANOVA followed by Bonferroni post-test and, right graph, by one way ANOVA and SNK post-test,***:*p*<0.001; **:*p*<0.01; *:*p*<0.05. Error bars indicate mean ± SD. Data in (a) from two independent experiments (each with six replicates). Data in b: four animals/group.

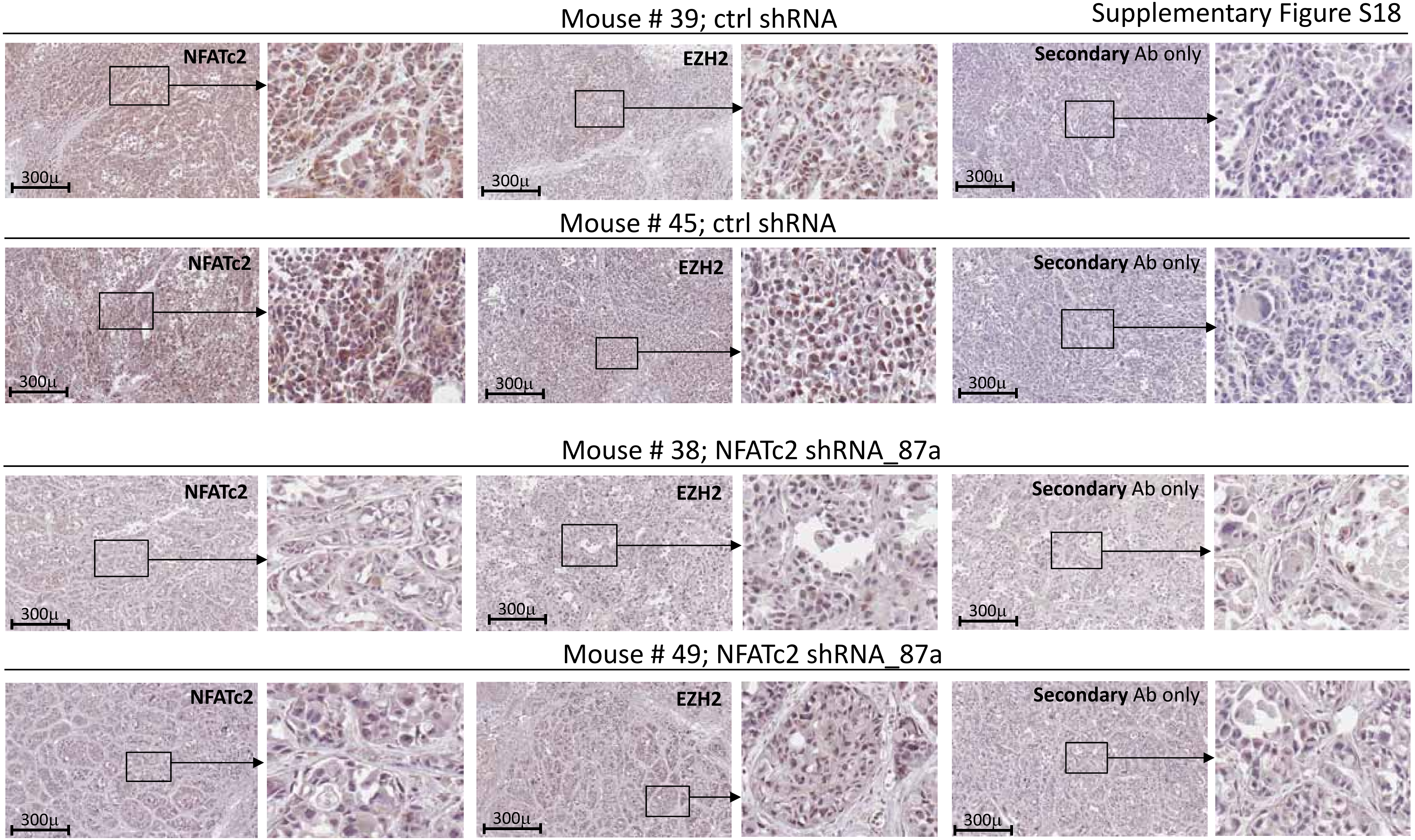

Supplementary Figure S18. NFATc2 and EZH2 phenotype of neoplastic nodules removed from animals injected with control shRNA or NFATc2 shRNA melanoma transfectants. Expression of NFATc2 (left panels) and EZH2 (middle panels) in tumor nodules removed at day +55 from two animals (Mouse #39 and #45) injected s.c. with NFATc2 control transfectants (Ctrl shRNA) and from two animals (Mouse #38 and #49) injected with NFATc2 shRNA transfectants (NFATc2 shRNA_87a) from Me71. Right hand panels: sections stained with secondary Ab only. Insets: higher magnification of a representative area.

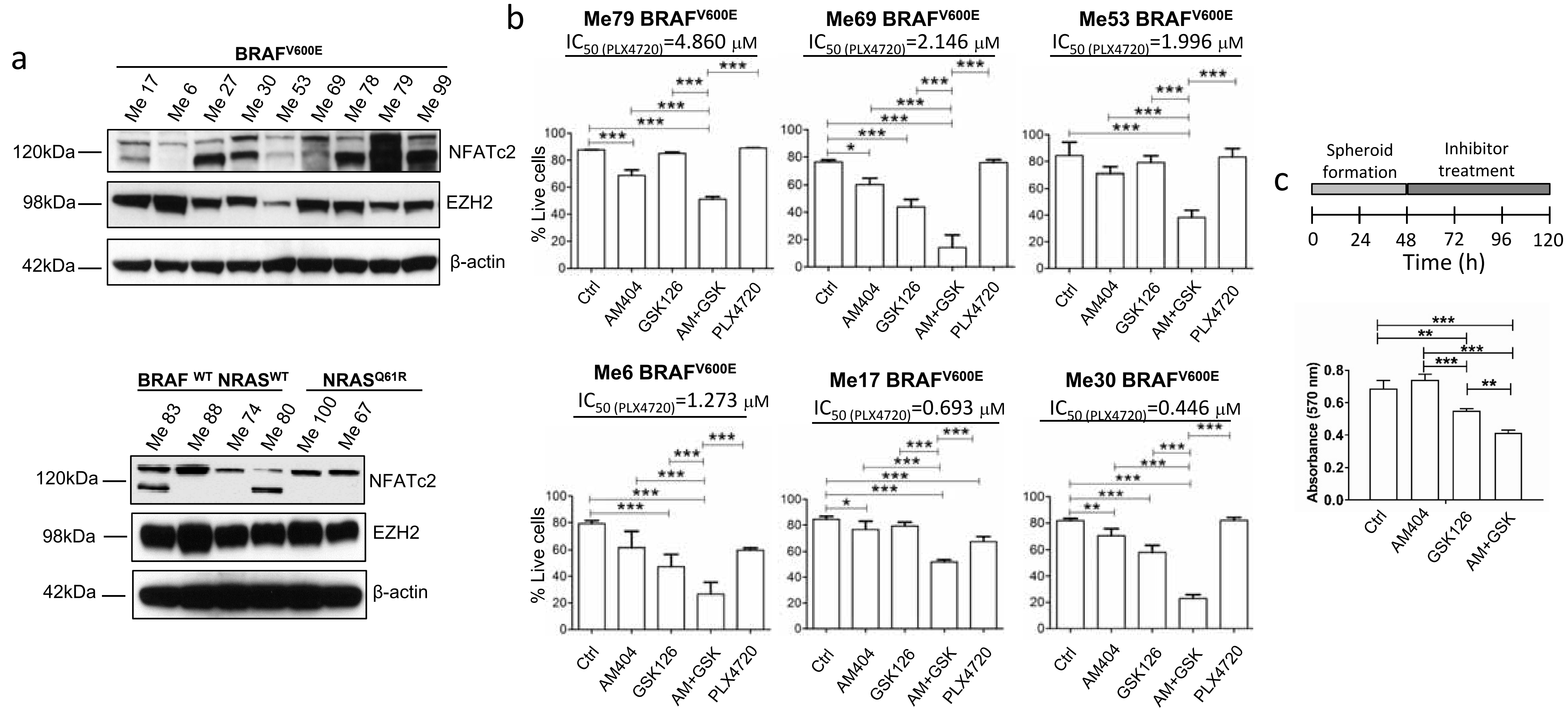

Supplementary Figure S19. **Expression of NFATc2 and of EZH2 in melanoma cell lines belonging to distinct mutational subsets and promotion of apoptosis in BRAF inhibitor-resistant $BRAF^{V600E}$-mutant melanoma cell lines by co-targeting of NFATc2 and EZH2**. **a** Expression of NFATc2 and of EZH2, by western blot analysis, in melanoma cell lines used for apoptosis experiments shown in panel b and in Fig. 6a,b. **b** Apoptosis, by Annexin-V/PI assay at 48h, in BRAF inhibitor-resistant $BRAF^{V600E}$-mutant melanoma cell lines treated or not with NFATc2 inhibitor AM404, or with EZH2 inhibitor GSK126, or with the combination of AM404 and GSK126, or with PLX4720 as in Fig. 6b. IC_{50} for the BRAF inhibitor PLX4720 of all cell lines, as published in ref. 35 is shown above each panel. **c,** timing of spheroid formation and inhibitor treatment (top) to test the anti-tumor activity of AM404 (15 μM), GSK126 (10 μM) and their combination (bottom) on Me 88 cells tested in a 3D spheroid tumor model. Statistical analysis in b, c by ANOVA and SNK post-test. *:p<0.05; **:p<0.001, ***:p<0.001. Error bars indicate mean ± SD. Data from three independent experiments.

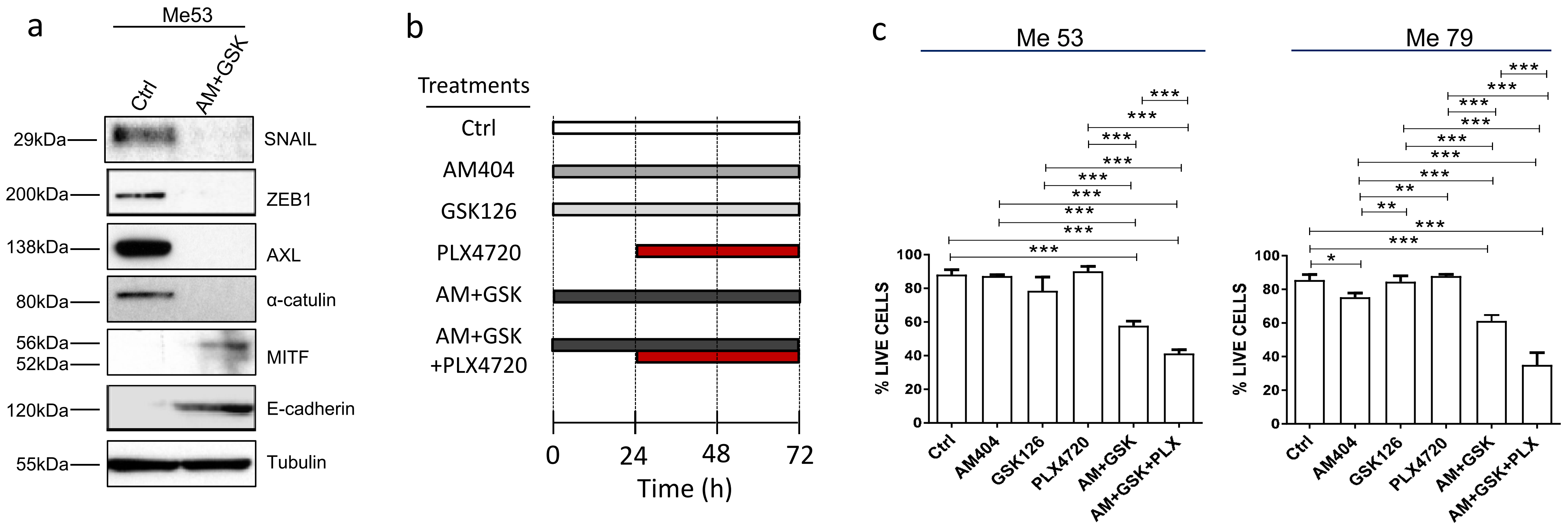

Supplementary Figure S20. Co-targeting of NFATc2 and EZH2 improves response to BRAF inhibition and reverses the EMT-lik melanoma profile. a, expression of SNAIL, ZEB1, AXL, α-catulin, MITF, and E-cadherin in Me53 cells treated or not O/N with the association of AM404 (15 μM) and GSK126 (10 μM). **b**, timing of combinatorial treatments related to data shown in panel c. **c**, two NFATc2^{+}, BRAFi-resistant melanoma cell lines were treated with AM404 (15 μM), GSK126 (10 μM) or the combination of these two inhibitors. After 24 h, PLX4720 (1μM) was added to untreated cells and to cells pre-treated with the AM+GSK combination. Apoptosis was then evaluated after a further 48h of culture. Results expressed as % live cells. Statistical analysis by one way ANOVA and SNK test. ***:p<0.001; **:p<0.01; *:p<0.05.
