# Supplementary material for: An actionable axis linking NFATc2 to EZH2 controls the EMT-like program of melanoma cells
Source: Oncogene. 2019 Feb 1;38(22):4384–96. doi: 10.1038/s41388-019-0729-2 (PMC6756060; doi:10.1038/s41388-019-0729-2)
Supplement: Supplementary file 2 — Supplementary Table [file 41388_2019_729_MOESM2_ESM.pdf]

Supplementary Table S1.

Hierarchical clustering of correlation values (Spearman score) of the 312 ZEB1-correlated genes (rows, selected for r≥0.5 with ZEB1) with SNAI1, NFATc2, CDH2, AXL, MITF and CDH1 (columns). Positive scores are highlighted in red, negative scores are highlighted in blue.
Data were retrieved from the TCGA melanoma dataset at www.cbioportal.org.

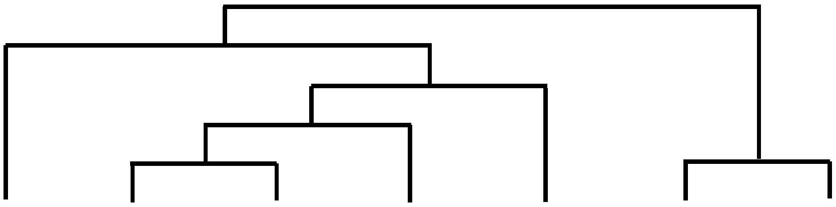

Gene Symbol	SNAI1	NFATC2	ZEB1	CDH2	AXL	MITF	CDH1
GPC6	0.36	0.26	0.64	0.42	0.42	-0.35	-0.31
CFI	0.35	0.27	0.6	0.32	0.36	-0.34	-0.29
B3GNT5	0.41	0.26	0.62	0.34	0.35	-0.39	-0.37
LRRC17	0.38	0.2	0.59	0.3	0.32	-0.3	-0.27
MIR100HG	0.31	0.27	0.69	0.39	0.39	-0.36	-0.32
PDGFC	0.25	0.27	0.68	0.41	0.41	-0.4	-0.33
ADAMTS6	0.26	0.26	0.65	0.37	0.44	-0.32	-0.33
SYT1	0.22	0.22	0.51	0.3	0.37	-0.34	-0.3
IFT57	0.28	0.21	0.54	0.34	0.26	-0.26	-0.27
NFYB	0.2	0.18	0.5	0.3	0.25	-0.21	-0.25
ERRFI1	0.37	0.24	0.61	0.44	0.37	-0.42	-0.47
PCDH9	0.28	0.28	0.51	0.45	0.23	-0.34	-0.33
CHN1	0.28	0.18	0.52	0.32	0.36	-0.42	-0.25
GEM	0.35	0.19	0.53	0.32	0.45	-0.4	-0.32
TMC7	0.38	0.16	0.5	0.26	0.45	-0.43	-0.27
LAMA4	0.32	0.46	0.57	0.45	0.28	-0.3	-0.23
ELK3	0.31	0.53	0.6	0.39	0.36	-0.21	-0.24
AHR	0.24	0.5	0.53	0.39	0.26	-0.24	-0.27
EPHA3	0.38	0.45	0.54	0.37	0.39	-0.29	-0.17
THRB	0.28	0.31	0.64	0.31	0.32	-0.42	-0.21
FGF2	0.33	0.38	0.6	0.3	0.29	-0.34	-0.19
KCTD12	0.32	0.44	0.68	0.41	0.42	-0.25	-0.23
KCNT2	0.29	0.35	0.61	0.32	0.34	-0.26	-0.23
RIN2	0.32	0.39	0.65	0.33	0.42	-0.36	-0.3
LIFR	0.31	0.4	0.64	0.31	0.36	-0.31	-0.32
TMEM47	0.31	0.38	0.65	0.45	0.43	-0.37	-0.36
CDK14	0.29	0.37	0.64	0.4	0.32	-0.32	-0.33
CCDC102B	0.26	0.31	0.56	0.33	0.33	-0.27	-0.27
EDIL3	0.33	0.37	0.61	0.41	0.37	-0.31	-0.26
PDE1C	0.32	0.33	0.54	0.35	0.37	-0.28	-0.22
GDNF	0.34	0.41	0.54	0.4	0.35	-0.34	-0.26
ABCA6	0.34	0.45	0.58	0.28	0.37	-0.36	-0.28
ITGA1	0.41	0.48	0.63	0.36	0.36	-0.35	-0.34
TMEM87B	0.36	0.37	0.54	0.28	0.35	-0.3	-0.27
DOCK4	0.36	0.54	0.67	0.41	0.38	-0.33	-0.29
PELI1	0.26	0.43	0.53	0.32	0.27	-0.31	-0.26
STAMBPL1	0.25	0.43	0.54	0.29	0.31	-0.28	-0.31
ATP2B1	0.38	0.45	0.67	0.42	0.24	-0.32	-0.32
MMP16	0.4	0.42	0.61	0.38	0.28	-0.3	-0.32
KDELC2	0.29	0.33	0.5	0.31	0.26	-0.25	-0.3
VASH2	0.29	0.31	0.5	0.29	0.26	-0.26	-0.24
ANGPT1	0.31	0.27	0.51	0.29	0.2	-0.31	-0.25

Spearman r values
1
0.9
0.8
0.7
0.6
0.5
0.4
0.3
0.2
0.1
0
-0.1
-0.2
-0.3
-0.4
-0.5
-0.6

GRIA2	0.29	0.28	0.5	0.28	0.19	-0.29	-0.26
NEGR1	0.32	0.33	0.5	0.38	0.23	-0.34	-0.24
CAVIN2	0.28	0.28	0.5	0.17	0.23	-0.19	-0.22
AFAP1	0.33	0.4	0.53	0.34	0.37	-0.4	-0.42
OSMR	0.34	0.41	0.54	0.28	0.45	-0.37	-0.29
LOXL3	0.37	0.46	0.51	0.35	0.43	-0.46	-0.31
TRPS1	0.15	0.28	0.59	0.42	0.48	-0.43	-0.42
FAM241A	0.17	0.28	0.57	0.19	0.33	-0.3	-0.25
PLCE1	0.23	0.33	0.64	0.42	0.45	-0.4	-0.28
SCN9A	0.16	0.25	0.6	0.43	0.36	-0.33	-0.24
ZEB1	0.35	0.43	1	0.47	0.45	-0.41	-0.4
C5ORF58	0.16	0.33	0.57	0.34	0.33	-0.29	-0.24
TENM1	0.17	0.25	0.5	0.25	0.29	-0.24	-0.19
ARHGEF6	0.19	0.42	0.6	0.33	0.51	-0.32	-0.28
DPYD	0.14	0.43	0.59	0.39	0.55	-0.37	-0.29
MAP1B	0.13	0.3	0.52	0.3	0.4	-0.34	-0.19
SLIT2	0.21	0.37	0.55	0.44	0.46	-0.27	-0.26
CRIM1	0.23	0.35	0.55	0.37	0.48	-0.36	-0.33
ZFPM2	0.23	0.29	0.52	0.31	0.39	-0.26	-0.25
ST6GALNAC5	0.25	0.37	0.54	0.35	0.39	-0.3	-0.16
NFE2L3	0.24	0.33	0.53	0.28	0.39	-0.31	-0.21
SLC14A1	0.19	0.3	0.52	0.23	0.36	-0.31	-0.2
CEP126	0.18	0.27	0.51	0.25	0.19	-0.29	-0.27
DEPTOR	0.11	0.33	0.51	0.29	0.22	-0.3	-0.3
LPAR1	0.46	0.48	0.65	0.27	0.4	-0.43	-0.2
CPE	0.47	0.35	0.63	0.27	0.38	-0.41	-0.23
EBF1	0.44	0.33	0.6	0.23	0.34	-0.38	-0.23
MEOX2	0.5	0.34	0.52	0.23	0.32	-0.35	-0.2
ADAMTS5	0.49	0.3	0.59	0.29	0.4	-0.35	-0.25
HECW2	0.44	0.31	0.59	0.27	0.45	-0.33	-0.19
VCAN	0.49	0.32	0.56	0.33	0.47	-0.37	-0.21
S1PR3	0.5	0.32	0.52	0.22	0.46	-0.36	-0.23
CLMP	0.41	0.32	0.52	0.23	0.47	-0.31	-0.22
FLT1	0.48	0.41	0.58	0.29	0.42	-0.33	-0.27
PLPP3	0.47	0.37	0.63	0.24	0.42	-0.33	-0.19
TRPC6	0.42	0.34	0.52	0.22	0.34	-0.25	-0.16
THSD7A	0.41	0.33	0.51	0.24	0.42	-0.23	-0.15
TCF4	0.39	0.39	0.67	0.32	0.5	-0.36	-0.24
PALLD	0.33	0.32	0.56	0.31	0.41	-0.36	-0.18
NRP1	0.46	0.39	0.69	0.33	0.61	-0.46	-0.29
MECOM	0.35	0.24	0.53	0.25	0.44	-0.31	-0.22
MAP3K7CL	0.3	0.29	0.58	0.24	0.48	-0.4	-0.22
ZNF204P	0.29	0.22	0.5	0.18	0.37	-0.32	-0.2
EDNRA	0.43	0.19	0.54	0.26	0.42	-0.34	-0.18
PRRX1	0.4	0.26	0.63	0.3	0.41	-0.38	-0.19
TFPI	0.38	0.26	0.56	0.23	0.38	-0.32	-0.24
TCEAL7	0.35	0.22	0.51	0.22	0.34	-0.28	-0.18
TCIM	0.4	0.21	0.58	0.29	0.44	-0.32	-0.21
LOX	0.38	0.23	0.5	0.28	0.4	-0.28	-0.2
PLN	0.34	0.21	0.51	0.29	0.38	-0.24	-0.2
DPYSL3	0.42	0.34	0.57	0.34	0.41	-0.45	-0.27
SEMA3A	0.44	0.34	0.61	0.34	0.34	-0.39	-0.28
ARHGAP6	0.32	0.3	0.52	0.3	0.31	-0.35	-0.27
LPAR4	0.39	0.27	0.52	0.36	0.34	-0.36	-0.27

ANKH	0.47	0.27	0.55	0.25	0.36	-0.45	-0.27
TMEM100	0.43	0.28	0.54	0.26	0.4	-0.43	-0.25
ADAMTS9	0.55	0.39	0.57	0.34	0.45	-0.47	-0.31
GAP43	0.46	0.27	0.51	0.34	0.38	-0.37	-0.23
CALCRL	0.35	0.33	0.61	0.28	0.44	-0.26	-0.16
FGF7	0.31	0.33	0.61	0.3	0.42	-0.25	-0.19
FILIP1	0.32	0.34	0.55	0.24	0.36	-0.19	-0.19
PCDH17	0.35	0.35	0.59	0.36	0.41	-0.22	-0.15
EPB41L2	0.29	0.4	0.54	0.32	0.4	-0.22	-0.14
ANGPTL1	0.27	0.24	0.56	0.28	0.33	-0.18	-0.1
FHL5	0.25	0.25	0.51	0.2	0.3	-0.12	-0.16
C1QTNF3	0.37	0.27	0.57	0.29	0.22	-0.21	-0.14
PCDH18	0.39	0.28	0.53	0.29	0.3	-0.21	-0.14
RGS4	0.38	0.3	0.53	0.27	0.35	-0.28	-0.13
FAM13C	0.34	0.24	0.53	0.24	0.32	-0.24	-0.11
RASSF9	0.3	0.23	0.52	0.21	0.28	-0.24	-0.1
KIAA0040	0.32	0.43	0.55	0.14	0.46	-0.44	-0.2
PLAGL1	0.4	0.38	0.64	0.22	0.54	-0.42	-0.23
SLC16A14	0.38	0.37	0.57	0.2	0.51	-0.39	-0.18
PRTFDC1	0.36	0.28	0.55	0.15	0.47	-0.33	-0.21
LGI2	0.36	0.37	0.57	0.18	0.42	-0.33	-0.16
GUCY1A3	0.37	0.32	0.59	0.22	0.47	-0.32	-0.14
CYYR1	0.36	0.28	0.58	0.18	0.45	-0.27	-0.13
PDGFRA	0.35	0.28	0.53	0.22	0.45	-0.33	-0.09
RASGRF2	0.35	0.27	0.55	0.2	0.51	-0.3	-0.16
JCAD	0.37	0.26	0.51	0.19	0.5	-0.33	-0.12
TGFB3	0.5	0.3	0.56	0.17	0.44	-0.42	-0.19
MYCT1	0.4	0.22	0.51	0.14	0.41	-0.29	-0.11
MEDAG	0.45	0.24	0.52	0.19	0.53	-0.41	-0.21
SCARF1	0.44	0.32	0.51	0.15	0.54	-0.36	-0.17
BCAT1	0.36	0.26	0.55	0.23	0.56	-0.33	-0.3
DSE	0.34	0.27	0.53	0.26	0.58	-0.43	-0.24
FAM131B	0.42	0.33	0.51	0.29	0.58	-0.5	-0.2
PRDM8	0.28	0.32	0.53	0.22	0.57	-0.42	-0.18
TLR2	0.26	0.31	0.5	0.16	0.54	-0.34	-0.2
PTPRE	0.36	0.38	0.5	0.14	0.6	-0.48	-0.21
ADGRL4	0.38	0.34	0.65	0.22	0.47	-0.28	-0.22
ECM2	0.27	0.29	0.59	0.2	0.44	-0.24	-0.18
COLEC12	0.31	0.31	0.57	0.21	0.45	-0.29	-0.25
PIWIL4	0.24	0.28	0.5	0.16	0.35	-0.26	-0.18
RHOU	0.34	0.43	0.62	0.22	0.49	-0.33	-0.26
CD1D	0.24	0.36	0.51	0.21	0.46	-0.28	-0.24
INHBA	0.35	0.29	0.58	0.38	0.52	-0.33	-0.22
SULF1	0.31	0.27	0.53	0.32	0.54	-0.24	-0.16
TMEM200A	0.29	0.34	0.61	0.26	0.6	-0.33	-0.17
SEL1L3	0.25	0.34	0.52	0.23	0.5	-0.3	-0.16
HGF	0.36	0.35	0.6	0.31	0.56	-0.32	-0.2
VGLL3	0.25	0.3	0.58	0.28	0.48	-0.27	-0.17
LRRC4C	0.24	0.3	0.5	0.28	0.43	-0.26	-0.13
CYP1B1	0.24	0.27	0.53	0.17	0.48	-0.22	-0.14
NEXN	0.21	0.23	0.52	0.19	0.44	-0.27	-0.16
NHS	0.28	0.22	0.51	0.28	0.52	-0.32	-0.17
CALHM5	0.3	0.28	0.5	0.25	0.6	-0.32	-0.12
CDH11	0.3	0.27	0.5	0.23	0.52	-0.29	-0.11

ABCC9	0.45	0.4	0.56	0.17	0.34	-0.18	-0.1
KL	0.35	0.4	0.54	0.16	0.35	-0.2	-0.13
SORL1	0.37	0.49	0.51	0.18	0.27	-0.28	-0.09
FBN1	0.36	0.34	0.56	0.26	0.44	-0.24	-0.07
TSHZ2	0.38	0.44	0.59	0.25	0.54	-0.31	-0.13
CFH	0.26	0.35	0.52	0.22	0.4	-0.26	-0.1
RARRES1	0.25	0.36	0.52	0.19	0.46	-0.23	-0.12
NHSL2	0.4	0.4	0.5	0.18	0.43	-0.27	-0.12
SYNPO2	0.34	0.34	0.5	0.14	0.39	-0.25	-0.08
ITGA8	0.29	0.44	0.53	0.21	0.39	-0.18	-0.12
ITGA4	0.33	0.38	0.52	0.26	0.44	-0.16	-0.16
SGMS1	0.11	0.27	0.62	0.28	0.55	-0.33	-0.2
CMAHP	0.14	0.27	0.56	0.19	0.47	-0.24	-0.16
MAN1A1	0.21	0.42	0.59	0.24	0.57	-0.23	-0.14
GCNT1	0.18	0.43	0.57	0.19	0.47	-0.26	-0.18
ST8SIA4	0.19	0.38	0.6	0.24	0.63	-0.31	-0.23
FAM105A	0.16	0.35	0.57	0.17	0.63	-0.3	-0.17
ATP8B4	0.17	0.33	0.55	0.18	0.59	-0.25	-0.16
SLC8A1	0.19	0.33	0.5	0.19	0.6	-0.21	-0.15
CCDC170	0.2	0.32	0.53	0.12	0.53	-0.24	-0.14
P2RY14	0.21	0.33	0.5	0.13	0.47	-0.2	-0.1
TM6SF1	0.18	0.32	0.59	0.26	0.48	-0.17	-0.21
GPR34	0.12	0.36	0.55	0.18	0.49	-0.15	-0.21
RGS18	0.12	0.33	0.52	0.16	0.49	-0.18	-0.17
TLR4	0.12	0.3	0.5	0.22	0.51	-0.11	-0.13
ARHGEF3	0.09	0.31	0.54	0.14	0.44	-0.21	-0.06
GLIPR1	0.28	0.33	0.66	0.27	0.63	-0.46	-0.29
ANTXR2	0.25	0.27	0.59	0.33	0.52	-0.42	-0.26
SLC9A7	0.24	0.34	0.52	0.29	0.5	-0.4	-0.22
RTN1	0.2	0.37	0.56	0.3	0.64	-0.4	-0.24
KCNMA1	0.2	0.33	0.53	0.26	0.62	-0.43	-0.24
EMB	0.13	0.28	0.5	0.2	0.57	-0.33	-0.17
COL8A1	0.29	0.26	0.55	0.43	0.57	-0.37	-0.28
RUNX1	0.21	0.21	0.5	0.34	0.57	-0.4	-0.23
RGS5	0.39	0.36	0.53	0.24	0.19	-0.22	-0.18
FREM1	0.38	0.39	0.52	0.22	0.24	-0.24	-0.14
PPP1R9A	0.29	0.35	0.5	0.26	0.11	-0.17	-0.12
ATP2B1-AS1	0.44	0.34	0.52	0.29	0.17	-0.36	-0.28
SPRY4	0.33	0.42	0.5	0.33	0.14	-0.28	-0.25
TOP1	0.33	0.42	0.51	0.32	0.18	-0.04	-0.24
MARCH1	0.25	0.4	0.54	0.2	0.5	-0.04	0.01
SLC40A1	0.18	0.38	0.5	0.05	0.36	-0.12	-0.07
ZEB1-AS1	0.16	0.25	0.79	0.36	0.2	-0.26	-0.3
ANKAR	0.17	0.27	0.53	0.19	0.13	-0.11	-0.18
BACH1	0.23	0.5	0.62	0.43	0.37	-0.24	-0.29
DIXDC1	0.18	0.39	0.56	0.41	0.35	-0.22	-0.29
GOLIM4	0.18	0.36	0.54	0.39	0.28	-0.13	-0.29
UBXN4	0.18	0.34	0.59	0.27	0.26	-0.13	-0.24
CASC4	0.15	0.34	0.65	0.36	0.25	-0.22	-0.23
PLOD2	0.14	0.24	0.51	0.32	0.19	-0.21	-0.2
CMTM1	0.18	0.36	0.59	0.4	0.25	-0.18	-0.25
LEPR	0.16	0.33	0.52	0.3	0.23	-0.17	-0.19
HECTD2	0.1	0.31	0.63	0.38	0.28	-0.23	-0.36
THAP2	0.12	0.32	0.56	0.41	0.21	-0.18	-0.32

TABLE S1

BIRC2	0.08	0.32	0.5	0.32	0.22	-0.15	-0.33
POGLUT1	0.16	0.25	0.55	0.35	0.23	-0.16	-0.27
PXYLP1	0.19	0.25	0.54	0.4	0.28	-0.24	-0.31
ZFP37	0.16	0.28	0.52	0.38	0.23	-0.22	-0.33
MSANTD4	0.17	0.28	0.54	0.3	0.12	-0.13	-0.25
ETV1	0.15	0.35	0.55	0.32	0.12	-0.11	-0.19
ARRDC3	0.16	0.31	0.53	0.38	0.19	-0.1	-0.22
TMEM167A	0.12	0.27	0.51	0.35	0.12	-0.08	-0.18
ARID4B	0.07	0.43	0.56	0.32	0.19	-0.07	-0.3
USP34	0.1	0.37	0.52	0.28	0.17	-0.08	-0.25
HSPA13	0.12	0.33	0.54	0.37	0.25	-0.07	-0.26
SOCS5	0.09	0.34	0.54	0.36	0.22	-0.1	-0.29
CCP110	0.08	0.31	0.52	0.33	0.2	-0.07	-0.25
PPP1R12A	0.14	0.39	0.57	0.36	0.23	-0.07	-0.23
ATF1	0.13	0.31	0.5	0.28	0.19	-0.07	-0.2
RGPD4	0.15	0.36	0.5	0.26	0.19	-0.08	-0.22
SMC5	0.13	0.32	0.5	0.28	0.16	-0.08	-0.24
MIS18BP1	0.13	0.3	0.51	0.28	0.17	-0.02	-0.22
SP3	0.17	0.36	0.5	0.32	0.2	-0.05	-0.28
DNAJC10	0.22	0.32	0.52	0.31	0.17	-0.04	-0.23
GABPB1	0.22	0.31	0.51	0.31	0.23	-0.12	-0.31
SACS	0.18	0.3	0.51	0.35	0.17	-0.1	-0.3
CWC22	0.17	0.26	0.51	0.31	0.17	-0.08	-0.31
ITGB1	0.04	0.2	0.55	0.38	0.3	-0.1	-0.32
UFM1	0.09	0.2	0.5	0.35	0.21	-0.1	-0.29
C16ORF52	0.12	0.25	0.52	0.42	0.14	-0.16	-0.35
PIBF1	0.14	0.25	0.54	0.36	0.13	-0.07	-0.32
GORAB	0.12	0.24	0.52	0.32	0.11	-0.06	-0.26
CEP290	0.08	0.23	0.52	0.28	0.15	-0.09	-0.28
ZNF639	0.1	0.18	0.5	0.3	0.15	-0.11	-0.3
JMJD1C	-0.02	0.31	0.57	0.36	0.3	-0.12	-0.33
RUFY2	-0.03	0.2	0.5	0.22	0.2	-0.08	-0.26
SEC62	0.03	0.25	0.52	0.27	0.22	-0.01	-0.22
PHACTR2	0.21	0.46	0.61	0.35	0.38	-0.21	-0.17
STARD4	0.2	0.35	0.51	0.23	0.27	-0.19	-0.16
ENPP4	0.17	0.31	0.58	0.23	0.3	-0.2	-0.18
NTN4	0.2	0.3	0.56	0.24	0.33	-0.23	-0.19
RAP1A	0.17	0.34	0.52	0.24	0.36	-0.23	-0.21
RORA	0.15	0.29	0.5	0.16	0.26	-0.22	-0.12
CD302	0.23	0.41	0.52	0.21	0.29	-0.19	-0.24
SLC12A6	0.12	0.38	0.5	0.24	0.34	-0.29	-0.15
DOCK11	0.14	0.47	0.57	0.27	0.41	-0.2	-0.14
RGPD1	0.14	0.37	0.52	0.19	0.29	-0.11	-0.17
HCFC2	0.07	0.33	0.51	0.23	0.28	-0.12	-0.14
ITPR1	0.09	0.31	0.5	0.24	0.29	-0.15	-0.14
FUT8	0.14	0.34	0.61	0.39	0.42	-0.22	-0.19
FNDC3B	0.2	0.32	0.57	0.35	0.4	-0.17	-0.25
CPED1	0.15	0.26	0.53	0.32	0.39	-0.14	-0.2
UHRF1BP1L	0.09	0.34	0.59	0.31	0.32	-0.16	-0.25
ZCCHC6	0.11	0.32	0.53	0.31	0.37	-0.17	-0.24
RAP2C	0.13	0.28	0.54	0.29	0.32	-0.19	-0.24
TANK	0.11	0.3	0.52	0.3	0.32	-0.21	-0.27
MCL1	0.08	0.32	0.5	0.34	0.42	-0.2	-0.23
SMURF2	0.06	0.31	0.5	0.4	0.36	-0.17	-0.29

SKIL	0.09	0.3	0.57	0.4	0.35	-0.09	-0.23
STAM2	0.06	0.27	0.5	0.29	0.26	-0.06	-0.21
SPOPL	0.02	0.35	0.55	0.34	0.31	-0.15	-0.24
KIAA0825	0.07	0.32	0.53	0.36	0.29	-0.13	-0.21
GOLPH3	0.04	0.29	0.5	0.3	0.25	-0.1	-0.14
CMTM6	0.09	0.29	0.5	0.24	0.34	-0.09	-0.21
GALNT4	0.03	0.33	0.5	0.24	0.31	-0.16	-0.22
NAIP	0.01	0.31	0.5	0.27	0.33	-0.15	-0.22
UTRN	0	0.34	0.5	0.3	0.35	-0.15	-0.13
DDHD1	0.04	0.17	0.5	0.23	0.25	-0.14	-0.12
P2RY1	0.19	0.39	0.53	0.46	0.33	-0.2	-0.18
ITGA2	0.11	0.42	0.5	0.4	0.34	-0.23	-0.12
CREB5	0.13	0.33	0.51	0.43	0.19	-0.22	-0.17
KDM7A	0.13	0.5	0.56	0.31	0.24	-0.14	-0.19
CEP85L	0.13	0.43	0.59	0.31	0.21	-0.13	-0.21
NFAT5	0.1	0.39	0.51	0.28	0.13	-0.12	-0.15
ATP11C	0.1	0.42	0.58	0.35	0.27	-0.1	-0.22
XIAP	0.08	0.39	0.56	0.28	0.24	-0.09	-0.2
MANEA	0.05	0.34	0.55	0.3	0.26	-0.07	-0.19
SCAF11	0.11	0.43	0.56	0.3	0.27	-0.04	-0.22
ZNF41	0.05	0.44	0.53	0.34	0.19	-0.05	-0.2
SP4	0.05	0.47	0.53	0.31	0.23	-0.04	-0.22
ITGAV	0.06	0.38	0.54	0.37	0.25	-0.01	-0.19
AKAP11	0.04	0.35	0.56	0.34	0.22	0	-0.2
ZNF148	0.02	0.35	0.51	0.31	0.19	-0.01	-0.22
CEP120	-0.01	0.37	0.55	0.31	0.28	-0.06	-0.22
FAM208A	0	0.34	0.51	0.33	0.23	-0.05	-0.22
SLC5A3	0.08	0.42	0.55	0.36	0.26	-0.15	-0.25
RASA1	0.03	0.39	0.54	0.35	0.24	-0.14	-0.28
MTX3	0.02	0.31	0.52	0.38	0.15	-0.14	-0.24
LIMS1	0.15	0.38	0.5	0.31	0.31	-0.06	-0.21
ATRX	-0.01	0.42	0.54	0.31	0.14	0.02	-0.19
ARID4A	-0.01	0.34	0.51	0.28	0.14	0.03	-0.19
NAB1	0.05	0.34	0.54	0.34	0.1	-0.05	-0.2
GABPA	0.04	0.3	0.52	0.31	0.15	-0.02	-0.23
SENP7	0.01	0.32	0.51	0.3	0.1	-0.01	-0.22
FAM214A	0.05	0.33	0.52	0.2	0.11	-0.05	-0.18
CTDSPL2	0.12	0.37	0.51	0.34	0.08	0.01	-0.21
PDS5B	0.12	0.4	0.5	0.41	0.16	0	-0.25
VPS13C	-0.01	0.47	0.54	0.22	0.23	-0.02	-0.09
DENND4A	-0.02	0.48	0.56	0.27	0.29	-0.03	-0.18
NR3C1	-0.01	0.46	0.53	0.26	0.25	-0.07	-0.18
CCDC186	0	0.39	0.51	0.23	0.21	-0.04	-0.2
AFTPH	0	0.41	0.51	0.21	0.25	-0.11	-0.15
CCPG1	-0.03	0.34	0.5	0.18	0.22	-0.07	-0.11
MBNL1	-0.04	0.31	0.51	0.25	0.23	-0.08	-0.16
KIAA1551	0.08	0.45	0.5	0.15	0.23	-0.07	-0.17
RSPRY1	0.13	0.42	0.51	0.34	0.18	-0.02	-0.13
ARL15	0.09	0.36	0.51	0.3	0.2	-0.04	-0.05
CAB39	0.1	0.39	0.51	0.23	0.25	0	-0.11
UBR1	0.05	0.42	0.5	0.26	0.19	0.06	-0.1
LMBRD2	-0.02	0.42	0.5	0.29	0.17	0.02	-0.1
IL6ST	0.01	0.57	0.5	0.29	0.2	0.04	-0.16
MIA2	0.01	0.19	0.51	0.25	0.22	0.13	0

Table S2. Upstream regulator analysis by IPA on differentially expressed genes in Me71 NFATc2-shRNA_86a transfectant compared to two control transfectants

A. NFATc2 shRNA_86a compared to Control_shRNA_1

Upstream Regulator	Log Ratio	Molecule Type	Predicted Activation State	Activation z-score	p-value of overlap	Target molecules in dataset
TBX2	0.298	transcription regulator	Inhibited	-5.020	3.00E-13	ANLN,ASF1B,AURKA,AURKB,BHLHE40,BUB1,CCNA2,CCNL1,CCNL2,CDC6,CDCA3,CDCA5,CDH1,CDK1,CDKN1A,CDT1,CHAF1B,CHEK1,CKAP2,CKS1B,DDIT3,DNMT1,E2F7,E2F8,EZH2,FAM120B,FOXM1,HAUS1,HELLS,LIG1,MAD2L1,MCM2,MCM4,MCM5,MCM7,MED20,MXD3,NCAPD2,NCAPG2,PARD6A,PCGF6,PKMYT1,PLK1,RBL1,SGOL1,SMC2,SRF,TCEA2,TIPIN,TYRP1,ZNF329
**FOXM1**	**-0.882**	transcription regulator	Inhibited	-4.166	2.15E-06	ADAM17,ANXA1,ATF2,AURKB,BIRC5,BUB1B,CAV1,CCNA2,CCNB1,CCNB2,CCND1,CCNE2,CCNF,CDC20,CDC25A,CDC25B,CDH1,CDH2,CDK1,CDKN1A,CDKN3,CENPA,CENPF,CKS1B,FOXM1,GTSE1,KDR,KIF20A,MYC,NEK2,PLK1,PLK4,SKP2,SNAI2,STMN1,TJP1,TP53,ZEB2
**MYC**	**-0.517**	transcription regulator	Inhibited	-3.998	1.70E-12	ACSL4,ACTN1,ACTN4,ADARB1,ADD1,ADK,ADM,AK2,AKAP12,ALCAM,AMD1,ANGPT1,ARL6IP1,ASNS,ATPIF1,AURKB,BAX,BCKDHB,BCL2,BCL2A1,BCL2L1,BIN1,BIRC2,BIRC5,BMI1,BRCA1,BRD2,BUB1,BUB1B,CANX,CASP1,CASP9,CAV1,CCNA2,CCNB1,CCNB2,CCND1,CCND3,CCNE2,CCNG2,CD44,CD47,CD9,CDC20,CDC25A,CDC25B,CDC25C,CDH1,CDH2,CDK1,CDK6,CDK7,CDKN1A,CFLAR,CHEK1,CHKA,CKS2,COL15A1,COL4A2,COL5A2,COL6A3,COX5B,COX6A1,CPD,CPT1A,CPT2,CRIP2,CRYAB,CSDE1,CSRP2,CSTB,CTNNB1,CTSB,CTSD,CTSV,CUL1,CYFIP2,DBN1,DDIT3,DDX18,DDX5,DFFB,DKK1,DLEU2,DNMT1,DNPH1,DSTN,DUSP6,E2F2,E2F3,ECM1,EED,EGR1,EIF2B4,EIF2S2,EIF3D,EIF4A1,EIF4G1,EMP1,ENO1,EXOSC8,EZH2,F2R,FABP5,FAM129A,FAP,FBL,FBLN5,FBXO32,FCGRT,FN1,FOSL1,FOXM1,FSTL1,FTH1,FXYD1,GAA,GABARAP,GADD45A,GAMT,GAPDH,GAR1,GCLC,GCLM,GCSH,GGH,GLG1,GLS,GLUD1,GOLGA2,GOT2,GTF2F2,H2AFZ,HAS2,HBE1,HIST1H4A (includes others),HK2,HLA-B,HLA-E,HMGN2,HMOX1,HNRNPA1,HNRNPAB,HSPA14,HSPA1A/HSPA1B,HSPB1,HSPD1,HSPE1,ICAM1,ID2,ID3,IER3,IFI35,IFIT1,IL10,IL1RAP,IL8,IMPA2,IREB2,IRF7,IRF9,ITGA6,ITGB1,KDR,KLF10,KLF4,KLF6,LAMB2,LDHA,LIN28B,LSM4,LXN,MAD2L1,MAN2A1,MAT2A,MBP,MCM5,MCM7,MGAT1,MIF,MINA,MITF,MKI67,MOGS,MRE11A,MRPL12,MSH2,MSN,MTBP,MTFR2,MTHFD1,MYC,MYO1C,NBN,NCL,NDEL1,NDRG1,NDRG2,NFATC3,NGFRAP1,NME1,NME2,NOP56,OAS1,ODC1,OLIG1,PAICS,PAM,PCNA,PERP,PFKFB3,PFKL,PFKM,PFKP,PGAM1,PGK1,PHB,PKM,PLA1A,PLAUR,PLK1,PMP22,PNCK,POLD1,POLR1B,PPP1R15A,PRDX3,PREP,PRKACB,PRMT1,PRODH,PROM1,PSAT1,PTEN,RAD51,RANBP1,RARA,RBBP4,RCC1,RHOB,ROCK2,RPL13,RPL21,RPS15A,RPS18,RRM2,RRM2B,RRS1,RTN2,RUVBL2,SAT1,SCPEP1,SDCBP,SEPHS2,SFRP1,SGK1,SHMT1,SKP2,SLC25A19,SLC2A3,SLC3A2,SLC7A5,SNRPC,SNRPD1,SNRPN,SOD2,SOX6,SP3,SPN,SPP1,ST3GAL1,ST3GAL3,STMN1,TAGLN2,TES,TF,TFDP1,THBS1,TMEM97,TMSB10/TMSB4X,TNC,TNFRSF10B,TP53,TP53I3,TPD52,TRIP12,TSPAN7,TYMS,UBE2C,UXT,VAMP3,XRCC3,YRDC,ZFP36,ZFP36L1,ZIC2,ZXDB
E2F1		transcription regulator	Inhibited	-3.726	8.85E-20	AMY2A,AURKA,AURKB,BAX,BCL2,BCL2L11,BIRC5,BMI1,BMP4,BOK,BRCA1,BUB3,CALD1,CALM1 (includes others),CASP7,CASP9,CAV1,CBX5,CCNA2,CCNB1,CCNB2,CCND1,CCND3,CCNE2,CCNO,CD9,CDC25A,CDC45,CDC6,CDCA4,CDK1,CDKN1A,CDKN2D,CFLAR,CHEK1,CHEK2,CLIC1,COPS8,COX7C,CRABP2,CRYAB,CSDE1,CTNNBIP1,CTSB,DBF4,DDAH2,DGKI,DHFR,DLEU2,DNMT1,DUSP10,DUSP4,DUT,E2F2,E2F3,E2F4,ECT2,EED,EGR1,EIF3I,ENO2,EXOSC9,EYA4,EZH2,FBXO5,FEN1,FGFR1,FHIT,FOXM1,GMNN,GPS2,H19,HADHA,HELLS,HES1,HIST1H2AC,HIST1H2BJ/HIST1H2BK,HIST1H4A (includes others),HIST2H2AA3/HIST2H2AA4,HLTF,HMGB1,HMGB2,HRK,HSPA1A/HSPA1B,HSPE1,ICAM1,ID3,IRS1,JMY,KDR,KIAA0101,KIF23,KRT10,LACTB,MAD2L1,MAF,MAP3K14,MAP3K5,MCL1,MCM10,MCM2,MCM4,MCM5,MCM7,MEIS1,MLH1,MMP16,MSH2,MT1G,MTHFD1,MYB,MYC,NCL,NCOA3,NDC80,NRIP1,NRP1,PA2G4,PAWR,PCNA,PHB,PMPCB,POLA2,POLD1,PPP1R13B,PPP1R8,PRDM2,PRIM2,PRKDC,PSAT1,PSMB9,PTPN4,RACGAP1,RAD51,RANBP1,RBBP4,RBBP8,RBL1,RFC2,RFC3,RFC4,RPA3,RRM1,RRM2,SIVA1,SLC3A2,SMC4,SNRPC,SOD2,STAM,STMN1,TAP2,TAPBP,TK1,TOP2A,TOP2B,TP53,TP53BP2,TP53INP1,TRAF2,TYMS,UNG,UXT,YWHAE,ZFP36,ZNF160,ZNF2[illegible]
E2F3	-0.243	transcription regulator	Inhibited	-3.568	3.11E-08	BIRC5,BMI1,BOK,CCNA2,CCNB1,CCND1,CCND3,CDC25A,CDC45,CDC6,CDCA4,CDK1,CDKN1A,DHFR,E2F2,E2F3,EED,EZH2,FBXO5,MCM10,MCM2,MCM4,MCM5,MCM7,MLH1,MT1G,MYB,MYC,PCNA,POLA2,PPP1R13B,PPP1R8,RAD51,RBL1,RRM1,STAG3,TK1,TOP2B,TP53BP2,UXT
**EZH2**	**-0.935**	transcription regulator	Inhibited	-3.067	1.69E-02	AADAT,AMACR,APPBP2,AXIN2,BCL2,BCL2L11,BIRC3,BMP4,BRCA1,CCND1,CCND3,CCNE2,CD82,CDH1,CDK6,CDKN1A,CPD,CTNNB1,CX3CL1,CXCL1,DDT,DKK1,EFNA1,EZH2,FBLN1,FHOD3,FOXF2,FRZB,FUCA1,GDF15,GSTM4,HOXB13,HOXB5,HOXB6,ICAM1,ICOSLG,IGFBP3,IL24,IL6,IL8,ITGA1,KIAA1199,KLF4,MAL2,MYC,MZF1,NME5,OXTR,PAX3,PER1,PIM2,PKMYT1,PLA2G4A,PPP2R2B,PTGES,PTGS2,RAC3,RAP1GAP,SATB1,SCAMP2,TBX3,TP53,TRIM38,ZMYM4
E2F2	-0.299	transcription regulator	Inhibited	-2.778	1.45E-08	AMY2A,BCL2,BIRC5,BMI1,CCNA2,CCNB1,CCND1,CCND3,CDC25A,CDC45,CDC6,CDCA4,CDK1,CDKN1A,CDKN2D,DHFR,E2F3,EED,EZH2,JMY,MCM10,MCM2,MCM4,MCM5,MCM7,MLH1,MT1G,MYB,MYC,PCNA,RAD51,RBL1,RRM2,TOP2B,TP53,UXT
SREBF2		transcription regulator	Inhibited	-2.595	1.05E-04	AACS,ABCA1,ACSL1,ACSS2,ALDOC,CDKN1A,CYP51A1,DHCR7,EBP,FABP5,FABP6,FDFT1,G6PD,GTF2I,HES6,HMGCS1,HSD17B1,IDH1,IDI1,INSIG1,LDLR,LSS,MSMO1,MVK,NSDHL,PLIN2,RDH11,SC5D,SCD,SQLE,SREBF1,TFF3,TM7SF2,TMEM97
MBD2		transcription regulator	Inhibited	-2.469	6.61E-02	AMY1A (includes others), ATP5I,BIRC3,BRCA1,CDH1,CDKN1A,CENPH,CKS2,G6PD,HK2,MEF2A,MGMT,NBR2,NEIL3,ORC6,PLA2G16,PLK1,POLB,PTPN4,RELB,TFF2
RCAN1	0.269	transcription regulator	Inhibited	-2.401	3.38E-01	CCNA2,CCNF,ICAM1,IL6,NOS3,PTGS2,RCAN1
KLF5		transcription regulator	Inhibited	-2.385	1.64E-01	ACADM,BIRC5,CCND1,CDK1,CDKN1A,CDT1,CTNNB1,E2F3,FAM110A,LAMA1,MYC,NOTCH1,PCNA,PPARG,TBC1D14,WFS1
TLX1		transcription regulator	Inhibited	-2.236	5.51E-01	CDC6,CHEK1,KDR,MCL1,MYB,PCNA,RAD51
MYBL2		transcription regulator	Inhibited	-2.140	9.58E-03	BCL2,BCL2L11,BIRC3,BIRC5,CCNA2,CCNB1,CCNB2,CCND1,CDC25B,CDK1,IGFBP5,IQGAP1,ITGB1,MAD1L1,MYC,PPP3CA,TOP2A
CIITA	-0.729	transcription regulator	Inhibited	-2.082	2.25E-01	CCND1,CD74,GCNT2,HLA-B,HLA-DMB,HLA-DPA1,HLA-DQB1,HLA-DRA,IL10
FLI1		transcription regulator	Inhibited	-2.028	5.19E-01	CCND1,CCND3,CTGF,DNMT1,EGR1,ID2,IMP4,NHP2L1,NIP7,RARB,SNRPB,SRGN,TCP1,TNC

Table S2. Upstream regulator analysis by IPA on differentially expressed genes in Me71 NFATc2-shRNA_86a transfectant compared to two control transfectants

B. NFATc2 shRNA_86a compared to Control_shRNA_2

Upstream Regulator	Log Ratio	Molecule Type	Predicted Activation State	Activation z-score	p-value of overlap	Target molecules in dataset
TBX2		transcription regulator	Inhibited	-4.933	1.90E-15	ANLN,ASF1B,ATF3,AURKA,AURKB,BHLHE40,BUB1,CCNA2,CCNL1,CCNL2,CDC6,CDCA3,CDCA5,CDK1,CDKN1A,CDKN2C,CDT1,CHAF1B,CHEK1,CKAP2,CKS1B,DBP,DNMT1,E2F7,E2F8,EZH2,FAM120B,FOXM1,HAUS1,HELLS,HEY1,LIG1,MAD2L1,MCM2,MCM4,MCM5,MCM7,MED20,MXD3,NCAPD2,NCAPG2,PARD6A,PAXBP1,PCGF6,PKMYT1,PLK1,PRC1,RBL1,RBM39,SGOL1,SMC2,SRF,TCEA2,TIPIN,TYRP1,ZNF329
**FOXM1**	**-0.961**	transcription regulator	Inhibited	-4.37	2.42E-05	ANXA1,ATF2,AURKB,BIRC5,BUB1B,CCNA2,CCNB1,CCNB2,CCND1,CCNE1,CCNE2,CCNF,CDC20,CDC25A,CDC25B,CDK1,CDK2,CDKN1A,CDKN3,CENPA,CENPF,CKS1B,FOXM1,GTSE1,KDR,KIF20A,MYC,NEDD4,NEK2,PLK1,PLK4,PRC1,SKP2,SNAI2,STMN1,TJP1,TP53,ZEB2
**MYC**	**-0.58**	transcription regulator	Inhibited	-4.228	2.33E-13	ACSL4,ACTN1,ACTN4,ADARB1,ADD1,ADIPOR1,ADK,ADM,AK2,AKAP12,ALCAM,AMD1,ANGPT1,ARL6IP1,ASNS,ATF4,ATPIF1,AURKB,BAX,BCKDHB,BCL2,BCL2A1,BCL2L1,BIN1,BIRC2,BIRC5,BMI1,BOP1,BRCA1,BUB1,BUB1B,CANX,CASP1,CASP9,CAST,CCNA2,CCNB1,CCNB2,CCND1,CCND3,CCNE1,CCNE2,CCNG2,CD44,CD47,CD9,CDC20,CDC25A,CDC25B,CDC25C,CDK1,CDK11A/CDK11B,CDK2,CDK6,CDK7,CDKN1A,CEBPD,CFLAR,CHEK1,CHKA,CKS2,COL15A1,COL4A2,COL5A2,COL6A3,COX5B,COX6A1,CPD,CPT1A,CPT2,CRIP2,CRYAB,CSRP2,CTBS,CTNNB1,CTSB,CTSD,CTSV,CUL1,CYFIP2,DBI,DBN1,DDX18,DDX21,DDX5,DKK1,DLEU2,DNMT1,DNPH1,DSTN,DUSP6,E2F2,E2F3,ECM1,EED,EGLN1,EGR1,EIF2B4,EIF2S1,EIF2S2,EIF3D,EIF3G,EIF4G1,EMP1,ENO1,ERBB2,EXOSC8,EZH2,F2R,FABP5,FAM129A,FAP,FBL,FBLN5,FBXO32,FCGRT,FOSL1,FOXM1,FSTL1,FTH1,FXYD1,GAA,GADD45A,GAMT,GAPDH,GAR1,GAS1,GCLC,GCSH,GGH,GLG1,GLS,GLUD1,GM2A,GOLGA2,GPI,GTF2F2,H2AFZ,HAS2,HBE1,HERC5,HIST1H4A (includes others),HK2,HLA-E,HMGA1,HMGN2,HMOX1,HNRNPA1,HNRNPAB,HSPA14,HSPA1A/HSPA1B,HSPB1,HSPE1,ICAM1,ID1,ID2,ID3,IDH2,IER3,IFI35,IFIT1,IL1RAP,IL8,IMPA2,IREB2,IRF7,IRF9,ITGA6,ITGB1,ITM2B,KDR,KLF10,KLF4,KLF6,KRAS,LAMB2,LDHA,LIMS1,LIN28B,LSM4,LUM,LXN,MAD2L1,MAN2A1,MCM5,MCM7,MGAT1,MIF,MINA,MIR17HG,MITF,MKI67,MNT,MOGS,MRE11A,MRPL12,MSH2,MSN,MT1E,MTBP,MTF2,MTFR2,MTHFD1,MTHFR,MYC,MYO1C,NAP1L1,NBN,NCL,NDEL1,NDRG1,NDRG2,NFATC3,NGFRAP1,NME1,NME2,NOP56,OAS1,ODC1,OLIG1,PAICS,PAK2,PAM,PCNA,PFKFB3,PFKM,PFKP,PGAM1,PGK1,PHB,PLA1A,PLK1,PLP1,PMAIP1,PMP22,PNCK,PNO1,POLD1,POLR1B,PPP1CC,PPP1R15A,PRDX2,PRDX3,PREP,PRKACB,PRODH,PROM1,PSAT1,RAB40B,RAD51,RANBP1,RARA,RBBP4,RCC1,RHOB,RHPN1,ROCK2,RPL13,RPL21,RPL6,RPL7,RPS15A,RPS18,RPS27,RRM2,RRM2B,RRS1,RTN2,RUVBL2,SAT1,SCPEP1,SDCBP,SEPHS2,SERINC3,SFRP1,SGK1,SHMT1,SKP2,SLC1A5,SLC2A1,SLC2A3,SLC38A1,SLC3A2,SLC7A5,SMS,SNRPC,SNRPD1,SNRPN,SOD2,SOX6,SOX9,SPP1,ST3GAL1,STMN1,SUMO2,TAF1D,TAGLN2,TDG,TES,TF,TFDP1,THBS1,TMEM97,TMSB10/TMSB4X,TNC,TNFRSF10B,TP53,TP53I3,TPD52,TRIP12,TSPAN7,TXNIP,TYMS,UBE2C,UXT,VAMP3,VARS,XRCC3,YRDC,YY1,ZFP36,ZFP36L1,ZIC2
E2F1		transcription regulator	Inhibited	-3.152	2.11E-18	AMY2A,ATM,AURKA,AURKB,BAX,BCL2,BCL2L11,BIRC5,BMI1,BMP4,BNIP3,BOK,BRCA1,BUB3,CALD1,CALM1 (includes others),CASP7,CASP9,CBX5,CCNA2,CCNB1,CCNB2,CCND1,CCND3,CCNE1,CCNE2,CCNO,CD9,CDC25A,CDC45,CDC6,CDCA4,CDK1,CDK2,CDKN1A,CDKN2C,CDKN2D,CFLAR,CHEK1,CHEK2,CLIC1,COPS8,COX4I1,COX7C,CRABP2,CRYAB,CTNNBIP1,CTSB,DBF4,DDAH2,DGKI,DHFR,DLEU2,DNMT1,DUSP10,DUSP4,DUT,E2F2,E2F3,E2F4,ECT2,EED,EGR1,EIF3I,ENO2,EXOSC9,EYA4,EZH2,FBXO5,FEN1,FGFR1,FHIT,FOXM1,GM2A,GMNN,GPS2,H19,HADHA,HELLS,HES1,HIST1H2AC,HIST1H2BJ/HIST1H2BK,HIST1H4A (includes others),HIST2H2AA3/HIST2H2AA4,HLTF,HMGA1,HMGB1,HMGB2,HRK,HSPA1A/HSPA1B,HSPE1,ICAM1,ID3,JMY,KDR,KIAA0101,KIF23,KRT10,LACTB,MAD2L1,MAF,MAP3K14,MAP3K5,MCL1,MCM10,MCM2,MCM4,MCM5,MCM7,MEIS,MLH1,MMP16,MSH2,MT1G,MTHFD1,MYB,MYC,NCL,NCOA3,NDC80,NRIP1,NRP1,PA2G4,PAWR,PCNA,PHB,PMPCB,POLA2,POLD1,PPP1R13B,PPP1R8,PPT2,PRDM2,PRIM2,PRKDC,PSAT1,PSMD2,PTPN4,RACGAP1,RAD51,RANBP1,RBBP4,RBBP8,RBL1,RFC2,RFC3,RFC4,RPA3,RRM1,RRM2,RYBP,SIVA1,SLC3A2,SMC4,SNRPC,SOD2,STMN1,TAP1,TAPBP,TK1,TNFSF9,TOP2A,TOP2B,TP53,TP53BP2,TP53INP1,TRAF2,TYMS,UNG,UXT,VEGFB,ZFP36,ZNF160,ZNF22
E2F3	**-0.257**	transcription regulator	Inhibited	-2.868	8.36E-09	BIRC5,BMI1,BOK,CCNA2,CCNB1,CCND1,CCND3,CCNE1,CDC25A,CDC45,CDC6,CDCA4,CDK1,CDK2,CDKN1A,DHFR,E2F2,E2F3,EED,EZH2,FBXO5,MCM10,MCM2,MCM4,MCM5,MCM7,MIR17HG,MLH1,MT1G,MYB,MYC,PCNA,POLA2,PPP1R13B,PPP1R8,RAD51,RBL1,RRM1,STAG3,TK1,TOP2B,TP53BP2,UXT
TRIM24	**-0.312**	transcription regulator	Inhibited	-2.812	1.73E-02	AGRN,BST2,DDX58,DHX58,EIF5A,GBP2,GLUL,HERC6,IFI27L2,IFI35,IFI44,IFIH1,IFIT2,IFIT3,IFNGR1,IFNGR2,IRF7,IRF9,ISG15,KIAA0101,LGALS3BP,NMI,NUMA1,OAS1,PACSIN3,PARP12,PLEC,S100A11,SAMD9L,SHISA5,SOCS2,SPP1,STAT1,STAT2,TAP1,UBA7
**EZH2**	**-1.052**	transcription regulator	Inhibited	-2.698	2.72E-02	AADAT,ALPL,AMACR,APPBP2,AXIN2,BCL2,BCL2L11,BIRC3,BMP4,BRCA1,C15orf48,CCND1,CCND3,CCNE2,CD82,CDK6,CDKN1A,CPD,CTNNB1,CX3CL1,CXCL1,DENND2A,DKK1,EFNA1,EP300,ERBB2,EZH2,FBLN1,FHOD3,FOXF2,FRZB,FUCA1,GDF15,GSTM4,HOXB13,HOXB5,HOXB6,ICAM1,ICOSLG,IGFBP3,IL24,IL8,ITGA1,KIAA1199,KLF4,MAL2,MYC,MZF1,NME5,PAX3,PIM2,PKMYT1,PLA2G4A,PPP2CB,PRICKLE1,PTGES,PTGS2,RAC3,RAP1GAP,RBM5,SCAMP2,SCP2,SH3BP4,TBX3,TP53,TRIM38,WTAP,ZMYM4
E2F2	**-0.364**	transcription regulator	Inhibited	-2.682	9.89E-09	AMY2A,BCL2,BIRC5,BMI1,CCNA2,CCNB1,CCND1,CCND3,CCNE1,CDC25A,CDC45,CDC6,CDCA4,CDK1,CDKN1A,CDKN2C,CDKN2D,DHFR,E2F3,EED,EZH2,JMY,MCM10,MCM2,MCM4,MCM5,MCM7,MLH1,MT1G,MYB,MYC,PCNA,RAD51,RBL1,RRM2,TOP2B,TP53,UXT
TLX1		transcription regulator	Inhibited	-2.646	3.27E-01	CDC6,CHEK1,ERBB2,KDR,MCL1,MYB,NMI,PCNA,RAD51
KLF5		transcription regulator	Inhibited	-2.538	1.89E-01	ACADM,BIRC5,CCND1,CDK1,CDKN1A,CDT1,CTNNB1,E2F3,FAM110A,LAMA1,MYC,NOTCH1,PCNA,PPARG,RUNX1,TBC1D14,WFS1
TCF4		transcription regulator	Inhibited	-2.519	2.49E-02	BIRC5,CCND1,CDKN1A,DCT,DKK1,ELF4,ID2,IGFBP2,JUN,MIA,MITF,MYB,MYC,NCOR2,NOS2,PITX2,PSD3,SGK1,SOX9,TCF7L2,TYR,TYRP1
POU5F1		transcription regulator	Inhibited	-2.47	8.01E-02	ASH2L,BIRC5,BMI1,BRCA1,CCND1,CCNF,CDKN1A,DLX5,DNMT1,FOXA1,GADD45A,GOLGA6C (includes others),HOXB3,HOXC4,IER5L,ISL1,KLF2,KLF4,LHX2,MEF2A,MEIS1,MTF2,ONECUT1,ORAI1,OTP,PCGF6,PHB,PHC3,PMAIP1,RARA,SPP1,TBX3,TDRD7,TF,TP53,ZFHX3
MBD2		transcription regulator	Inhibited	-2.458	9.23E-02	AMY1A (includes others),ATP5I,BIRC3,BRCA1,CDKN1A,CENPH,CKS2,COX6C,G6PD,GSTP1,HK2,MEF2A,MGMT,NBR2,NEIL3,ORC6,PAK2,PLA2G16,PLK1,POLB,PTPN4,RELB
MYBL2		transcription regulator	Inhibited	-2.318	4.07E-03	BCL2,BCL2L11,BIRC3,BIRC5,CCNA2,CCNB1,CCNB2,CCND1,CDC25B,CDK1,CDK2,IGFBP5,IQGAP1,ITGB1,MAD1L1,MYC,PPP3CA,SLC1A5,TOP2A
SREBF2		transcription regulator	Inhibited	-2.291	1.53E-05	AACS,ABCA1,ACACA,ACSL1,ACSS2,ALDOC,CDKN1A,CYP51A1,DBI,EBP,ELOVL6,FABP5,FDFT1,FDPS,G6PD,GTF2I,HES6,HMGCR,HMGCS1,HSD17B1,IDH1,IDI1,INSIG1,LDLR,LSS,MSMO1,MVD,MVK,NSDHL,PLIN2,RDH11,SC5D,SCD,SQLE,SREBF1,TFF3,TM7SF2,TMEM97
MED13	0.258	transcription regulator	Inhibited	-2.121	3.82E-01	AACS,ACACA,CIB2,CLSTN3,ELOVL6,ENO1,GPD2,PPARG
GLI3		transcription regulator	Inhibited	-2.059	4.98E-01	BCL2,BMP4,CCND1,EMX2,GLI2,H2AFX,HOXD11,PTCH1,PVR,SALL1,SFRP1,SOX9,WNT5A

FLI1		transcription regulator	Inhibited	-2.037	3.22E-01	CCND1,CCND3,CTGF,DDX21,DNMT1,EGR1,ID2,IMP4,NHP2L1,NIP7,NOL6,POLR1D,RARB,SNRPB,SRGN,TCP1,TNC

Legend to Supplementary Table S2 A,B. Whole genome gene expression analysis of NFATc2_shRNA_86a and of two control transfectants (control shRNA_1 and _2) from Me71 was carried out as described in ref. 35, by single-color hybridization of RNAs performed on Illumina Bead Chip HumanHT-12_v4 Microarrays (Illumina). The expression profiles have been deposited in NCBI's Gene Expression Omnibus (GEO) with GSE accession number GSE101323. Differentially expressed genes in NFATc2 shRNA_86a transfectant compared to control-shRNA_1 (A) and control-shRNA-2 (B) transfectants were identified as described in ref 35 by BRBarray Tools (Vers.4.3.0) software. Ingenuity Pathway Analysis (IPA 8.5, www.ingenuity.com) was used to carry out "upstream regulator analysis" on differentially expressed genes. Upstream regulator analysis allows to identify the upstream transcriptional regulators that can explain the observed gene expression changes in the dataset. This computational tools returns results based on pvalues and Z score statistics. P values indicate the likelihood of the overlap between the genes in the dataset and those that are regulated by a predicted transcription factor. Negative Z score statistics (only those <-2 are considered significant and shown) are used to infer the "inhibited" the activation states of the predicted transcription factors.

Supplementary Table 3. **Antibodies for Western Blot analysis.**

Protein	Host	Catalog no	Company
NFATc2	mouse	610703	BD Transduction Laboratories
E-cadherin	mouse	610181	BD Transduction Laboratories
ZEB1	rabbit	3396	Cell Signaling
c-myc	rabbit	5605	Cell Signaling
Snail	rabbit	3879	Cell Signaling
Tri-Methyl-Histone H3 (Lys 27)	rabbit	9733	Cell Signaling
EZH2	rabbit	5246	Cell Signaling
N-cadherin	rabbit	13116	Cell Signaling
AXL	rabbit	4939	Cell Signaling
MITF clone C5	mouse	ab80651	Abcam
CTNNAL1	mouse	H00008727-B01P	Abnova
Twist	rabbit	sc-15393	Santa Cruz Biotechnology
ZEB2	mouse	sc-271984	Santa Cruz Biotechnology
FOXM1	rabbit	sc-500	Santa Cruz Biotechnology
ATF3	rabbit	sc-188	Santa Cruz Biotechnology
β-actin	rabbit	A2066	Sigma-Aldrich
vinculin	mouse	V9131	Sigma-Aldrich
α-tubulin	mouse	CP06	Calbiochem, Merck
DNA Topoisomerase I	mouse	556597	BD Pharmingen
STAT3	mouse	9139	Cell Signaling
pSTAT3	rabbit	4074	Cell Signaling
ERK 1/2	mouse	9107	Cell Signaling
pERK 1/2	rabbit	4370	Cell Signaling
ECL Anti-rat IgG, Horseradish Peroxidase-Linked species specific whole antibody from goat	goat	NA935	Ge Healthcare
anti-mouse IgM	rat	553435	BD Pharmingen
anti-mouse IgG	goat	NA931	Ge Healthcare
anti-rabbit IgG	rabbit	NA934	Ge Healthcare

Supplementary Table 4. Taqman Gene Expression Assays

Gene	Assay ID
AXL	Hs00242357_m1
ZEB1	Hs00232783_m1
MITF	Hs01117294_m1
NFATc2	Hs00905451_m1
SNAI1	Hs00195591_m1
CDH1	Hs01013958_m1
CDH2	Hs00983056_m1
EZH2	Hs00544830_m1
GAPDH	Hs99999905_m1
GUSB	Hs99999908_m1
HPRT1	Hs99999909_m1
TBX3	Hs01110714_m1
ITGB3	Hs00173978_m1
NRP1	Hs00826129_m1
CTGF	Hs01026927_g1
DDR2	Hs01025953_m1
FSTL1	Hs00907505_m1
AHNAK	Hs01102463_m1
PVRL3/NECTIN3	Hs00210045_m1
PRR16	Hs00706472_s1
KDR	Hs00911700_m1
DSP	Hs00950591_m1
QPCT	Hs01598354_m1

Supplementary Table 5. Stealth Select RNAi siRNa oligos

Target gene	assay ID	Sequence 5' to 3'	catalog no	Company
NFATc2	HSS107111	UCUACGUCAUCAAUGGGAAGAGAAA<br>UUUCUCUUCCCAUUGAUGACGUAGA	1299003	ThermoFisher Scientific
NFATc2	Hss107112	GGGUGACCAUUAAACAGGAGCAGAA<br>UUCUGCUCCUGUUUAAUGGUCACCC	1299003	ThermoFisher Scientific
c-myc	HSS106837	CAGCGACUCUGAGGAGGAACAAGAA<br>UUCUUGUUCCUCCUCAGAGUCGCUG	1299003	ThermoFisher Scientific
c-myc	HSS106839	CCAACAGGAACUAUGACCUCGACUA<br>UAGUCGAGGUCAUAGUUCCUGUUGG	1299003	ThermoFisher Scientific
FOXM1	HSS103712	CCCUGCCCAACAGGAGUCUAAUCAA<br>UUGAUUAGACUCCUGUUGGGCAGGG	1299003	ThermoFisher Scientific
FOXM1	HSS103713	GCCAUGAUACAAUUCGCCAUCAACA<br>UGUUGAUGGCGAAUUGUAUCAUGGC	1299003	ThermoFisher Scientific
EZH2	HSS176652	GACCACAGUGUUACCAGCAUUUGGA<br>UCCAAAUGCUGGUAACACUGUGGUC	1299003	ThermoFisher Scientific
EZH2	HSS176653	GAGCAAAGCUUACACUCCUUUCAUA<br>UAUGAAAGGAGUGUAAGCUUUGCUC	1299003	ThermoFisher Scientific

Supplementary Table 6. Antibodies for immunohistochemistry

Protein	Host	Catalog no	Company
NFATc2	rabbit	HPA024369	Sigma-Aldrich
AXL	goat	AF154	R&D Systems
MITF	mouse	M3621	DAKO/Agilent
EZH2	rabbit	5246	Cell Signaling
ZEB1	rabbit	sc-25388	Santa Cruz Biotechnology
N-cadherin	mouse	M3613	DAKO
